# Rapid extraction of trace benzene by a crown-ether-based metal-organic framework

**DOI:** 10.1093/nsr/nwae342

**Published:** 2024-10-03

**Authors:** Zhonghang Chen, Peiyu Fang, Jiangnan Li, Xue Han, Wenhao Huang, Wenyue Cui, Zhiwei Liu, Mark R Warren, David Allan, Peng Cheng, Sihai Yang, Wei Shi

**Affiliations:** Frontiers Science Center for New Organic Matter, Key Laboratory of Advanced Energy Materials Chemistry (MOE), and State Key Laboratory of Advanced Chemical Power Sources, College of Chemistry, Nankai University, Tianjin 300071, China; Beijing National Laboratory for Molecular Sciences, College of Chemistry and Molecular Engineering, Peking University, Beijing 100871, China; Beijing National Laboratory for Molecular Sciences, College of Chemistry and Molecular Engineering, Peking University, Beijing 100871, China; College of Chemistry, Beijing Normal University, Beijing 100875, China; Frontiers Science Center for New Organic Matter, Key Laboratory of Advanced Energy Materials Chemistry (MOE), and State Key Laboratory of Advanced Chemical Power Sources, College of Chemistry, Nankai University, Tianjin 300071, China; Frontiers Science Center for New Organic Matter, Key Laboratory of Advanced Energy Materials Chemistry (MOE), and State Key Laboratory of Advanced Chemical Power Sources, College of Chemistry, Nankai University, Tianjin 300071, China; Beijing National Laboratory for Molecular Sciences, College of Chemistry and Molecular Engineering, Peking University, Beijing 100871, China; Diamond Light Source, Harwell Science and Innovation Campus, Oxfordshire OX11 0DE, UK; Diamond Light Source, Harwell Science and Innovation Campus, Oxfordshire OX11 0DE, UK; Frontiers Science Center for New Organic Matter, Key Laboratory of Advanced Energy Materials Chemistry (MOE), and State Key Laboratory of Advanced Chemical Power Sources, College of Chemistry, Nankai University, Tianjin 300071, China; Haihe Laboratory of Sustainable Chemical Transformations, Tianjin 300192, China; Beijing National Laboratory for Molecular Sciences, College of Chemistry and Molecular Engineering, Peking University, Beijing 100871, China; Frontiers Science Center for New Organic Matter, Key Laboratory of Advanced Energy Materials Chemistry (MOE), and State Key Laboratory of Advanced Chemical Power Sources, College of Chemistry, Nankai University, Tianjin 300071, China

**Keywords:** metal-organic frameworks, crown ether, triangular channel, benzene/cyclohexane separation, crystal structure

## Abstract

Due to almost identical boiling points of benzene and cyclohexane, the extraction of trace benzene from cyclohexane is currently performed *via* the energy-intensive extractive distillation method. Their adsorptive separation by porous materials is hampered by their similar dimensions. Metal-organic frameworks (MOFs) with versatile pore environments are capable of molecular discrimination, but the separation of trace substrates in liquid-phase remains extremely challenging. Herein, we report a robust MOF (NKU-300) with triangular channels decorated with crown ether that can discriminate trace benzene from cyclohexane, exhibiting an unprecedented selectivity of 8615(10) for the mixture of benzene/cyclohexane (v/v = 1/1000). Remarkably, NKU-300 demonstrates exceptional selectivities for the extraction of benzene from cyclohexane over a wide range of concentrations of 0.1%–50% with ultrafast sorption kinetics and excellent stability. Single-crystal X-ray diffraction and computational modelling reveal that multiple supramolecular interactions cooperatively immobilise benzene molecules in the triangular channel, enabling superior separation performance. This study will promote the application of advanced sorbents with tailored binding sites for challenging industrial separations.

## Teaser

A robust metal-organic framework (NKU-300) with triangular channels decorated with crown ether can discriminate trace benzene from cyclohexane, exhibiting an unprecedented selectivity of 8615(10) for the mixture of benzene/cyclohexane (v/v = 1/1000).

## INTRODUCTION

Cyclohexane is critical for the synthesis of a wide range of materials, such as resins, nylon-6, and nylon-6,6, with a global annual market of >$20 billion [1]. It is produced by hydrogenation of benzene and the downstream purification requires the removal of trace benzene (<1%) from cyclohexane as trace benzene can significantly interfere with the sequential polymerization [2]. However, due to their close boiling points (benzene: 80.1°C; cyclohexane: 80.7°C) and azeotropic tendencies, the extraction of trace benzene from cyclohexane is extremely challenging and is currently performed in industry by the energy-intensive extractive and azeotropic distillation methods [3,4]. While catalytic hydrogenation can further reduce the residual benzene to ppm level, this process operates at high temperature/pressure conditions and requires noble metal catalysts [4].

**Figure 1.**
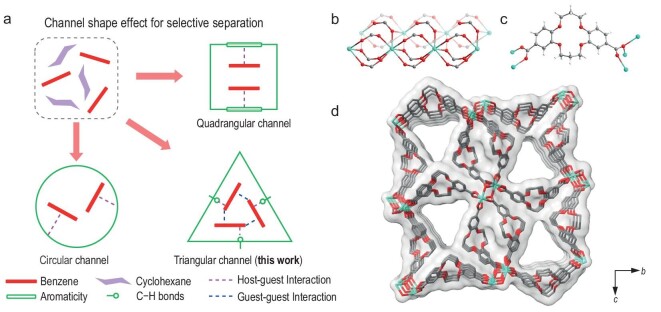
The design of benzene-adsorbed MOF and the crystal structure of NKU-300. (a) Comparison of binding of benzene molecules in quadrangular, triangular and circular channels. (b) Side view of the 1D {EuL}_∞_ coordination chain. (c) Coordination mode of L^2−^. (d) View of the framework structure of NKU-300 along the *a* axis; turquoise, Eu; red, O; gray, C atoms. H atoms are omitted for clarity.

Adsorptive separation is a promising alternative approach due to its ease of operation, low energy consumption, and excellent recyclability [5–8]. However, zeolites and activated carbons can hardly discriminate between these two molecules due to their similar dimensions (benzene: 7.123 × 6.869 × 3.401 Å^3^; cyclohexane: 7.118 × 6.757 × 4.876 Å^3^). Recent developments in non-porous adaptive crystals (NACs) [9,10], supramolecular coordination complexes (SCCs) [11,12], metal-organic frameworks (MOFs) [13–15], and covalent organic frameworks (COFs) [16] have witnessed many breakthroughs in gas separation [17–20]. NACs and SCCs can separate benzene and cyclohexane due to the highly confined host–guest interactions [21], but their performance is often limited by the hindered diffusion of the substrates. MOFs have emerged as promising sorbents for this separation [22–28]. Optimization of the pore chemistry through the rational application of metal···π, C–H···π, and π···π interactions and molecular sieving by spatial confinement to discriminate these two substrates is key to improving the separation efficiency [29]. Most of the studied MOFs have quadrangular or circular pores to bind benzene molecules by forming specific host–guest interactions, while intermolecular guest–guest interactions are usually weak due to the flexibility of packing of benzene molecules [23]. In contrast, in a triangular channel, both host–guest and guest–guest interactions (primarily through C–H···π interactions) are strongly confined with high rigidity, which is reminiscent of solid benzene, thus promoting further adsorption through efficient packing (Fig. 1a). To date, most studies have focused on the equimolar mixture with a selectivity up to 166; however, the practical mixture contains only *ca.* <0.5% benzene, making the selective adsorption of trace benzene extremely challenging. To the best of our knowledge, the extraction of trace benzene in a mixture of benzene/cyclohexane (v/v = 1/1000) by a sorbent material has not been achieved. Furthermore, the intrinsic trade-off between molecular diffusion and adsorption selectivity limits their practical potential [30].

Here, we demonstrate a promoted adsorption mechanism in a robust MOF (NKU-300) based on a V-shaped ligand (Fig. S1 in Supplementary data) with triangular channels decorated with crown ether. Unlike the conventional quadrangular or cylindrical channels in MOFs, the triangular channels (size of 10.0 × 10.0 × 10.7 Å^3^; Fig. S2) of NKU-300 provide an optimal binding environment specifically for benzene but not for cyclohexane (Fig. 1), resulting in a record-high selectivity of 8615(10) for the liquid-phase separation of benzene/cyclohexane (v/v = 1/1000) under ambient conditions. High purity cyclohexane (99.99%) can be obtained from only one cycle of adsorption and an exceptional separation productivity of 0.296 kg kg^−1^ h^−1^ was achieved. NKU-300 also exhibits excellent stability over 20 cycles of adsorption-desorption based separations, outperforming the benchmark materials (Tables S1 and S2). Single-crystal X-ray diffraction (SCXRD) analysis of NKU-300 immersed in a mixture of benzene/cyclohexane (v/v = 1/100) shows that trimers of benzene molecules confined in the triangular channels interact with the methylene sites of crown ether *via* multiple C–H···π interactions, whereas cyclohexane molecules are excluded from this channel. *In situ* synchrotron SCXRD reveals the sequential binding of each adsorption site. Density functional theory (DFT) calculations suggest that the immobilization of the first benzene molecule in the pore can promote further accommodation of benzene molecules in NKU-300, resulting in accelerated adsorption kinetics. Molecular dynamics (MD) calculations further confirm the observed high selectivity and fast kinetics.

## RESULTS AND DISCUSSION

The ligand H_2_L (H_2_L = 7,8,16,17-tetrahydro-6*H*,15*H*-dibenzo[*b, i*][1,4,8,11]tetraoxacyclotetradecine-2,12-dicarboxylic acid; Fig. S1) containing dibenzo-14-crown-4 and carboxylic groups was synthesized *via* the Williamson ether route [31]. Single crystals of NKU-300, Eu(L)_1.5_·G*_x_* (G = guest), were synthesized by the solvothermal reaction of Eu(OAc)_3_·3H_2_O (OAc = acetate) and H_2_L in an acidic (HNO_3_) mixture of *N,N*-dimethylformamide (DMF) and water. SCXRD shows that NKU-300 crystallizes in the monoclinic space group *P*2_1_/*m*. The Eu(III) centre is seven-coordinated with oxygen donors [Eu-O = 2.318(2)–2.474(2) Å] in a distorted decahedron geometry (Fig. 1b). Each L^2−^ ligand acts as a *μ*_5_-bridge to connect five Eu(III) ions through two types of bidentate binding modes (*μ*_2_-*η*^1^:*η*^1^; *μ*_3_-*η*^2^:*η*^1^; Fig. 1c), forming one-dimensional (1D) triangular channels decorated with crown ether running through the 3D framework, resulting in ∼20% voids (Fig. 1d).

**Figure 2.**
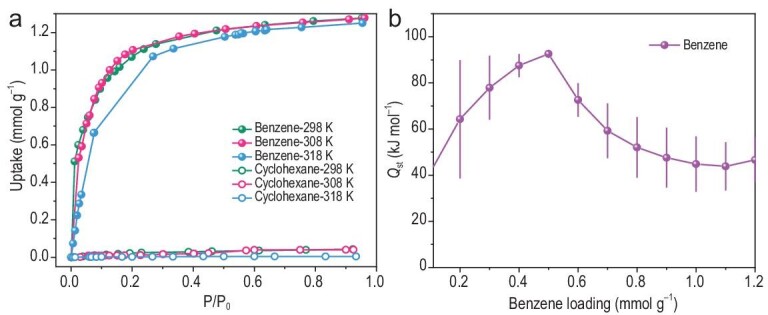
Single-component adsorption isotherms of benzene and cyclohexane vapour (a) and the adsorption enthalpy of benzene (b) in desovlated NKU-300.

Powder X-ray diffraction (PXRD) and infrared spectroscopy (IR) confirmed the bulk purity and excellent stability of NKU-300 in common organic solvents and aqueous solutions (pH 2–12) (Figs S3–S5). Thermogravimetric analysis (TGA) shows that NKU-300 is stable to 400°C (Fig. S6) with the retention of the framework upon desolvation confirmed by PXRD (Fig. S7). Desolvated NKU-300 shows a notable uptake of benzene vapour (1.28 mmol g^−1^) but negligible uptake of cyclohexane at 298 K (Fig. 2, Figs S8–S10), thus demonstrating a great potential for their separation.

**Figure 3.**
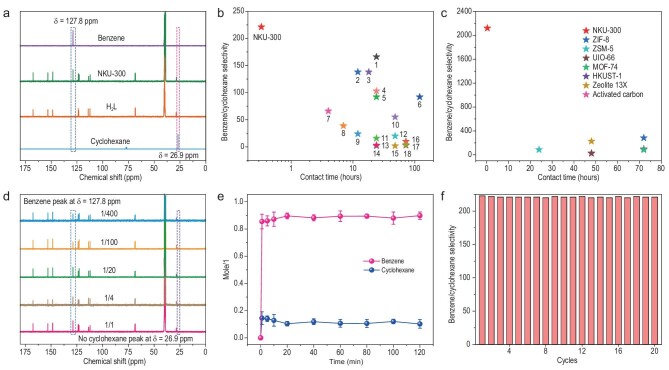
Performance of liquid-phase separation of benzene/cyclohexane by NKU-300. (a) Separation selectivity of benzene/cyclohexane evaluated by ^13^C NMR spectra. (b) Comparison of selectivities of the benzene/cyclohexane (equimolar mixture) of state-of-the-art materials. Contact time refers to the soaking time of the materials in the mixture of benzene/cyclohexane; 1–18 refer to MFM-300(Sc), BNF-2, MAF-stu-13, MFM-300(Cr), MFM-300(In), [Zn_4_(EgO_2_)_2_(tdc)_2_(dabco)], Carborane Metallacage, Hybrid[3]arene, L^H^-Au_10_S_4_-Cl, Zeolite 13X, [Li_2_Zn_2_(NO_2_-bdc)_3_(bpy), ZnL, UiO-66, ZSM-5, activated carbon, ZIF-8, MOF-74, HKUST-1, respectively. (c) Comparison of the selectivity of the benzene/cyclohexane (v/v = 1/100) of NKU-300 with benchmark sorbents. (d) Liquid-phase separation of benzene/cyclohexane (v/v = 1/1, 1/4, 1/20, 1/100, and 1/400) evaluated by ^13^C NMR spectra. (e) Time-dependent solid-liquid sorption profiles for the mixture of benzene/cyclohexane (v/v = 1/1000) in NKU-300. (f) Cyclic separation of the mixture of benzene/cyclohexane (v/v = 1/1) over 20 cycles (data derived from ^1^H NMR spectra).

Since liquid-phase purification is considered more economical and energy-efficient than gas-phase separation [30], the activated NKU-300 was immersed in an equimolar liquid mixture of benzene/cyclohexane for 30 minutes at 298 K, followed by filtration and brief drying in air. The sample was then digested in DCl/DMSO-*d*_6_ (DMSO = dimethylsulfoxide) and analyzed by ^13^C nuclear magnetic resonance (NMR) spectroscopy, which confirmed the preferential uptake of benzene over cyclohexane by NKU-300 with a remarkable benzene/cyclohexane selectivity of 221 (Fig. 3a, Figs S11 and S12), outperforming all benchmark materials (Fig. 3b, Tables S1 and S2). More importantly, NKU-300 can extract trace amounts of benzene from cyclohexane at various concentrations (v/v of benzene/cyclohexane = 1/1, 1/4, 1/20, 1/100, 1/400, and 1/1000) (Fig. 3c and d, Figs S12–S14), and an unprecedented selectivity of 8615(10) for the mixture of 1/1000 benzene/cyclohexane (i.e. benzene concentration <0.1%, Figs S12 and S15) was achieved, representing the first example of extracting benzene at such low concentration by a sorbent material. The kinetic plots for the liquid phase separation of the benzene/cyclohexane mixture (v/v = 1/1000) showed that an exceptional selectivity of >5889 was reached within 1 minute (Fig. 3e), and within 20 minutes, the adsorption reached equilibrium with selectivity of 8643(18) (Figs S16–S21). The excellent separation performance for a mixture of liquid benzene/cyclohexane (v/v = 1/1000) by a fixed-bed packed with NKU-300 has also been confirmed (Fig. S22). The excellent separation performance of NKU-300 also retained over 20 consecutive adsorption-desorption cycles (Fig. 3f, Figs S23–S27), demonstrating its great practical potential.

**Figure 4.**
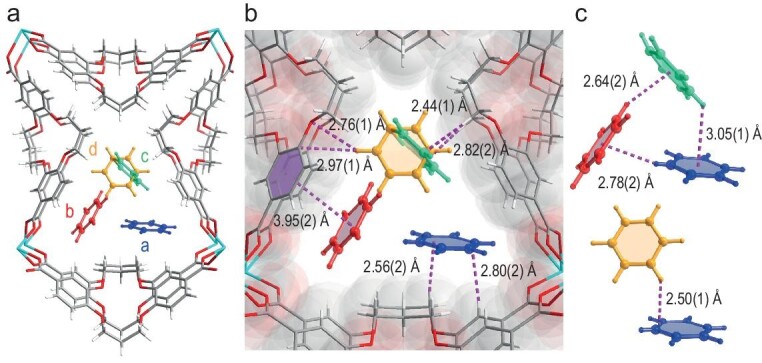
Crystal structure of benzene@NKU-300. (a) The SCXRD structure of benzene@NKU-300 (sites a, b, c, d). (b) The host–guest interactions of benzene and NKU-300. (c) The interactions between adsorbed benzene molecules.

To elucidate the molecular mechanism behind the superior selectivity and fast kinetics, benzene-loaded NKU-300 was prepared by immersing the activated samples in pure benzene (benzene@NKU-300) followed by drying and analysis using SCXRD. The NKU-300 framework was fully retained upon loading with benzene (Figs S28 and S29), and four distinct adsorption sites (a, b, c, and d with occupancies of 0.58, 0.52, 0.44, and 0.42, respectively) were identified for benzene@NKU-300, corresponding to a total uptake of 1.34 mmol g^−1^, which is in agreement with the isothermal uptake (1.28 mmol g^−1^). Sites a, b, and c are located symmetrically at both sides of the mirror plane and aligned parallel to the channel's elongation direction (Fig. S30). Whereas for site d, the single benzene molecule is oriented perpendicular to the cross section of the channel (Fig. 4a). The adsorbed benzene molecules are primarily stabilized by multiple host–guest interactions, i.e. C–H_framework_···π_benzene_ with H···C distances between 2.44(1)–2.82(2) Å (Fig. 4b). These interactions were also evidenced by Fourier transform infrared (FT-IR) studies, where the *ν*_s_(CH_2_) bands of the crown ether group (2815–2708 cm^−1^) are blue-shifted to 2831–2716 cm^−1^ upon adsorption of benzene (Fig. S31). In addition, guest–guest interactions were also observed with the adsorbed benzene molecules at sites a, b, and c exhibiting C–H···π interactions [H···centroid of benzene distances of 2.50(1)–3.05(1) Å] with each other in a manner similar to solid benzene (H···centroid of benzene distance of 2.83 Å, Fig. S32) [32,33] and an additional C–H···π interaction was also observed between site a and site d [H···C distance of 2.50(1) Å] (Fig. 4c). These aforementioned host–guest and guest–guest interactions, facilitated by the abundant aliphatic C–H bonds on the crown ether, collectively confine the benzene molecules to the triangular channels of NKU-300 [34,35].

It is worth noting that SCXRD analysis of NKU-300 immersed in benzene/cyclohexane mixture (v/v = 1/100) revealed a structure highly consistent with benzene@NKU-300 (Fig. S33), again confirming the selective adsorption of benzene during the competitive adsorption by NKU-300.

**Figure 5.**
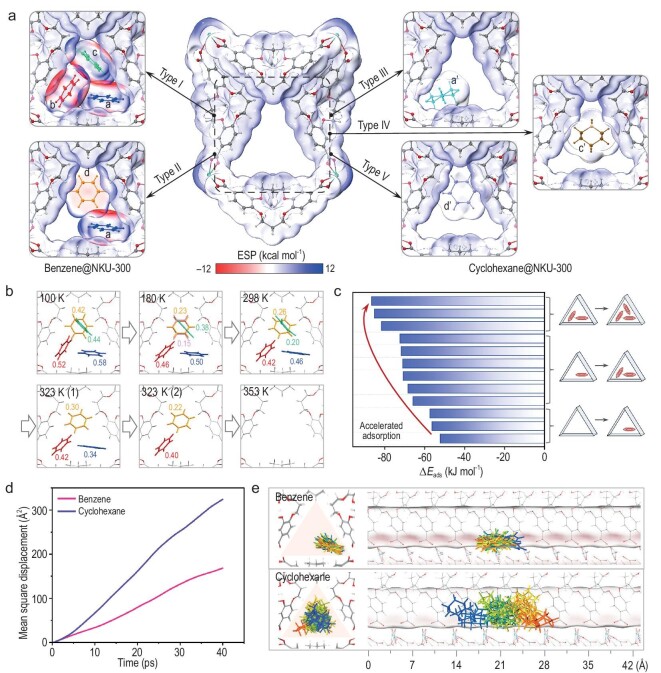
Computational studies and *in situ* SCXRD structures. (a) Electrostatic potential distribution of adsorption configurations of benzene (left, types Ⅰ and Ⅱ) and cyclohexane molecules (right, types Ⅲ, Ⅳ, and Ⅴ). (b) *In situ* SCXRD structures of desorption of benzene@NKU-300 from 180 to 353 K. Detailed measurement procedure is 180 K → 298 K → 323 K (1) and vacuum → 323 K (2) and Ar 200 mbar → 353 K; 100 K is an independent dataset of benzene@NKU-300. (c) The binding energy of distinct adsorption processes in benzene@NKU-300. (d) Graph showing the mean square displacements along the channel of NKU-300 for benzene and cyclohexane over 40 ps; data obtained *via* MD simulation using the NVT ensemble at 300 K. (e) The positions of benzene and cyclohexane sampled along the front and side view of the channel of NKU-300.

DFT calculations were performed based on the crystal structure of benzene@NKU-300 to obtain the electrostatic potential distribution. The triangular channel can be saturated by three benzene molecules at sites a, b, and c or two benzene molecules at sites a and d (Fig. 5a, type Ⅰ and Ⅱ). The electrostatic potential reveals the compact spatial arrangement of benzene due to its matched shape and size to the channel, thus minimizing the overall system energy.

To determine the strength of each adsorption site, *in situ* synchrotron SCXRD data were collected during the desorption of benzene from benzene@NKU-300 by gradual heating of the sample from 180 to 353 K under dynamic vacuum (Figs S34–S43 and Tables S7–S19) [36]. The benzene molecule at site d was the first to desorb at 180 K (Fig. 5b); as the temperature increased to 298 K, the occupancy of benzene at sites c and d gradually decreased from 0.76 to 0.46 (Fig. 5b). Interestingly, at 323 K, benzene molecules gradually migrate from sites c and a to sites d and b, respectively (Fig. 5b). At 353 K (i.e. the boiling point of benzene), a complete desorption of all sites was observed (Fig. 5b). These data uncovered the order of strength of benzene binding as d < c < a < b, which is in excellent agreement with the DFT calculation for the binding energy [site d (−58.6 kJ mol^−1^) < c (−67.1 kJ mol^−1^) < a (−70.4 kJ mol^−1^) < b (−71.5 kJ mol^−1^)] (Fig. S44 and Tables S20–S22). The enthalpy of adsorption (*Q*_st_) for benzene in NKU-300 is ∼90 kJ mol^−1^ at low surface coverage, and it gradually decreases to 43 kJ mol^−1^ upon increasing surface coverage (Fig. 2). DFT calculation revealed a binding energy of −105 kJ mol^−1^, confirming the strong affinity between the framework and benzene.

The binding energy during stepwise adsorption is shown in Fig. 5c (Fig. S44 and Table S21) and the increasing tendency of the binding energy implies that the subsequent adsorption processes are enhanced by the population of the previous adsorption sites. The adsorption kinetics of benzene vapor was measured, showing accelerated adsorption at its onset (Fig. S45). The low activation barrier of 19.7 kJ mol^−1^ facilitates the diffusion of benzene along the channel (Figs S46–S48 and Table S23). Therefore, the guest–guest C–H···π interaction plays an important role in increasing the binding energy of benzene molecules to expedite the adsorption process.

In contrast, the triangular channel of NKU-300 can accommodate only one cyclohexane molecule in three configurations from the optimized structure of cyclohexane@NKU-300 (types Ⅲ, Ⅳ, and Ⅴ in Fig. 5a). Analysis of electrostatic potential indicates that none of these sites forms significant interactions with NKU-300 (Fig. 5a).

Thus, the triangular channel of NKU-300 can accommodate packing of benzene molecules similar to that of solid benzene, while excluding the slightly wider cyclohexane molecule. Importantly, the interior of triangular channels is decorated with crown ether groups, facilitating the selective binding of benzene molecules *via* multiple C–H···π interactions, which promotes exceptional benzene/cyclohexane selectivity and separation performance. Thus, both the shape and size of the triangular channel, as well as the suitable pore interior environment, are important factors for the excellent separation performance of NKU-300.

MD simulations at 300 K were performed to investigate the diffusion of benzene and cyclohexane along the channel of NKU-300 (Fig. 5d and e, and Movies S1–S4). Benzene was found to oscillate between the strong binding sites a and b, which is in excellent agreement with the DFT (Tables S20 and S22) and *in situ* synchrotron SCXRD analysis (Fig. 5b). In contrast, the diffusion trajectory of cyclohexane shows no configuration or site preference due to its slightly larger molecular volume (Fig. S49) [37]. Benzene tends to stick together as clusters in the channel due to the strong guest–guest interactions (Movies S5 and S6). Thus, both host–guest and guest–guest interactions promote the accumulation of benzene in the channel.

Rapid and facile detection of benzene is also an important task due to toxicity hazards even at trace levels. The luminescent sensing properties of NKU-300 for trace benzene in different solvents was performed (Figs S50–S59 and Table S24). The emission intensity of NKU-300 dispersed in cyclohexane, DMF, ethanol, and acetonitrile notably decreased with the incremental addition of benzene because of the competitive absorption of ultraviolet light [38]. In contrast, under excitation at 312 nm, a significant enhancement of the emission intensity of NKU-300 was observed upon addition of benzene in water, suggesting the formation of benzene@NKU-300, which suppresses nonradiative decay through multiple hydrogen bonding [39]. The sensing process was completed in only 10 seconds with a limit of detection of 64.7 ppb, demonstrating the promising potential of NKU-300 for sensing benzene at low concentrations.

## CONCLUSIONS

We report a robust sorbent material, NKU-300, with unique triangular channels and suitable aperture sizes that exhibit an exceptional performance in the extraction of trace benzene from cyclohexane in the liquid-phase under ambient conditions. NKU-300 shows an unprecedented selectivity of 8615(10) for benzene/cyclohexane mixture (v/v = 1/1000). The practical potential of NKU-300 is further enhanced by its remarkable stability over repeated cycles and fast sorption kinetics. Structural analysis coupled with modelling has provided key insights into the optimal host–guest and guest–guest interactions which are dominated by C–H···π interactions, and underpin the superior performance in this challenging separation. More interestingly, NKU-300 shows a highly sensitive and unusual turn-on fluorescence response upon contact with trace benzene (64.7 ppb) in water within 10 seconds, thus it can also act as an excellent sensor for trace benzene in polluted water prior to extraction.

## METHODS

### Characterization methods

PXRD measurements were conducted on a Rigaku SmartLab SE X-ray diffractometer with Cu-K*α* radiation. TGA was performed by using a Mettler Toledo TGA 2 analyzer under a N_2_ atmosphere in the range of 40–800°C with a heating rate of 10° min^−1^. FT-IR spectra were collected using a Bruker Alpha ATR-FTIR spectrometer. The NMR spectra were recorded on a Bruker 400 AV spectrometer at 400 MHz (^1^H NMR) and 101 MHz (^13^C NMR). The high-resolution mass spectra (HRMS) were recorded on an Agilent 6520 Q-TOF liquid chromatography mass spectrometer. UV-vis absorption spectra were measured on a SHIMADZU UV-2600 spectrophotometer. Luminescence spectra and luminescence lifetimes were recorded on an Edinburgh FS5 fluorescence spectrophotometer equipped with Xenon lamp and pulsed flash lamps.

### Synthesis of NKU-300

A mixture of H_2_L (0.010 g, 0.026 mmol), Eu(OAc)_3_·3H_2_O (0.030 g, 0.078 mmol), DMF (2.5 mL), and deionized H_2_O (1 mL) was put into a Teflon-lined autoclave (20 mL), and then 60 μL HNO_3_ (1 M) was added. The autoclave was cooled slowly to room temperature after heating at 160°C for 48 hours. Colorless rod crystals of NKU-300 were collected by filtration, washed with DMF, deionized water, and dried in air (yield: 43%). Elemental analysis for C_30_H_27_EuO_12_ calcd. (%): C, 49.3; H, 3.72; found (%): C, 49.6; H, 3.54.

### Liquid-phase benzene/cyclohexane separation experiments

The liquid-phase benzene/cyclohexane separation batch experiments were conducted at 298 K. 13 mg of activated NKU-300 was soaked in 2 mL (v/v = 1/1, 1/4, 1/20, 1/100) and 20 mL (v/v = 1/400, 1/1000) of the mixture of benzene/cyclohexane in a sealed vial. The crystals were collected by filtration and dried in air to remove the surface-adsorbed benzene or cyclohexane molecules, and then was digested in DCl/DMSO-*d*_6_ for the measurement of ^1^H NMR and ^13^C NMR spectra. The benzene/cyclohexane relative uptake by NKU-300 was measured by the ratio of the integrals of the peaks corresponding to benzene and cyclohexane in ^1^H NMR spectra, taking into account the number of protons, the integrals of benzene peak were measured by the ratio of the benzene to H_2_L. All batch separation experiments were performed with three replicates.

The benzene/cyclohexane selectivity (*S*) is calculated by the equation below:


\begin{eqnarray*}
S = \frac{{a/b}}{{{{a}_0}/{{b}_0}}},
\end{eqnarray*}

where *a* and *b* are the molar ratio of adsorbed benzene and cyclohexane, respectively, which are determined by the ratio of the integrals of the peaks in NMR spectra. *a*_0_/*b*_0_ is the molar ratio of benzene and cyclohexane in the mixed solvent. The purity of the resultant cyclohexane after the removal of benzene was determine by a gas chromatography-mass spectrometer instrument (Agilent 7890B GC-5977B MSD).

## Supplementary Material

nwae342_Supplemental_Files

## Data Availability

Crystal structure data are available in the Cambridge Structural Database under deposition numbers 2320896, 2320897, 2320898, 2320924, 2320926, 2320932, 2320933, 2320934, 2321059, 2321060, 2321066, 2321067, 2321068, and 2320931. All other data are available from the corresponding authors upon request.
