# Supplementary material for: Rapid extraction of trace benzene by a crown-ether-based metal-organic framework
Source: Natl Sci Rev. 2024 Oct 3;11(12):nwae342. doi: 10.1093/nsr/nwae342 (PMC11562822; doi:10.1093/nsr/nwae342)
Supplement: nwae342_Supplemental_Files [file nwae342_supplemental_files.zip › SI-final-R1.pdf]

Supplementary Materials

Rapid extraction of trace benzene by a crown-ether-based metal-organic framework

Zhonghang Chen *et al*.

Corresponding authors: Wei Shi, shiwei@nankai.edu.cn; Sihai Yang, Sihai.Yang@pku.edu.cn

The PDF file includes:

Experimental section
Figs. S1 to S59
Tables S1 to S24
References

Other Supplementary Materials for this manuscript include the following:

Movies S1 to S6

Synthesis of the H_2L

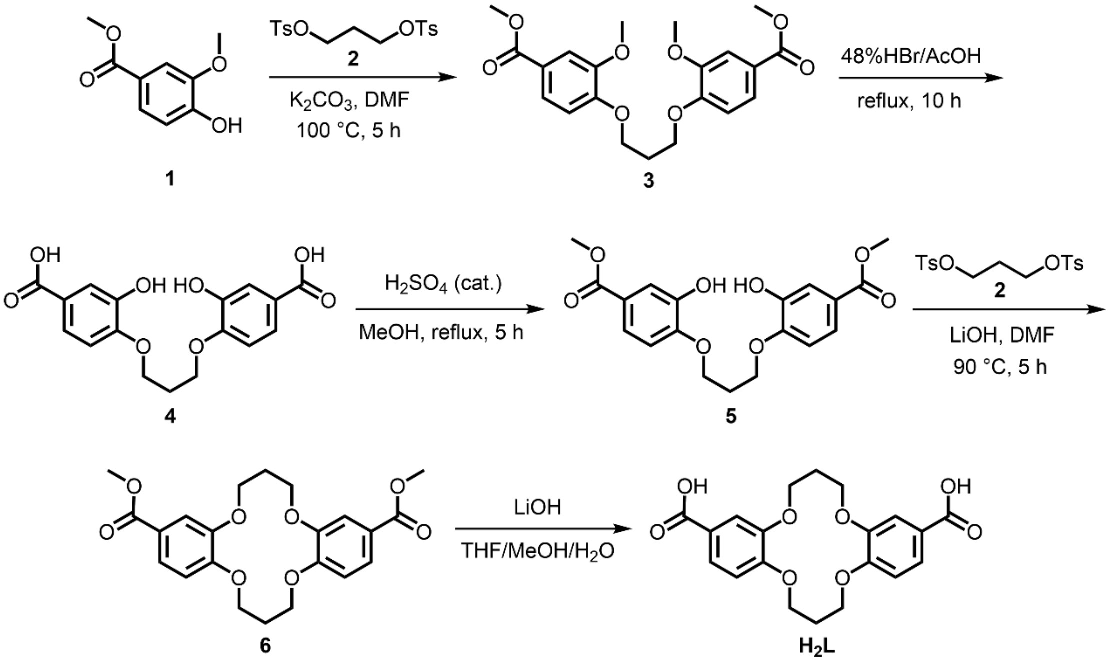

Synthesis of 3. 2 was synthesized according to the literature [1]. A mixture of **1** (40.08 g, 220 mmol), K_2CO_3 (30.41 g, 220 mmol), **2** (42.29 g, 110 mmol) and DMF (230 mL) was purged with argon to degas. The mixture was allowed to stir at 100 °C for 5 h. The reaction mixture was then cooled to room temperature and poured into ice-cold water. The precipitate was filtrated and dried under vacuum at 110 °C overnight. **3** was obtained as a white solid (41.37 g, 93%).

^{1}H NMR (400 MHz, $CDCl_3$) δ 7.63 (dd, *J* = 2.8 2.4 Hz, 2H, Ar-H), 7.52 (d, *J* = 2.8 Hz, 2H, Ar-H), 6.84 (d, *J* = 8.0 Hz, 2H, Ar-H), 4.29 (t, *J* = 9.6 Hz, 4H, $C\underline{H}_2CH_2C\underline{H}_2$), 3.86 (s, 6H, COOMe), 3.85 (s, 6H, OMe), 2.38-2.34 (m, 2H, $CH_2C\underline{H}_2CH_2$).

^{13}C NMR (101 MHz, $CDCl_3$) δ 166.9, 152.3, 148.9, 123.9, 122.8, 112.8, 111.9, 65.4, 55.7, 51.7, 28.9.

HRMS-ESI (m/z): calcd for $[C_{21}H_{24}O_8, M+H]^+$: 405.1550, Found: 405.1543.

Synthesis of 4. 3 (4.85 g, 12 mmol) in a mixture of AcOH (120 mL) and HBr (100 mL, 48%) was purged with argon to degas. The mixture was refluxed for 10 h and allowed to cool to room temperature and poured into ice-cold water. The precipitate was filtrated and dried under vacuum at 110 °C for 12 h. **4** was obtained as a white solid (3.43 g, 82%).

^{1}H NMR (400 MHz, DMSO-d_6) δ 12.51 (s, 2H, COOH), 9.26 (d, *J* = 12.8 Hz, 2H, OH), 7.40 (dd, *J* = 14.4, 9.1 Hz, 4H, Ar-H), 7.03 (dt, *J* = 14.6, 7.5 Hz, 2H, Ar-H), 4.24 (dq, *J* = 12.1, 6.1 Hz, 4H, $C\underline{H}_2CH_2C\underline{H}_2$), 2.23 (dt, *J* = 12.9, 6.20 Hz, 2H, $CH_2C\underline{H}_2CH_2$).

^{13}C NMR (101 MHz, DMSO-d_6) δ 167.6, 151.2, 146.7, 123.6, 122.1, 116.6, 112.7, 65.4, 29.0.

HRMS-ESI (m/z): calcd for [$C_{17}H_{16}O_8$, M+H]$^+$: 349.0924, Found: 349.0920.

Synthesis of 5. A solution of **4** (5.22 g, 15 mmol) in anhydrous MeOH (150 mL) and conc. H_2SO_4 (2.5 mL) was stirred under reflux for 5 h. After cooling to room temperature, the reaction was neutralized with aqueous $NaHCO_3$. The precipitate was filtered and dried under vacuum at 110 °C for 12 h. **5** was obtained as an offwhite solid (5.36 g, 95%).

Melting point: 240-242 ºC.

^{1}H NMR (400 MHz, DMSO-d_6) δ 9.33 (s, 2H, OH), 7.42 (dd, *J* = 8.4, 2.1 Hz, 2H, Ar-H), 7.38 (d, *J* = 2.2 Hz, 2H, Ar-H), 7.05 (d, *J* = 8.5 Hz, 2H, Ar-H), 4.24 (q, *J* = 5.5, 4.7 Hz, 4H, $C\underline{H}_2CH_2C\underline{H}_2$), 3.79 (s, 3H, COOMe), 2.22 (p, *J* = 6.2 Hz, 2H, $CH_2C\underline{H}_2CH_2$).

^{13}C NMR (101 MHz, DMSO-d_6) δ 166.5, 151.5, 146.8, 122.4, 122.0, 116.3, 112.8, 65.4, 52.2, 28.9.

HRMS-ESI (m/z): calcd for [$C_{19}H_{20}O_8$, M+H]$^+$: 377.1237, Found: 377.1236.

Synthesis of 6. A mixture of **5** (1.88 g, 5 mmol) and LiOH (0.31 g, 13 mmol) in DMF (180 mL) was purged with argon to degas. The mixture was heated up to 80 °C for 30 min and **2** (1.92 g, 5 mmol) was added. The mixture heated up to 90 °C for 5 h. After cooling to room temperature, the mixture was acidified to pH ≈ 1 with dilute HCl (1 M) and then extracted with CH_2Cl_2 (150 mL × 3). The combined organic layer was washed with NaOH (5% wt), water and brine, dried over $MgSO_4$, and concentrated under reduced pressure. The residue was purified by column chromatography on silica gel with binary eluent (1:1, PE-EA v/v; 1:40, MeOH-DCM, v/v) to yield crude product. The crude product was further recrystallization with ethanol to yield **6** as a white solid (0.40 g, 19%).

Melting point: 215-217 ºC.

^1H NMR (400 MHz, $CDCl_3$) δ 7.62 (dd, *J* = 8.3, 2.6 Hz, 2H, Ar-H), 7.51 (d, *J* = 2.6 Hz, 2H, Ar-H), 6.83 (d, *J* = 8.2 Hz, 2H, Ar-H), 4.27 (d, *J* = 4.7 Hz, 8H, $C\underline{H}_2CH_2C\underline{H}_2$), 3.89 – 3.81 (m, 6H, COOMe), 2.35 (d, *J* = 11.1 Hz, 4H, $CH_2C\underline{H}_2CH_2$).

^{13}C NMR (101 MHz, $CDCl_3$) δ 167.0, 153.1, 148.5, 123.6, 122.5, 113.8, 111.6, 68.0, 51.9, 28.7.

HRMS-ESI (m/z): calcd for $[C_{22}H_{24}O_8, M+NH_4]^+$: 434.1856, Found: 434.1801.

Synthesis of H_2L. A mixture of **6** (0.50 g, 1.20 mmol) and anhydrous LiOH (0.48 g, 20 mmol) in H_2O (20 mL), MeOH (5 mL) THF (15 mL) and was heated at 50 °C for 5 h. After cooling to room temperature, the organic solvents were removed in vacuo and the water phase was acidified to pH ≈ 1 with HCl (3 M). The precipitate was filtered and dried under vacuum at 90 °C for 12 h. **H_2L** was obtained as a white solid (0.46 g, 98%).

^1H NMR (400 MHz, DMSO-d_6) δ 12.62 (s, 2H, COOH), 7.53 (dd, *J* = 8.4, 1.9 Hz, 2H, Ar-H), 7.41 (d, *J* = 2.0 Hz, 2H, Ar-H), 6.99 (d, *J* = 8.4 Hz, 2H, Ar-H), 4.20 (dt, *J* = 10.9, 5.0 Hz, 8H, $C\underline{H}_2CH_2C\underline{H}_2$), 2.23 (d, *J* = 13.2 Hz, 4H, $CH_2C\underline{H}_2CH_2$).

^{13}C NMR (101 MHz, DMSO-d_6) δ 167.6, 153.2, 148.5, 123.7, 123.2, 114.0, 112.4, 68.2, 28.6.

HRMS-ESI (m/z): calcd for $[C_{20}H_{20}O_8, M+NH_4]^+$: 406.1543, Found: 406.1499.

Vapor adsorption isotherms for benzene and cyclohexane

Vapor adsorption isotherms for benzene and cyclohexane were recorded at different temperatures, maintained using a temperature-programmed water bath, on a Hiden Isochema IGA-003 system under ultra-high vacuum (10^{-10} bar) using a turbo pumping system. Ultra-pure research grade benzene and cyclohexane (99.999%) were purchased from Sigma-Aldrich. In a typical vapour adsorption experiment, 50 mg of methanol-exchanged NKU-300 was loaded into the IGA system and activated at 473 K under dynamic high vacuum (10^{-10} bar) for 12 h to give fully desolvated NKU-300. The ultra-pure benzene or cyclohexane was loaded into the liquid container for vapour generation and to avoid vapor condensation within the system, adsorption isotherms were recorded at relative pressure of 0–0.95 at each temperature. Kinetic studies were carried out using

IGA-003 and the pressure was set from 0-5 mbar and 5-10 mbar, respectively at different temperatures.

Benzene vapour adsorption kinetic

To investigate the mechanism of the kinetic trap of benzene within NKU-300, kinetic profiles were measured over a temperature range from 298-318 K, at which the material was expected to display similar uptake capacities. In these experiments, samples were exposed to a pressure of benzene in the range of 0-5 mbar and 5-10 mbar, respectively. corresponding to 1.3 and 0.7 benzene per cage, and the amount of benzene adsorbed was monitored as a function of time. As shown in Figs. S43 and S44, the kinetic profiles at 298, 308 and 318 K exhibit exponential behaviour and the data were fitted using an exponential expression used previously to model vapour sorption kinetics in metal-organic frameworks [2]:

$$\frac{Mt}{Me} = A(1 - e^{-kt}) \qquad (1)$$

where M_t and M_e represent the adsorbed amount of benzene at time t and at equilibrium, respectively; *k* is rate constants and A is Arrhenius constants.

The activation energy barrier E_a, which depends on the rate constant *k* of benzene adsorption at temperature T, can be obtained by fitting to the Arrhenius equation (2).

$$E_a = -R(\frac{\partial lnk}{\partial(1/T)})P \qquad (2)$$

where R is the gas constant. Thus, for an adsorption profile which has a rate constant *k* obeying the Arrhenius equation, a plot of ln(*k*) versus T^{-1} gives a straight line, the slope of which can be used to determine E_a.

Calculation of isosteric heats of adsorption

To estimate the differential enthalpies (ΔH_n) for benzene adsorption, all isotherms at different temperatures were fitted to the van’t Hoff isochore:

$$\ln(P) = \frac{\Delta H_n}{RT} - \frac{\Delta S_n}{R} \qquad (3)$$

where *P* is pressure, *T* is the temperature, *R* is the real gas constant. A plot of ln(*P*) versus 1/*T* at constant amount adsorbed allows the differential enthalpy of adsorption and also the isosteric enthalpy of adsorption (Q_{st}) to be determined.

Kinetic separation experiments

Batch kinetics experiments was performed at 298 K. 13 mg of activated NKU-300 was soaked in 20 mL of benzene/cyclohexane mixture (v/v = 1/1000) or 2 mL of equimolar benzene/cyclohexane mixture, the crystals were collected by filtration and dried in air

to remove the surface-physically adsorbed benzene or cyclohexane molecules at different incubation time (1, 5, 10, 20, 40, 60, 80, 100, and 120 min). And then was digested in DCl/DMSO-d_6 for ^{1}H NMR spectroscopic analysis.

Regeneration of NKU-300

300 mg of activated NKU-300 was soaked in 6 mL of equimolar benzene/cyclohexane mixture for 0.5 h at 298 K, the crystals were collected by filtration and dried in air to remove the surface-physically adsorbed benzene or cyclohexane molecules, and part of this was digested in DCl/DMSO-d_6 for the measurement of ^{1}H NMR. The remaining samples regenerated by heated at 100 °C under vacuum for 1 h, and the PXRD was measured to verify the stability of NKU-300. This process was repeated in subsequent experiments.

Luminescence experiments

NKU-300 were grinded into fine powder before use. The samples for luminescence experiments were dispersed in different solvents by ultrasound for 30 minutes to form a suspension with a concentration of 0.3 mg mL^{-1}. Every 3 mL of this suspension was transferred to a cuvette. The luminescence spectra and luminescence lifetimes of the suspension were measured at room temperature.

Crystal structure determination

The single crystal of H_2L was recrystallized from the mixed solvent of DMF and H_2O, the SCXRD data for H_2L was collected on a Rigaku XtaLAB Mini II X-ray diffractometer equipped with graphite-monochromated Mo-Kα radiation (λ = 0.71073 Å) at 293 K. The crystal of benzene@NKU-300_100K was prepared by soaking in pure benzene. The crystal of benzene@NKU-300_100K-1 was prepared by soaking in benzene/cyclohexane = 1/100. The SCXRD data for NKU-300, benzene@NKU-300_100K, and benzene@NKU-300_100K-1 were obtained on a Rigaku XtaLAB Synergy-R X-ray diffractometer with graphite-monochromated Cu-Kα radiation (λ = 1.54056 Å) at 100 K. All absorption corrections were performed using the multi-scan program. The structures were solved with the SHELXT [3] using Intrinsic Phasing and refined with the full matrix least-squares technique based on F^2 using SHELXL [4]. All non-hydrogen atoms were refined anisotropically, while hydrogen atoms were positioned geometrically and refined as riding.

In situ synchrotron single crystal X-ray diffraction data of benzene-loaded NKU-300 were collected at different temperatures using synchrotron radiation at Beamline I19 of Diamond Light Source, equipped with three-circle goniometer and Rigaku Saturn 724+

CCD detector (λ = 0.4859 Å, double crystal monochromator with Si 111 cryo-cooled crystals). The data of benzene-saturated NKU-300 were collected at 180 K to determine the initial positions of adsorbed-benzene. Subsequently, the temperature was gradually increased to 423 K with or without vacuum on to determine the primary binding domain of adsorbed-benzene (the detailed condition is summarized in Table S7). The dosing of Ar during the removal of benzene is for better heat exchange. The unit cell parameters are listed in Table S7 and the refinement statistics are given in Table S8. In addition, the occupancies of the benzene molecules at sites a to d are summarized in Table S9 and a summary of crystallography data was listed in Tables S10−S19, respectively.

Quantum chemical calculations

Density functional theory (DFT) calculations were carried out to explore the adsorption mechanisms. Gaussian 16 Rev. C.01 was used for all electronic structure calculations [5]. The PBE0 hybrid DFT method was employed, with a 52-electron large core pseudopotential on Eu with MWB52 basis, and the 6-31G* basis set for all other atoms. The crystal structure of benzene@NKU-300 is directly used for the single point calculation without optimization because the framework of NKU-300 is rigid. Since the structural change of NKU-300 before and after the adsorption of benzene is negligible, the structure of NKU-300 and benzene are also extracted from the crystal structure of benzene@NKU-300 without optimization. Electrostatic potential analysis was calculated using Multiwfn 3.8 [6]. Electrostatic potential colored molecular van der Waals surface diagrams were rendered with the program visual molecular dynamics (VMD) [7].

The adsorption energy ΔE_{ads} between NKU-300 and benzene can be calculated using the following equation:

$$\Delta E_{\text{ads}} = E_{\text{benzene@NKU-300}} - E_{\text{NKU-300}} - E_{\text{benzene}} \qquad (4)$$

In Eq. (4), $E_{\text{benzene@NKU-300}}$ and $E_{\text{NKU-300}}$ represent the total energies of NKU-300 with and without benzene, respectively. E_{benzene} is the energy of the benzene.

Molecular dynamics calculations

Molecular dynamics (MD) simulations were carried out in the xTB program [8] with the force-field GFN-FF [9] to investigate the mobility of the benzene and cyclohexane in a single channel of NKU-300. A 32-layer channel was extracted from the crystal structures of benzene@NKU-300 and the optimized structure of cyclohexane@NKU-300, respectively, and nitrate ions are used to balance the charge. We placed a single benzene or cyclohexane in the middle of the channel and the initial locations were based

on the single crystal X-ray diffraction data or the optimized structure, which are the most energetically favorable locations (site a for benzene@NKU-300 and site d' for cyclohexane@NKU-300). We also placed two or three benzene in the middle of the channel based on two benzene-saturated adsorption configurations (type I and II). The structure of the channel is constrained with force constant of 1.0. The structure optimization was performed using approximate normal coordinate rational function optimizer (ANCopt) and a loose optimization level was chosen. The optimized structure was then used for the MD simulation. Four times mass of hydrogen atoms was chosen. First the structure was equilibrated for 4 ps and a 100 ps simulation using the NVT ensemble with the Berendsen thermostat at 300 K was followed to observe the guest mobility. The interval for trajectory printout is 20 fs and the time step are 0.5 fs. The trajectories were rendered with the program VMD.

Table S1. Comparison of the selected materials for liquid phase separation of benzene/cyclohexane (v/v = 1/1).

Materials	Benzene/ cyclohexane selectivity	Contact time / hours	Separate mode	Ref
Hybrid[3]arene	39	7	vapor diffusion/298K	[10]
L^H-$Au_{10}S_4$-Cl (L^H = 4,5-bis(diphenylphosphanyl)-2*H*-1,2,3-triazole)	24	12	soaking/298K	[11]
Carborane Metallacage	66	4	vapor diffusion/298K	[12]
MAF-stu-13	138	18	stirred/soaking/298K	[13]
MFM-300(Sc)	166	24	soaking/298K	[14]
MFM-300(Cr)	103	24	soaking/298K	[14]
MFM-300(In)	92	24	soaking/298K	[14]
UIO-66	3	24	soaking/298K	[14]
$[Zn_4(EgO_2)_2(tdc)_2(dabco)]$ (ethylene glycol = EgO_2, tdc = thiophene-2,5-dicarboxylate, dabco = 1,4-diazo[2.2.2.]bicyclooctane)	92	120	soaking/298K	[15]
BNF-2	124	12	soaking/353K	[16]
ZnL (H_2L = (*R*,*R*)-(–)-*N*,*N*’-bis(3-*tert-butyl*-5-(4-ethynylpyridyl)salicylidene)-1,2-diaminocyclohexane)	20	48	soaking/353K	[17]
$[Li_2Zn_2(NO_2\text{-}bdc)_3(bpy)]$ (H_2bdc = 1,4-benzenedicarboxylic acid, bpy = 4,4’-bipyridyl)	16	24	soaking/298K	[18]
HKUST-1	3	72	soaking/298K	This work
ZIF-8	10	72	soaking/298K	This work
MOF-74	4	72	soaking/298K	This work

ZSM-5	2	24	soaking/298K	This work
Zeolite 13X	55	48	soaking/298K	This work
Activated carbon	2	48	soaking/298K	This work
NKU-300	221	0.33	soaking/298K	This work

Table S2. Comparison of the selected materials for liquid phase separation of benzene/cyclohexane (v/v = 1/100).

Materials	Benzene/ cyclohexane selectivity	Contact time / hour	Separate mode	Ref
UIO-66	28	48	soaking/298K	This work
HKUST-1	96	72	soaking/298K	This work
ZIF-8	282	72	soaking/298K	This work
MOF-74	84	72	soaking/298K	This work
ZSM-5	86	24	soaking/298K	This work
Zeolite 13X	226	48	soaking/298K	This work
Activated carbon	20	48	soaking/298K	This work
NKU-300	2120	0.33	soaking/298K	This work

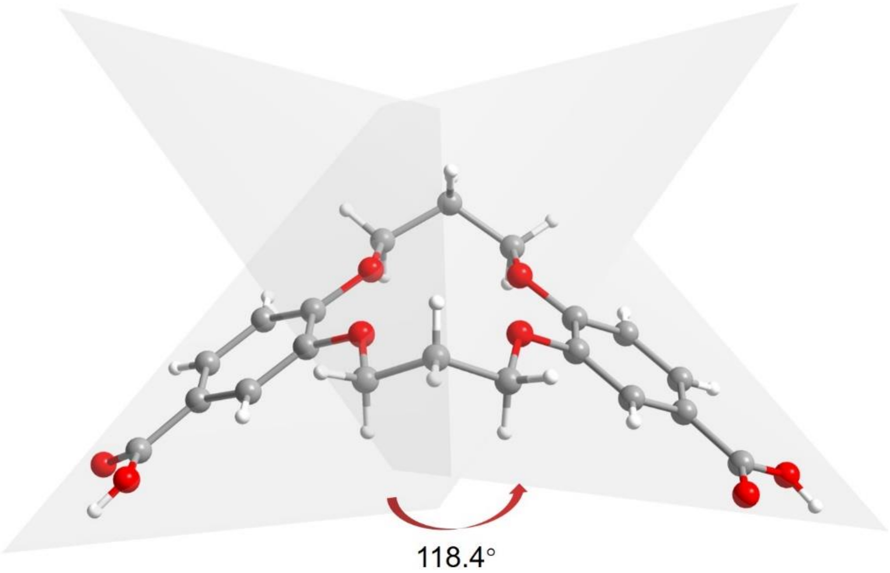

Figure S1. View of the X-ray single-crystal structure of **H_2L**. The **H_2L** molecule exhibits a significant bending in the core with the dihedral angle between the adjacent phenyl rings of 61.6°, which has an approximate length of 13.1 Å and a width of 6.2 Å. A summary of crystallography data was listed in Table S3.

Table S3. Crystal data and structure refinement for **H_2L**. Standard deviations are expressed in brackets in terms of the least significant digit(s).

Complexes	**H_2L**
Formula	$C_{20}H_{20}O_8$
Formula weight	388.36
Temperature (K)	293
Wavelength (Å)	0.71073
Crystal system	Triclinic
Space group	*P*-1
a (Å)	4.8371(5)
b (Å)	11.2489(10)
c (Å)	16.9238(13)
α (°)	90.617(7)
β (°)	91.892(7)
γ (°)	102.100(8)
V (Å^3)	899.76(14)
F(000)	408.0
Z	2
ρ_{calc} (g cm^{-3})	1.433
μ (mm^{-1})	0.112
2θ range (°)	4.382–50.700
Ref. meas. / indep.	13685/3276
R_{int}	0.1046
$^aR_1$ / $^bwR_2$ [$I \geq 2\sigma(I)$]	0.0665/0.0984
$^aR_1$ / $^bwR_2$ (all data)	0.1998/0.1284
GOF on F^2	0.930
Largest diff. peak/hole (e.Å^{-3})	0.23/-0.24
CCDC	2320896

$^a R_1 = \Sigma||F_o| - |F_c||/\Sigma|F_o|$. $^b wR_2 = [\Sigma w(F_o^2 - F_c^2)^2/\Sigma w(F_o^2)^2]^{1/2}$.

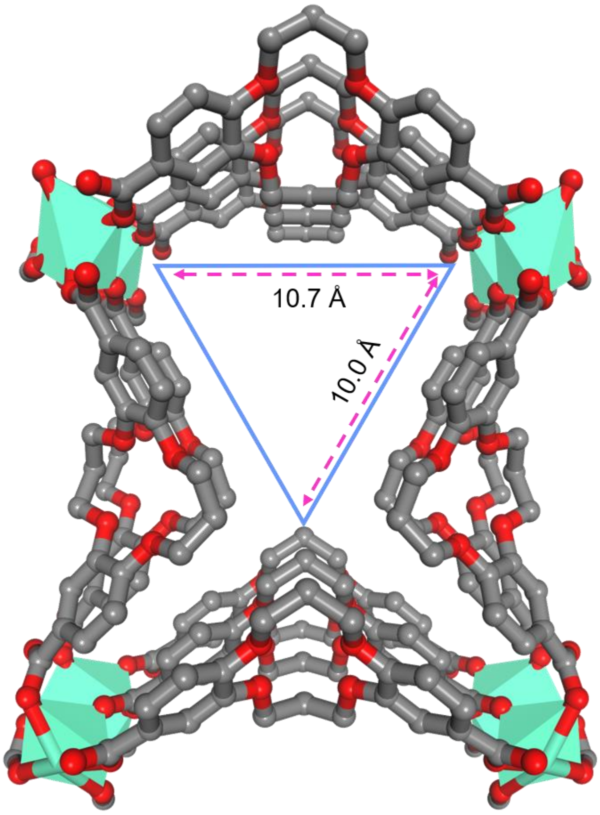

Figure S2. View of the triangular channel of NKU-300. A summary of crystallography data was listed in Table S4.

Table S4. Crystal data and structure refinement for NKU-300_100K. Standard deviations are expressed in brackets in terms of the least significant digit(s).

Complexes	NKU-300_100K
Formula	$C_{30}H_{27}EuO_{12}$
Formula weight	731.47
Temperature (K)	100
Wavelength (Å)	0.71073
Crystal system	Monoclinic
Space group	$P2_1/m$
a (Å)	4.8601(2)
b (Å)	37.4324(11)
c (Å)	17.2628(5)
α (°)	90.00
β (°)	96.308(3)
γ (°)	90.00
V (Å^3)	3121.52(18)
F(000)	1464.0
Z	4
ρ_{calc} (g cm^{-3})	1.556
μ (mm^{-1})	2.070
2θ range (°)	4.036–53.000
Ref. meas. / indep.	26220/6541
R_{int}	0.0510
$^aR_1$ / $^bwR_2$ [$I \geq 2\sigma(I)$]	0.0268/0.0537
$^aR_1$ / $^bwR_2$ (all data)	0.0376/0.0558
GOF on F^2	1.029
Largest diff. peak/hole (e.Å^{-3})	0.51/-0.60
CCDC	2320897

$^a R_1 = \Sigma||F_o| - |F_c||/\Sigma|F_o|$. $^b wR_2 = [\Sigma w(F_o^2 - F_c^2)^2/\Sigma w(F_o^2)^2]^{1/2}$.

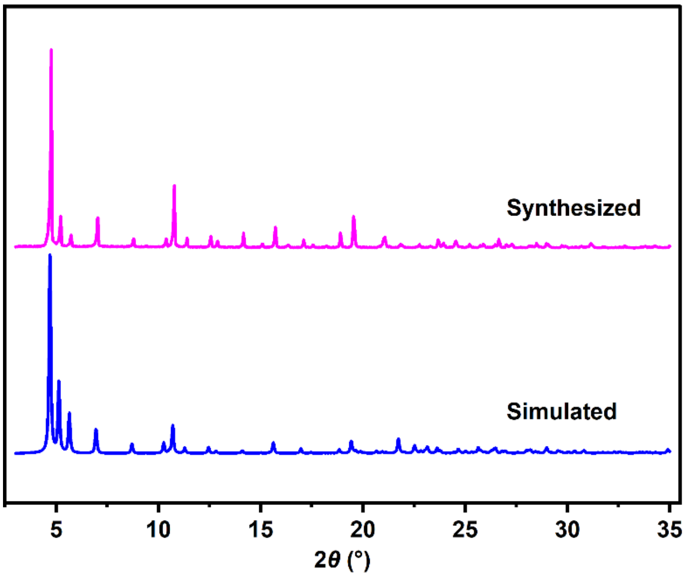

Figure S3. Comparison of the simulated and experimental PXRD patterns of NKU-300.

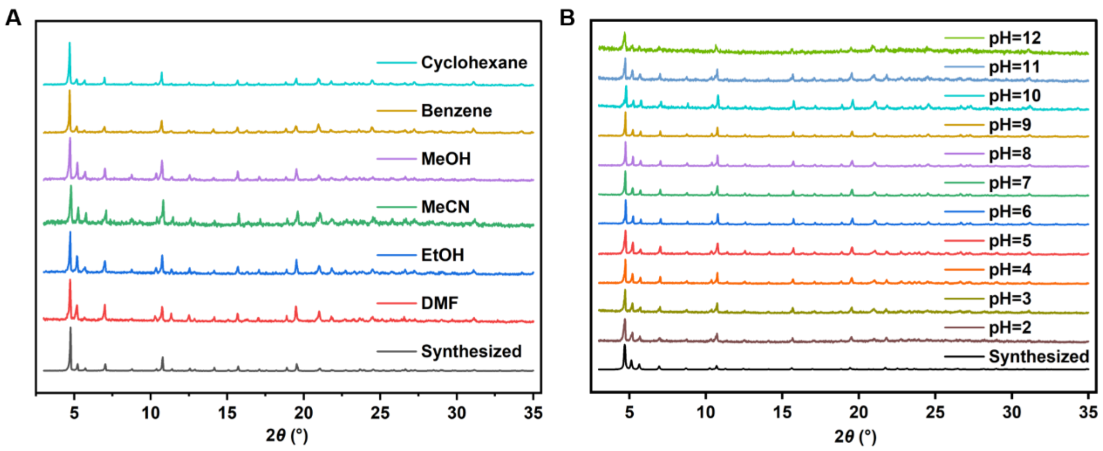

Figure S4. PXRD patterns of NKU-300 after immersing in common organic solvents (**A**) and aqueous solutions with pH values ranging from 2 to 12 (**B**) for 1 day.

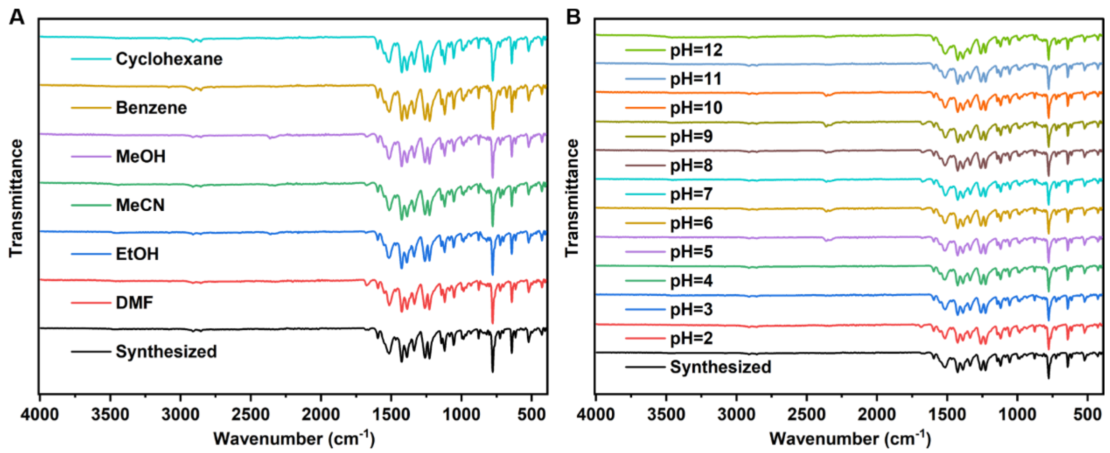

Figure S5. The IR spectra of NKU-300 before and after immersing in common organic solvents (**A**) and aqueous solutions with pH values ranging from 2 to 12 for 1 day (**B**).

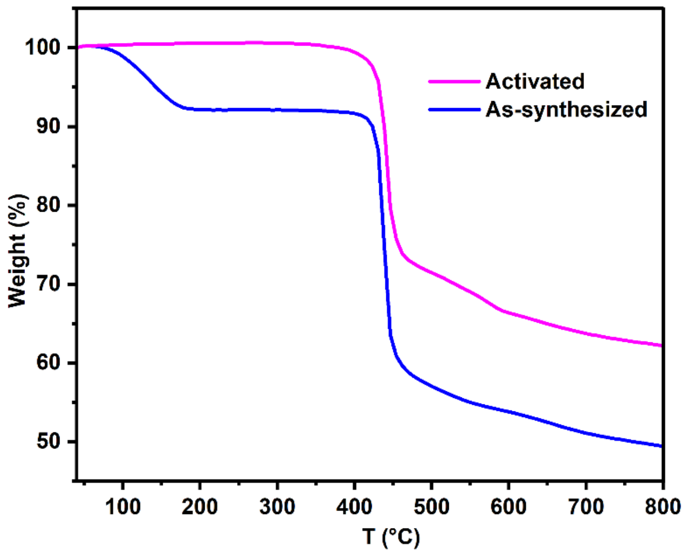

Figure S6. Thermogravimetric analysis curves of NKU-300 before and after activated.

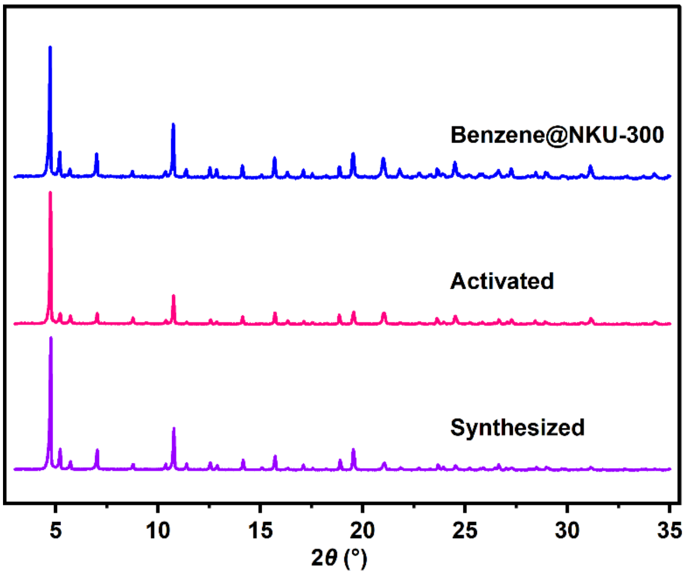

Figure S7. PXRD patterns of as-synthesized, activated and benzene-loaded NKU-300.

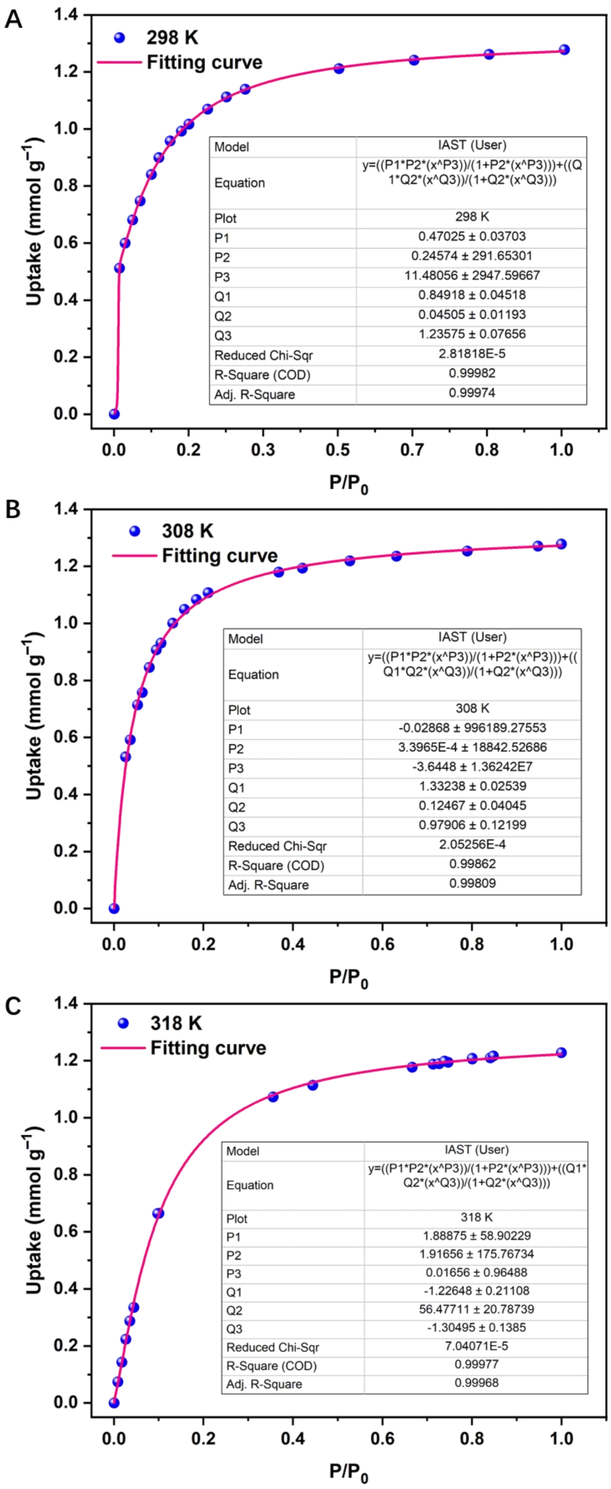

Figure S8. Fitted isotherms of benzene using dual-site fitting model at 298K (**A**), 308 K (**B**) and 318 K (**C**).

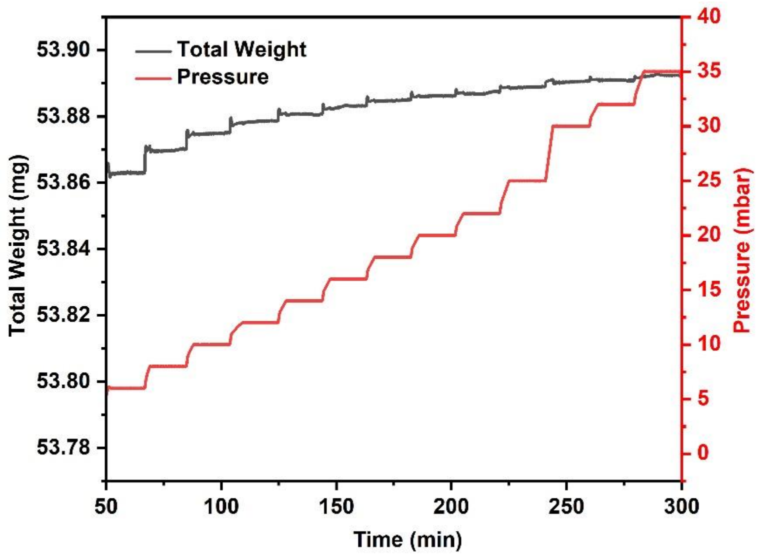

Figure S9. The adsorption kinetics curves of cyclohexane uptake by NKU-300 at 298 K.

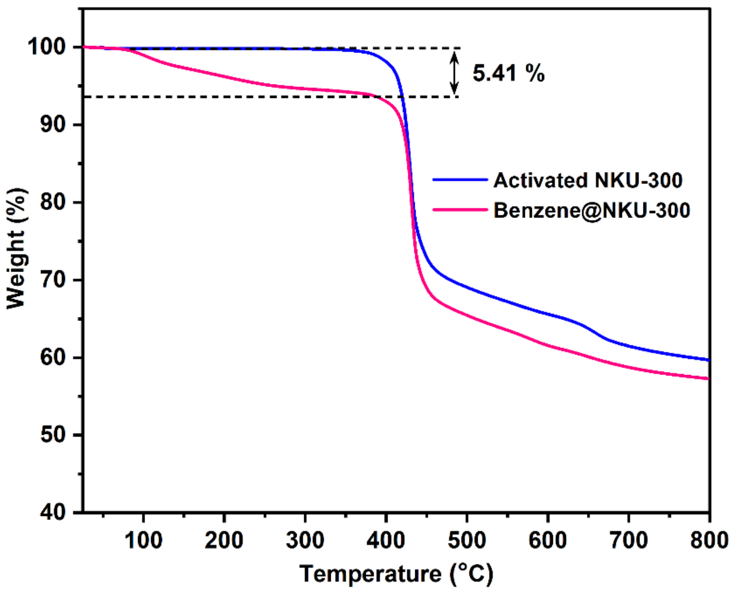

Figure S10. Thermogravimetric analysis curves of NKU-300 before and after benzene adsorption.

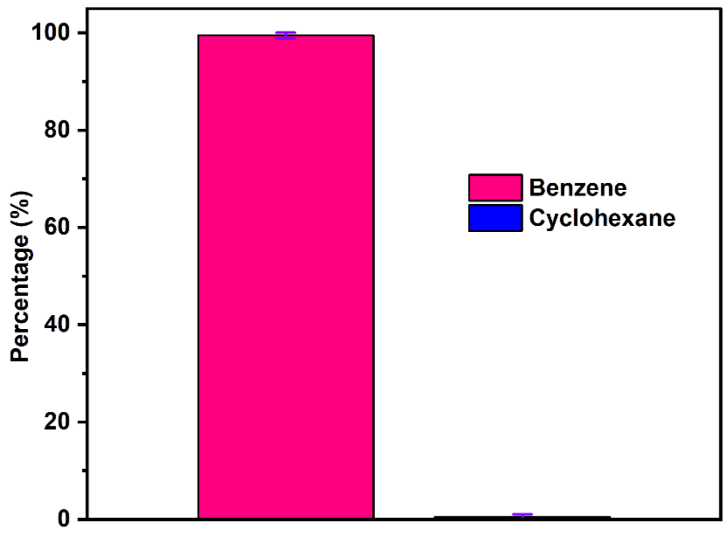

Figure S11. Relative uptakes of benzene and cyclohexane adsorbed by NKU-300 over 0.5 h, determined by ^{1}H NMR spectroscopy.

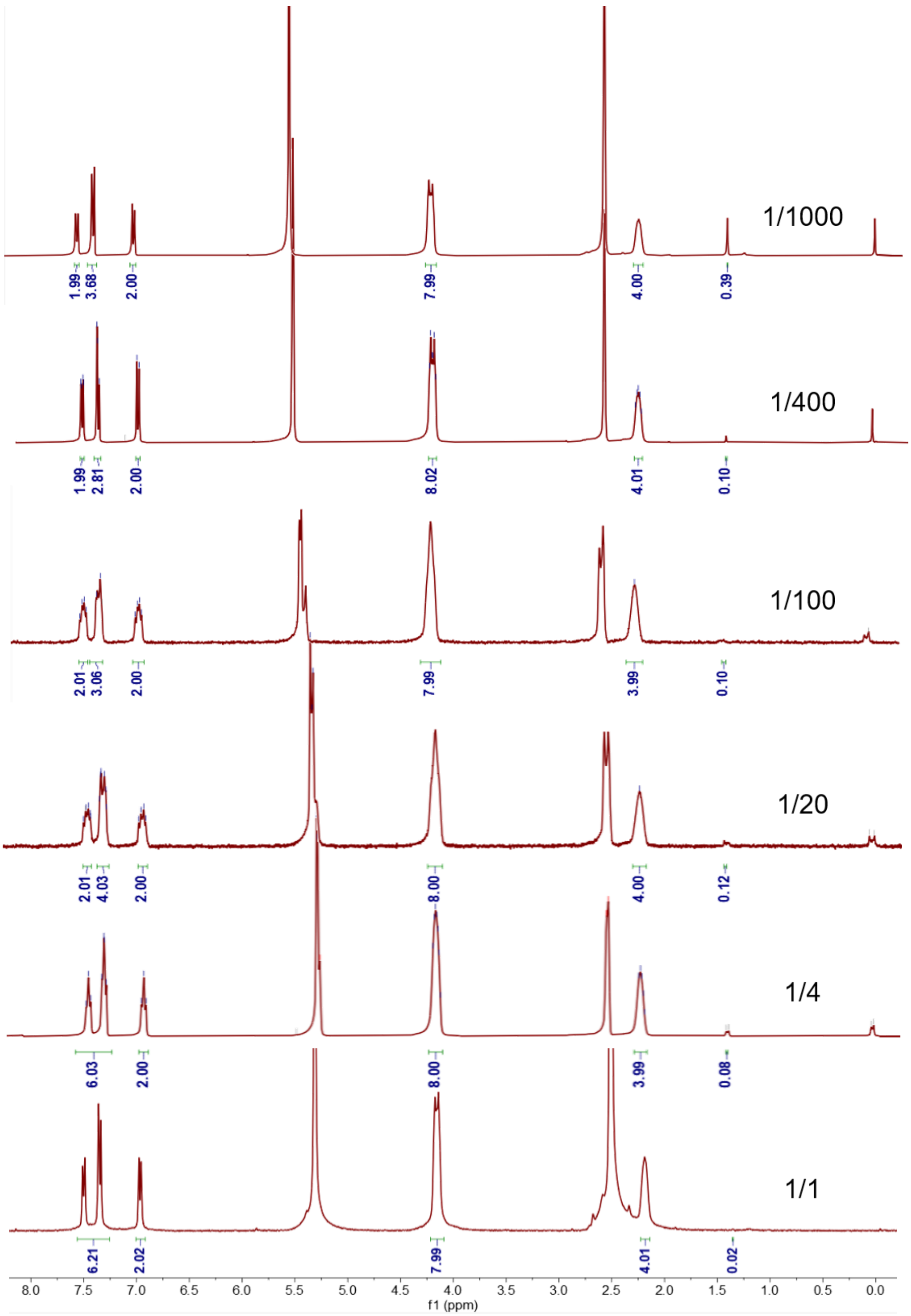

Figure S12. ^{1}H NMR spectra of digested NKU-300 after liquid-phase separation of benzene/cyclohexane with different proportion (v/v = 1/1, 1/4, 1/20, 1/100, 1/400, 1/1000).

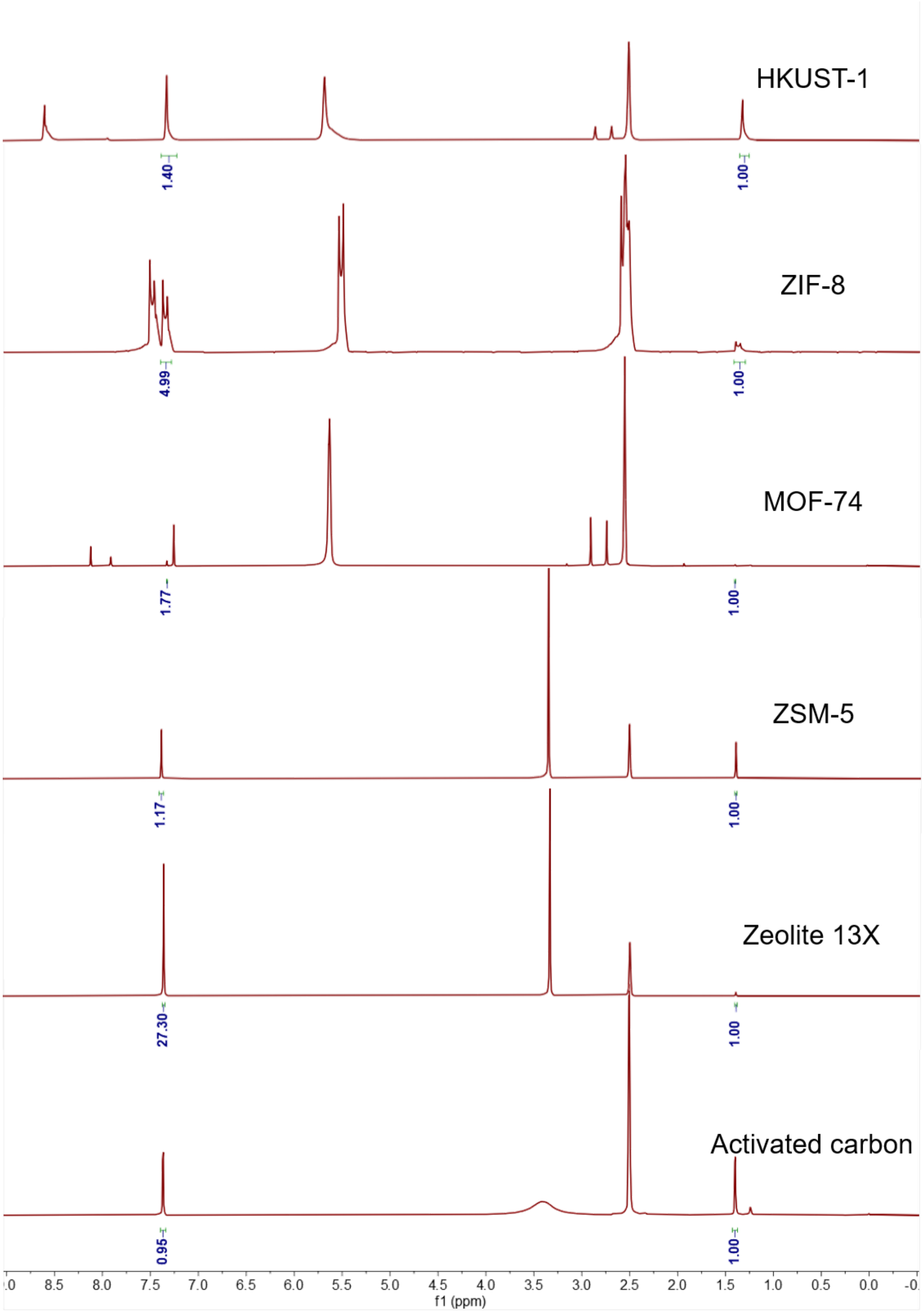

Figure S13. ^{1}H NMR spectra of digested HKUST-1, ZIF-8, MOF-74, ZSM-5, Zeolite 13X, and activated carbon after liquid-phase separation of benzene/cyclohexane (v/v = 1/1).

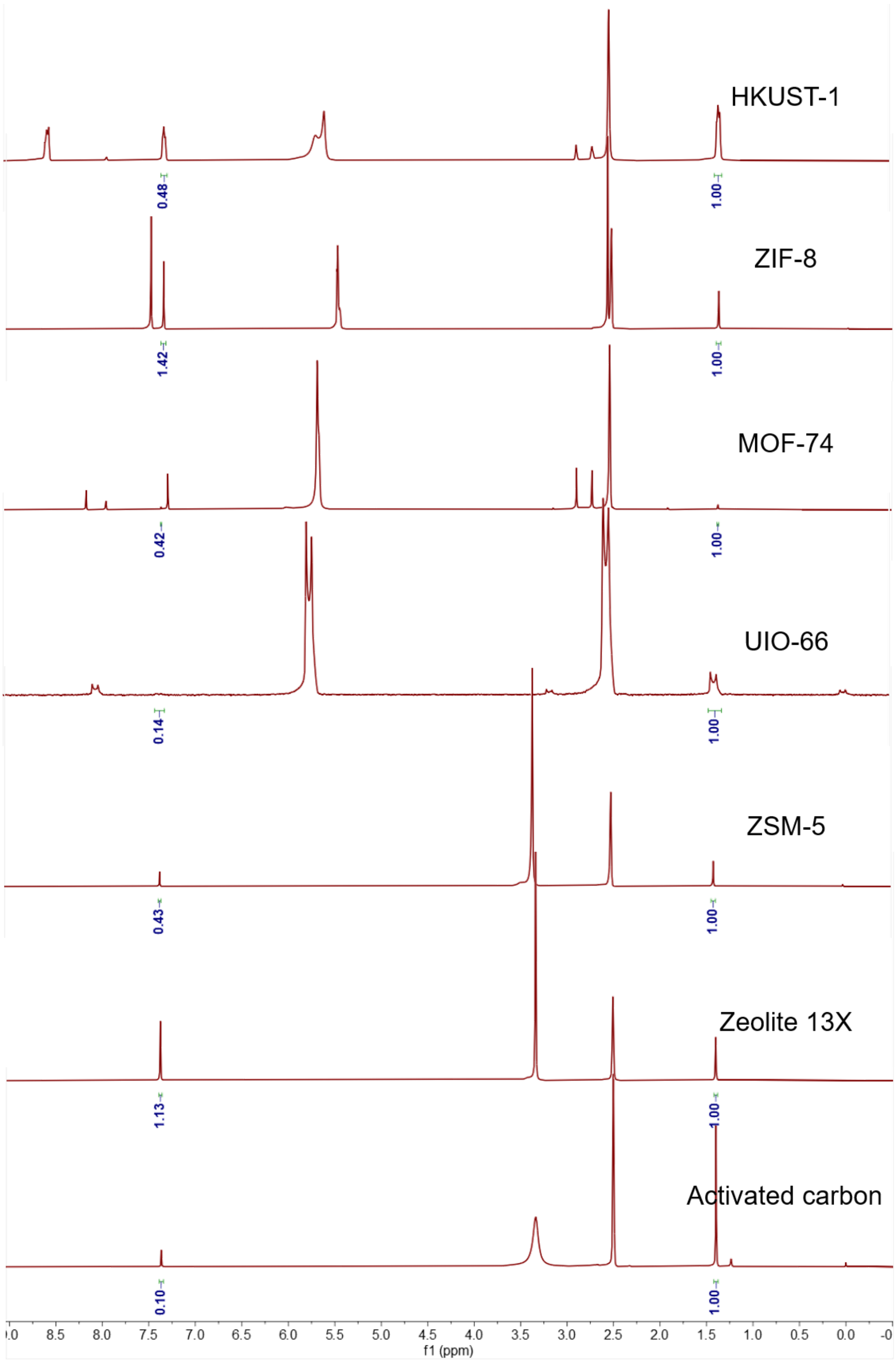

Figure S14. ^{1}H NMR spectra of digested HKUST-1, ZIF-8, MOF-74, UIO-66, ZSM-5, Zeolite 13X, and activated carbon after liquid-phase separation of benzene/cyclohexane (v/v = 1/100).

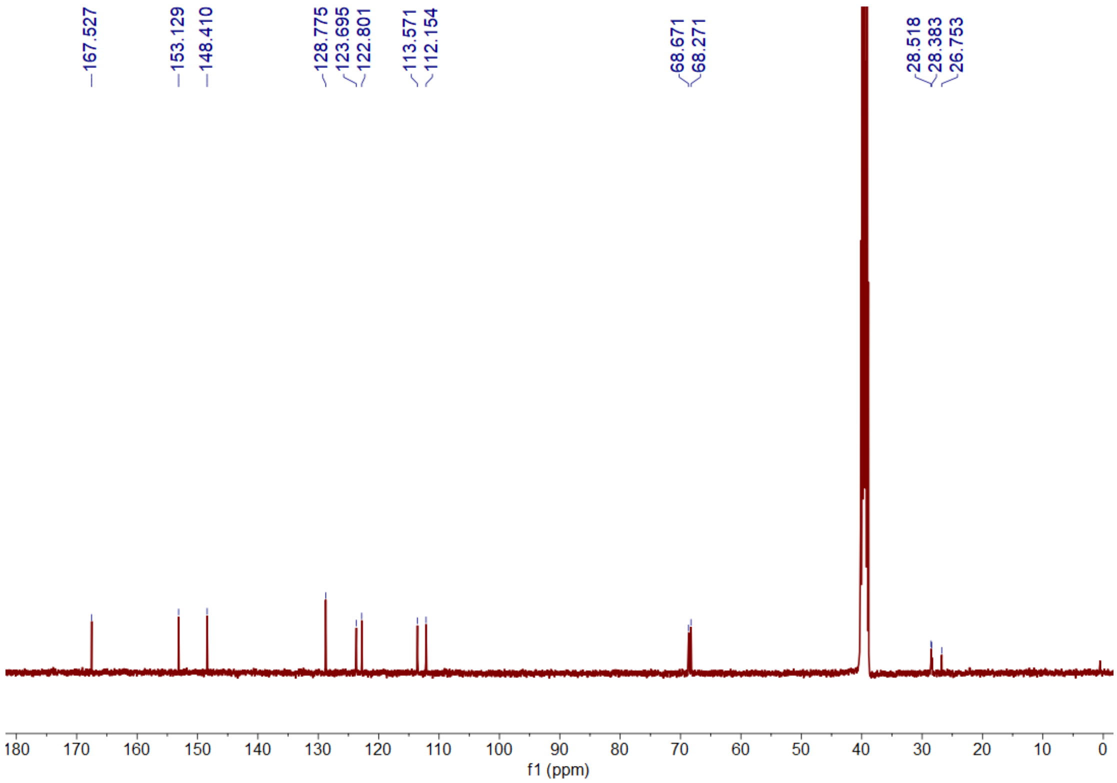

Figure S15. ^{13}C NMR spectra of digested NKU-300 after liquid-phase separation of benzene/cyclohexane mixture (v/v = 1/1000).

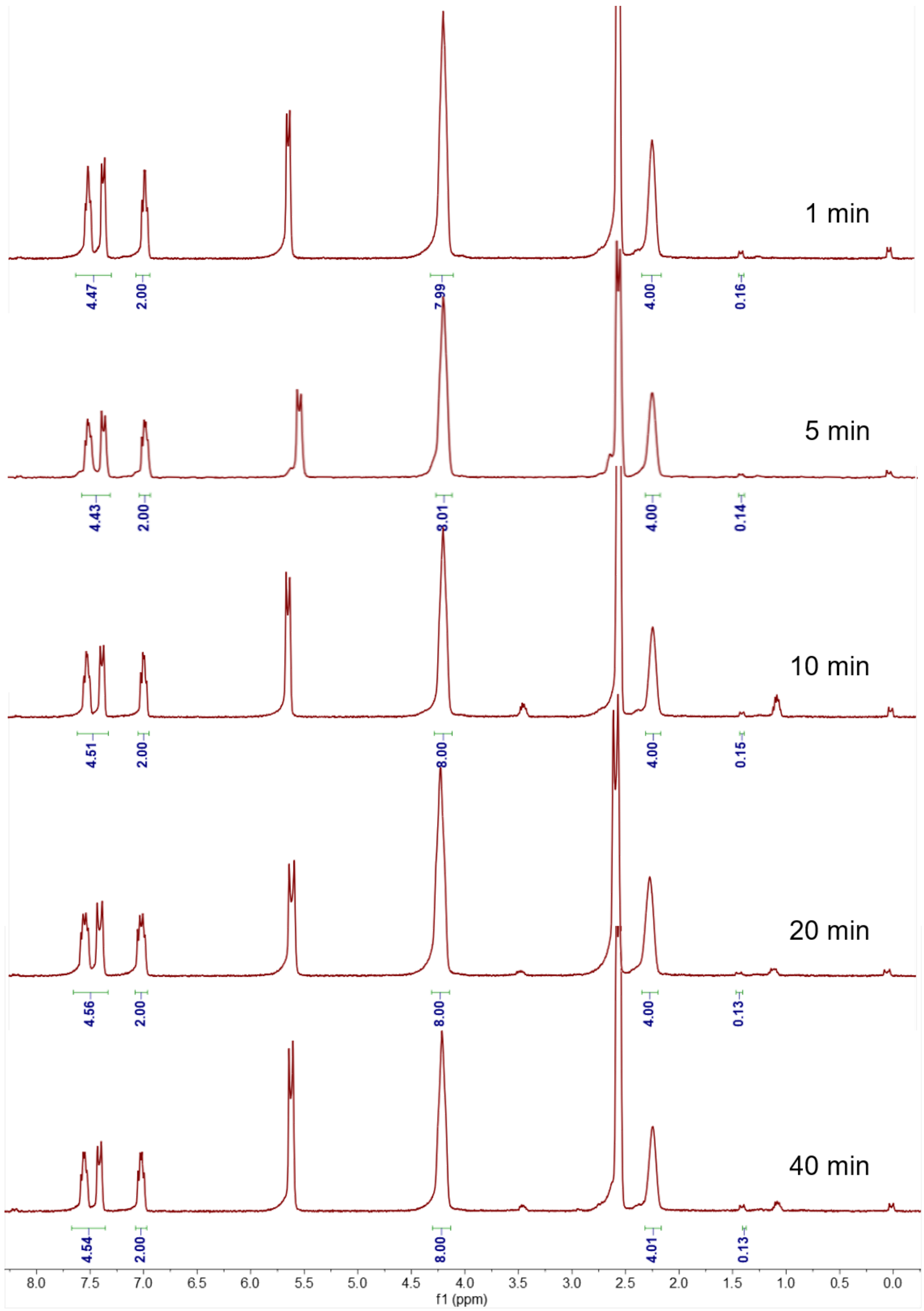

Figure S16. ^{1}H NMR spectra of digested NKU-300 after liquid-phase separation of benzene/cyclohexane mixture (v/v = 1/1000) at different incubation time (1, 5, 10, 20, and 40 min).

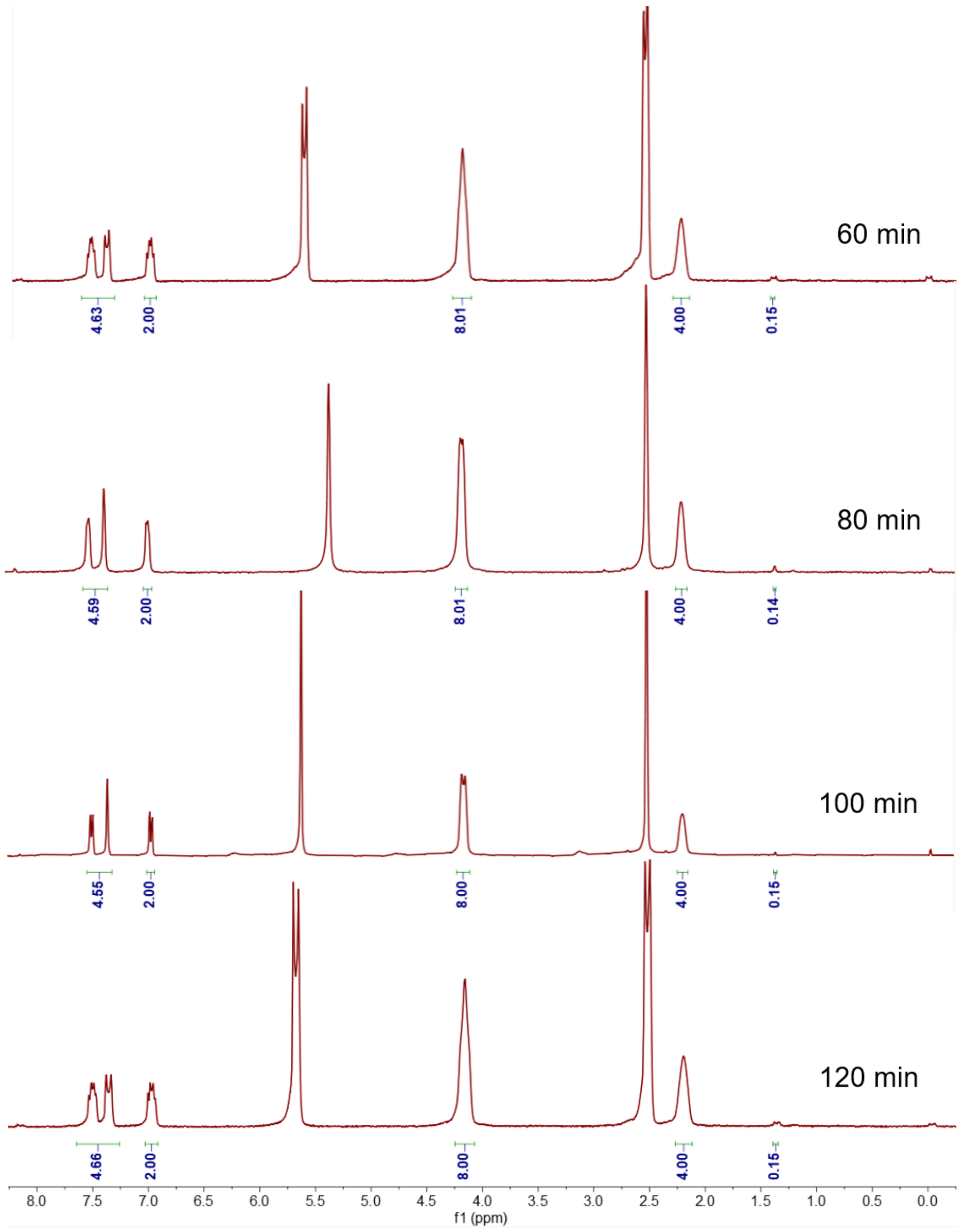

Figure S17. ^{1}H NMR spectra of digested NKU-300 after liquid-phase separation of benzene/cyclohexane mixture (v/v = 1/1000) at different incubation time (60, 80, 100, and 120 min).

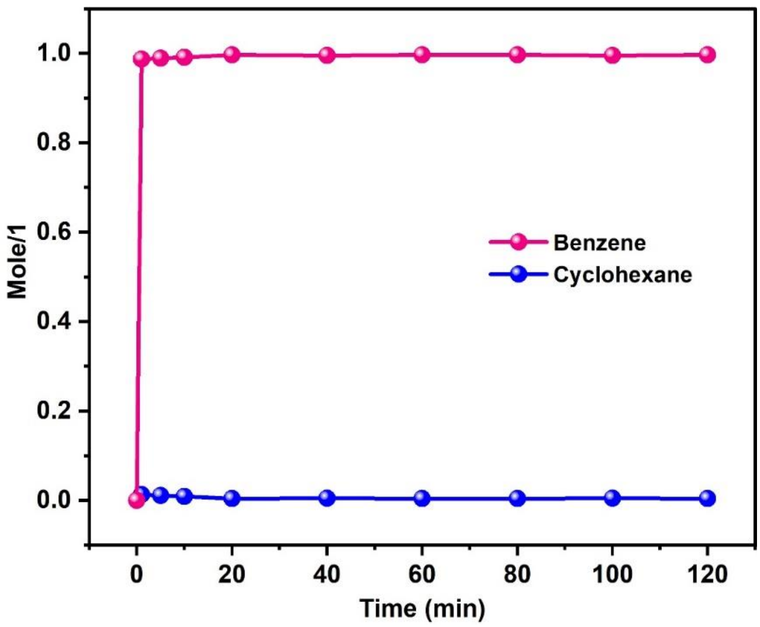

Figure S18. Time-dependent solid-liquid sorption profiles for the equimolar mixture of benzene/cyclohexane in NKU-300. The kinetic separation was also conducted using the equimolar mixture of benzene/cyclohexane. The kinetic plots revealed an exceptional dynamic selectivity of >70 within 1 minute, and at the adsorption equilibrium in 20 minutes, a selectivity of 226 is obtained (Figs. S19−20).

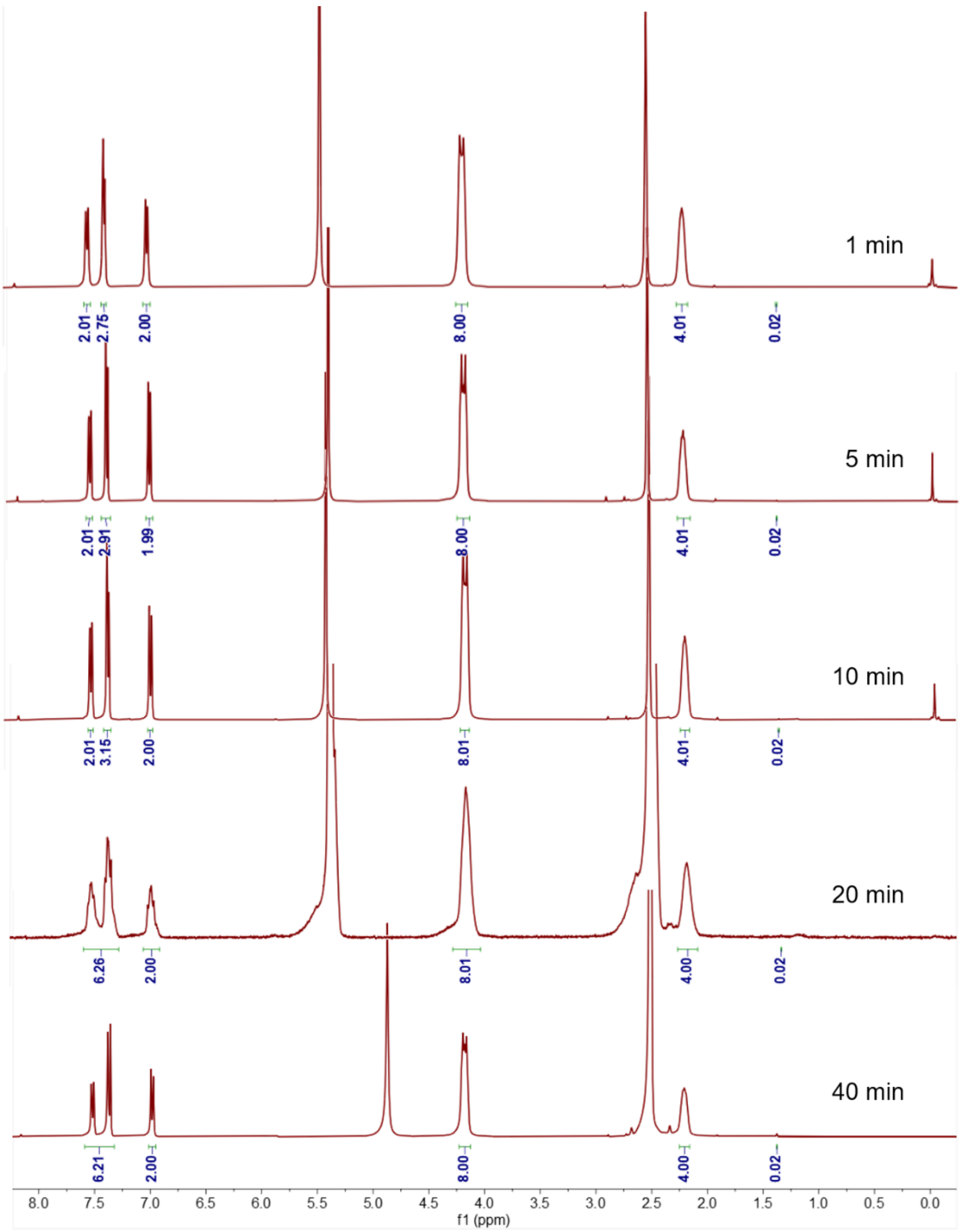

Figure S19. ^1H NMR spectra of digested NKU-300 after liquid-phase separation of equimolar benzene/cyclohexane mixture at different incubation time (1, 5, 10, 20, and 40 min).

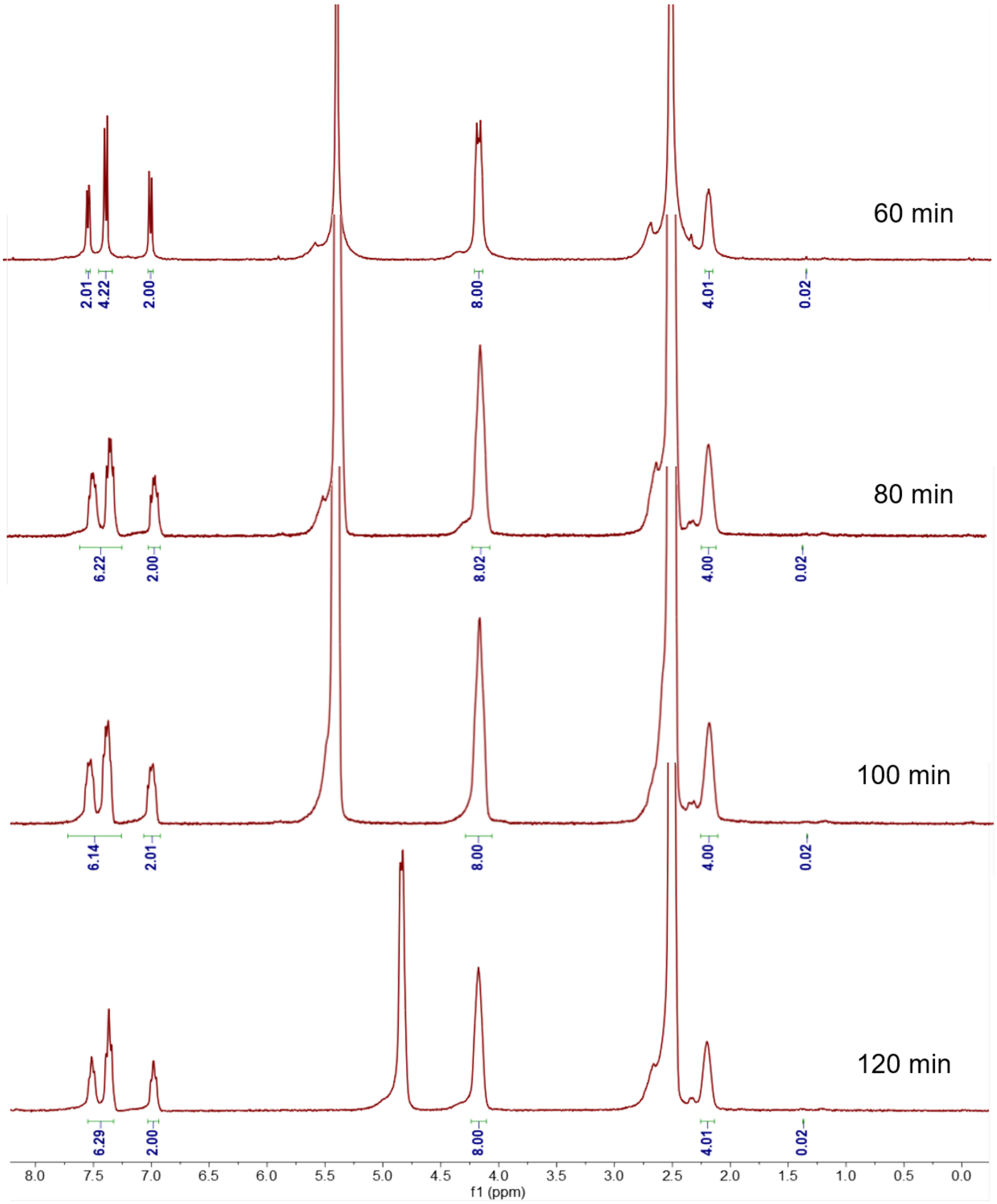

Figure S20. ^{1}H NMR spectra of digested NKU-300 after liquid-phase separation of equimolar benzene/cyclohexane mixture at different incubation time (60, 80, 100, and 120 min).

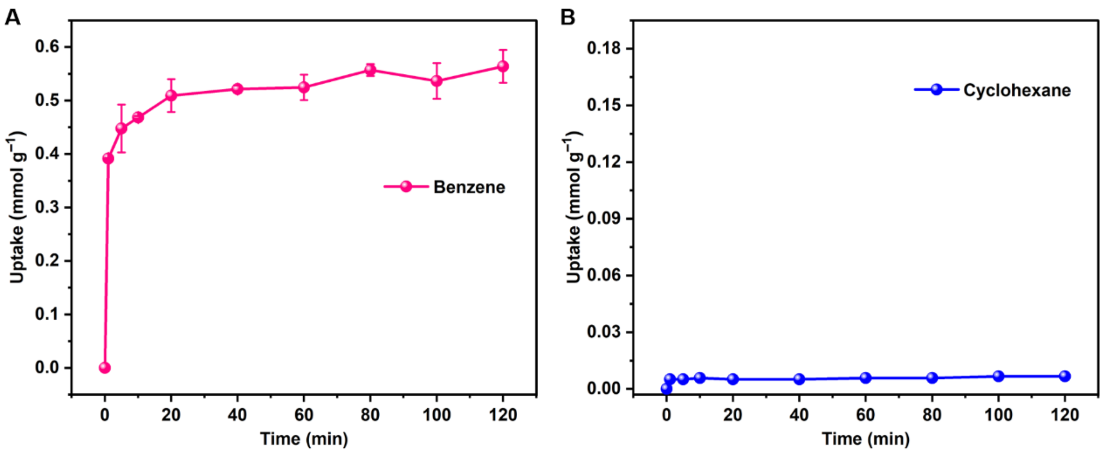

Figure S21. Time-dependent solid-liquid sorption profiles for benzene (**A**) and cyclohexane (**B**) in NKU-300.

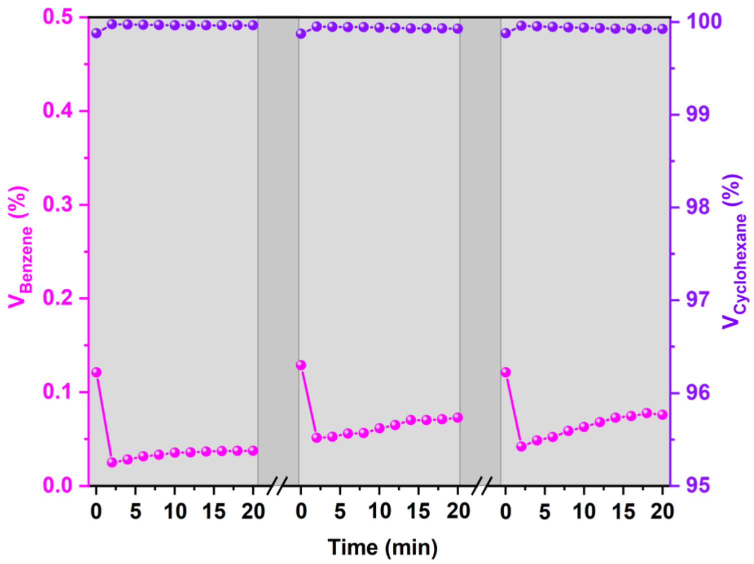

Figure S22. Time-dependent sorption profiles for mixture of liquid benzene/cyclohexane (v/v = 1/1000) by a fixed-bed packed with NKU-300.

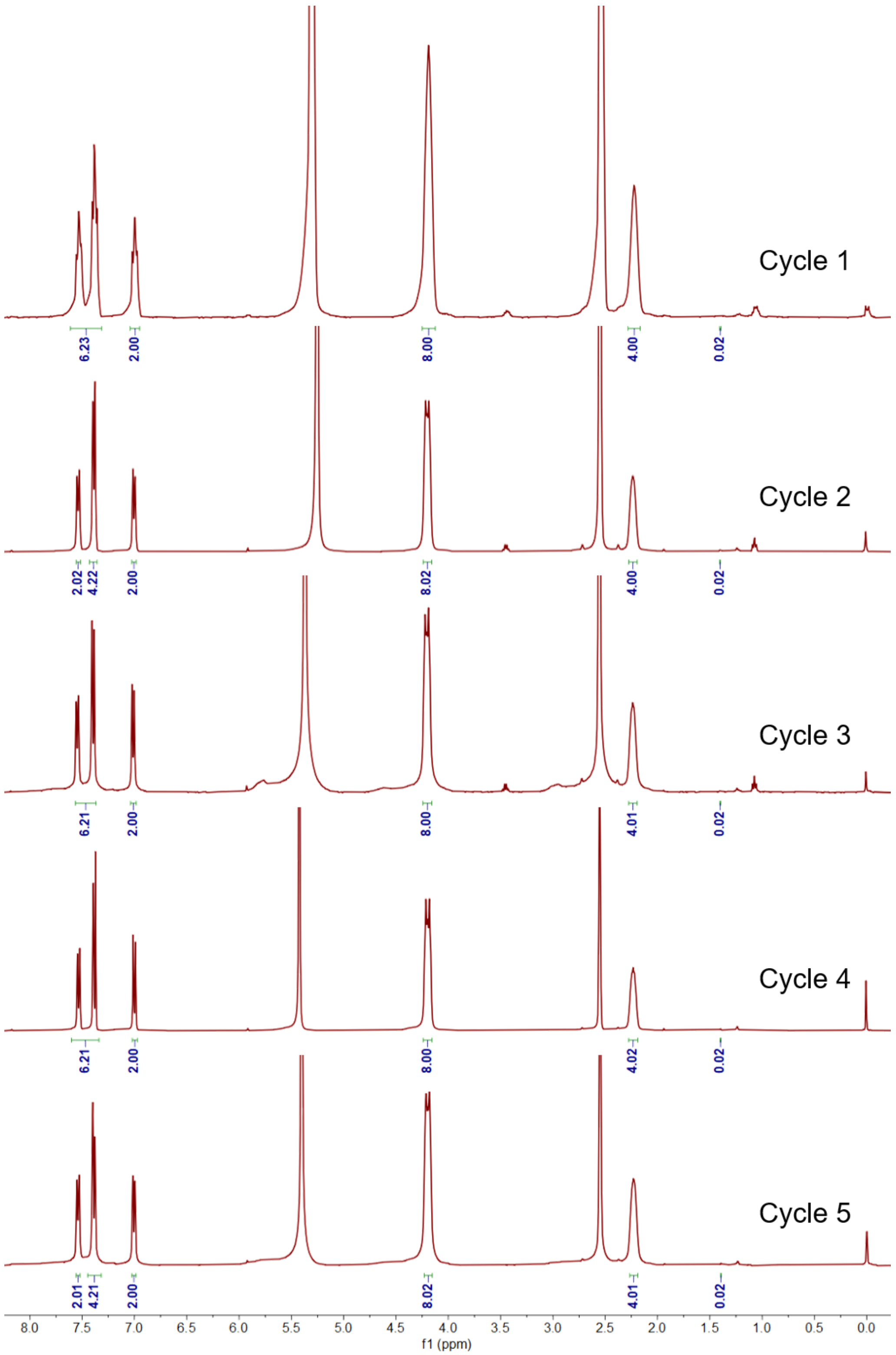

Figure S23. ^{1}H NMR spectra of digested NKU-300 after liquid-phase separation of equimolar benzene/cyclohexane mixture after cyclic separation experiments (cycles 1-5).

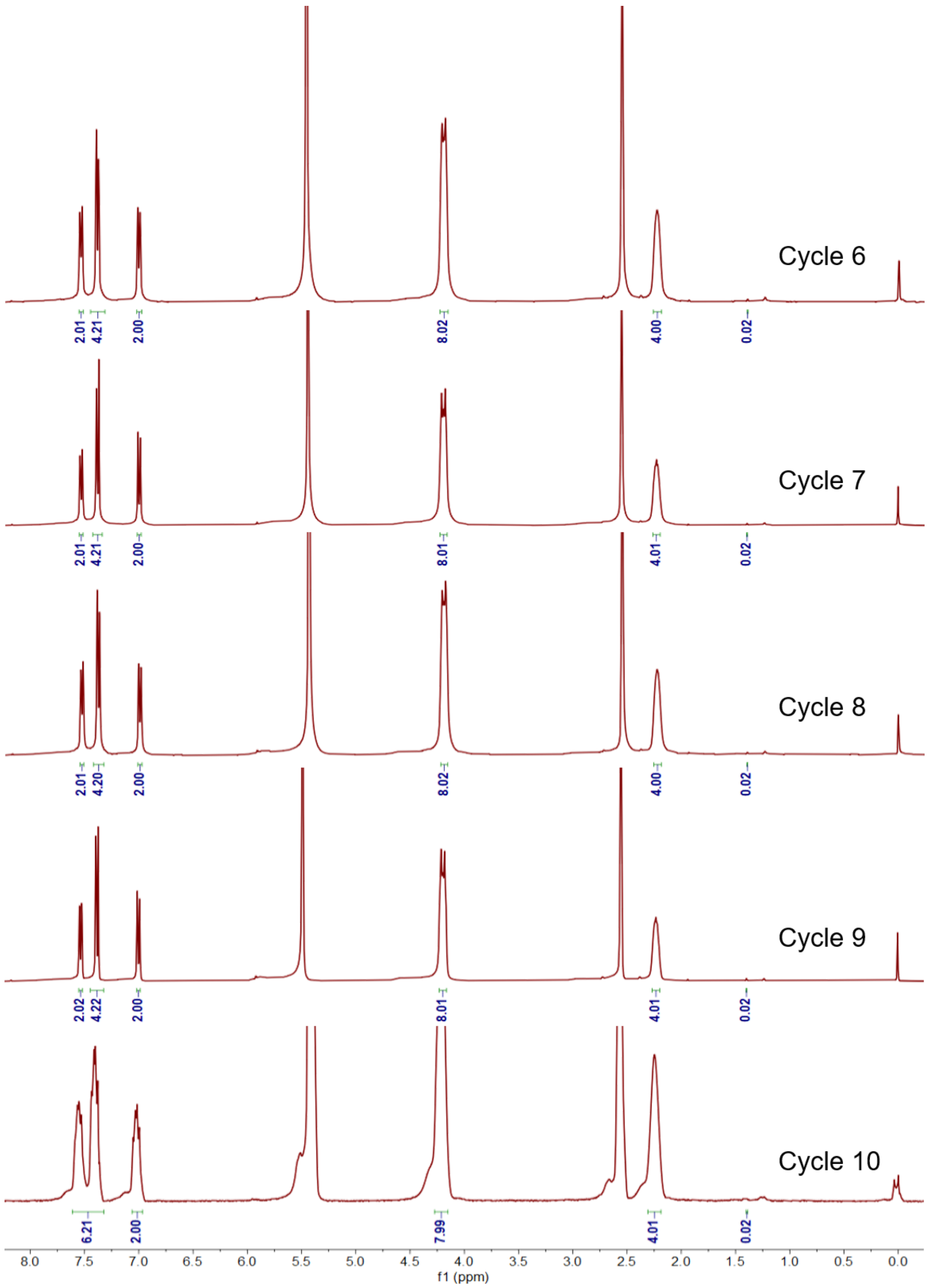

Figure S24. ^{1}H NMR spectra of digested NKU-300 after liquid-phase separation of equimolar benzene/cyclohexane mixture after cyclic separation experiments (cycles 6-10).

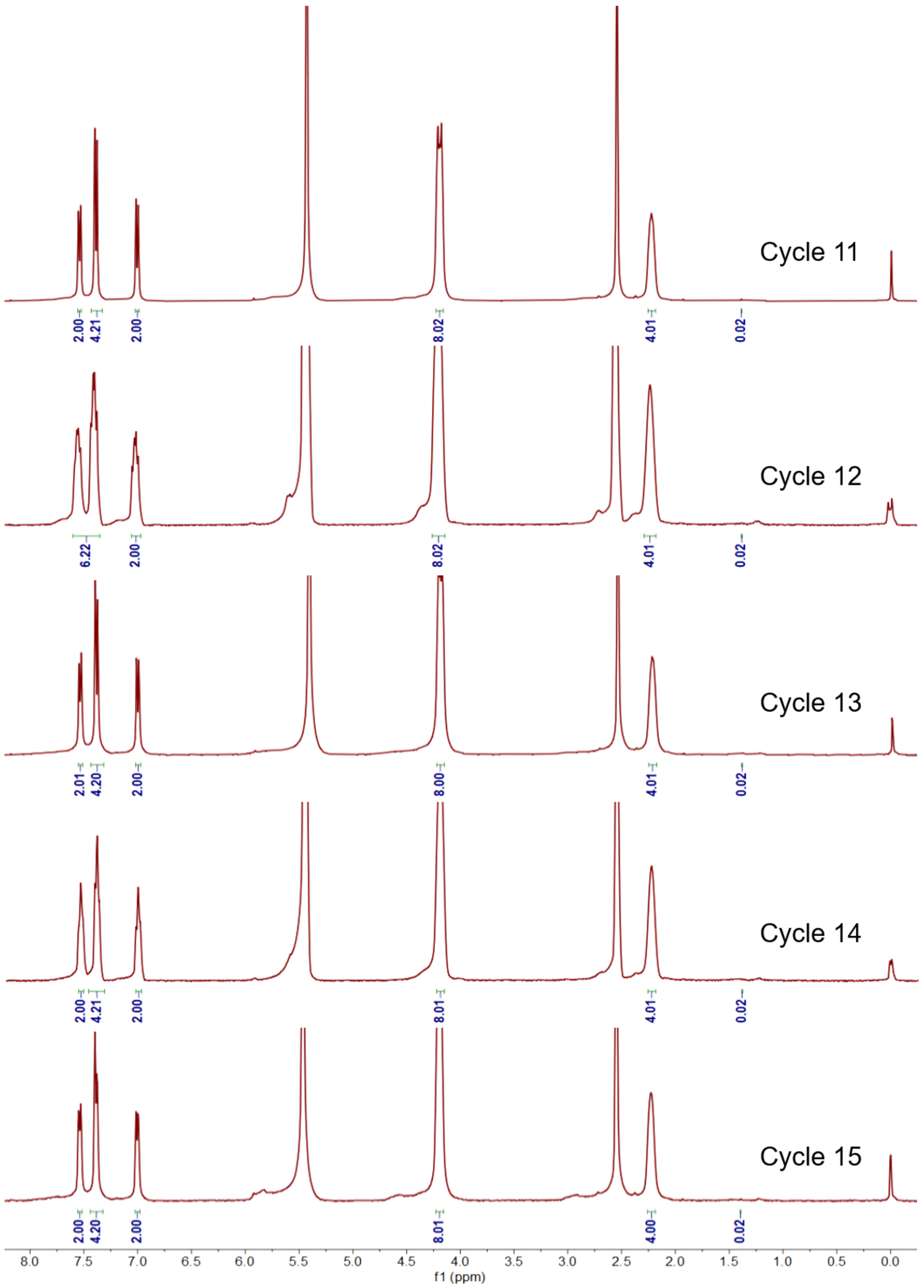

Figure S25. ^{1}H NMR spectra of digested NKU-300 after liquid-phase separation of equimolar benzene/cyclohexane mixture after cyclic separation experiments (cycles 11-15).

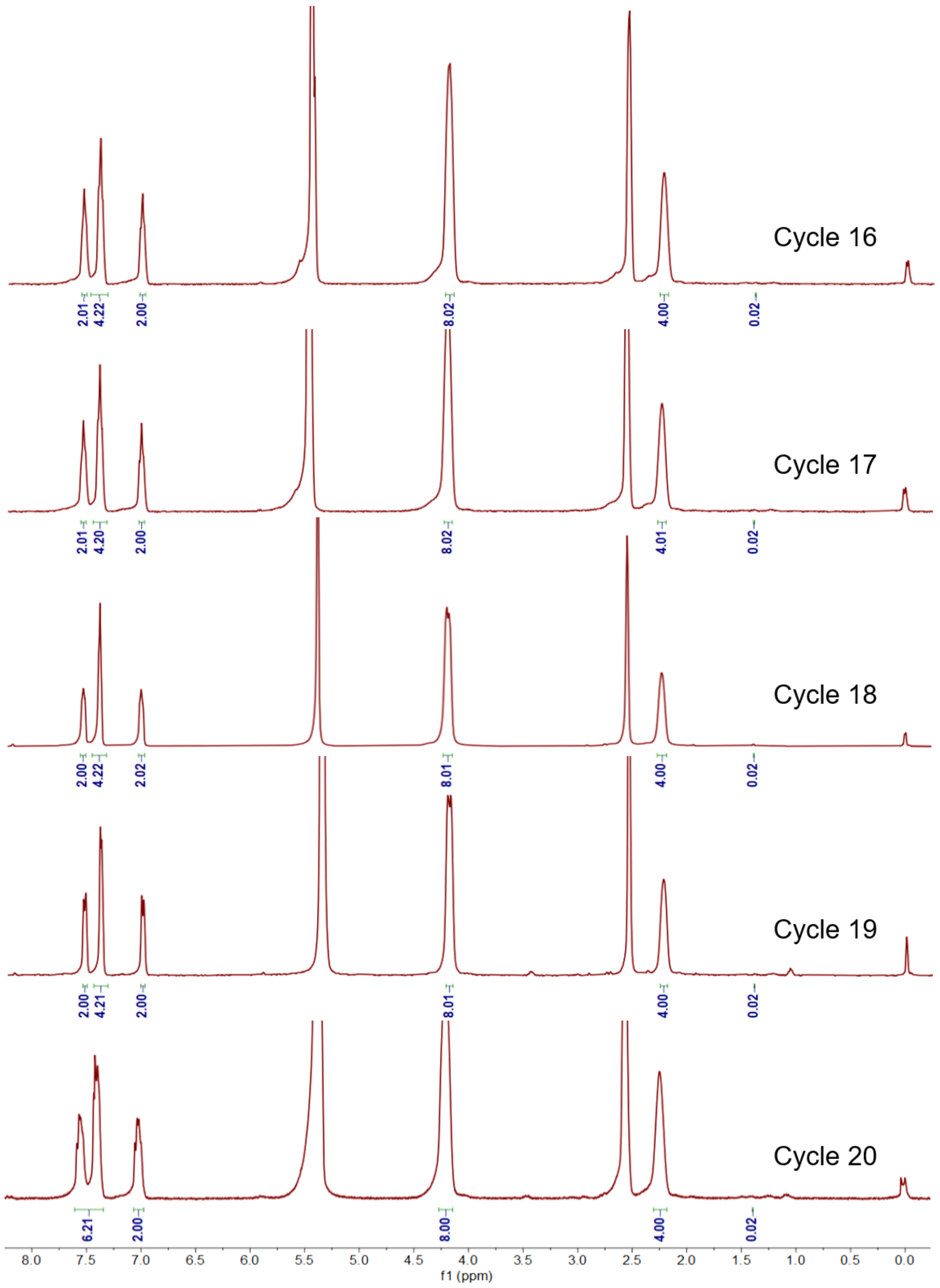

Figure S26. ^{1}H NMR spectra of digested NKU-300 after liquid-phase separation of equimolar benzene/cyclohexane mixture after cyclic separation experiments (cycles 16-20).

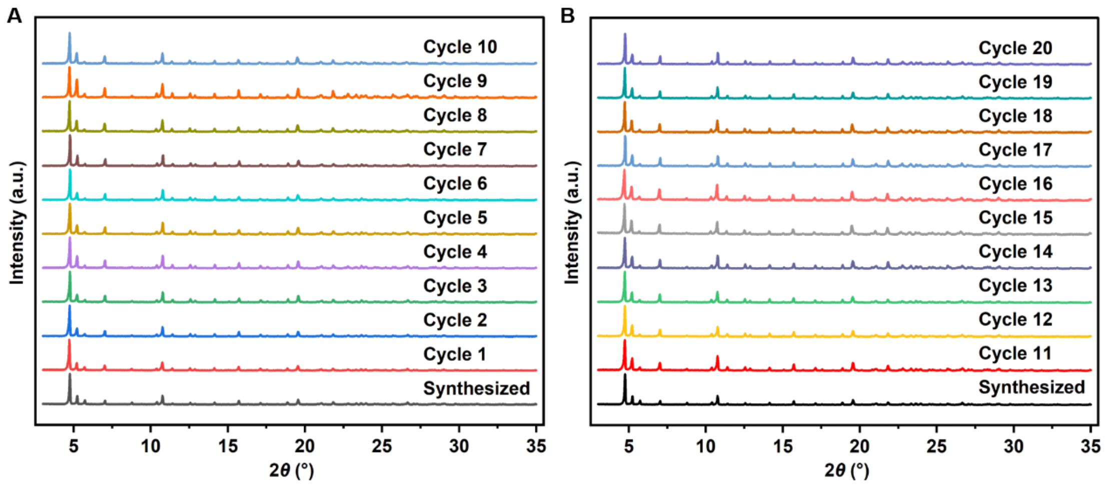

Figure S27. PXRD patterns of NKU-300 after cyclic separation experiments.

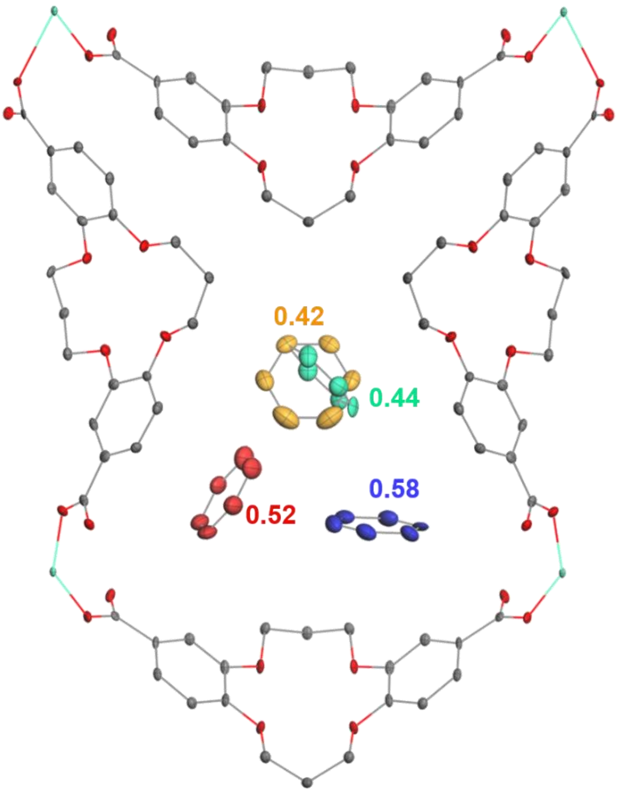

Figure S28. The SCXRD structure of benzene@NKU-300_100K. The numbers represent the occupancies. The atoms are represented by their 50% probability thermal ellipsoids. Hydrogen atoms was omitted for clarity. A summary of crystallography data was listed in Table S5.

Table S5. Crystal data and structure refinement for benzene@NKU-300_100K. Standard deviations are expressed in brackets in terms of the least significant digit(s).

Complexes	benzene@NKU-300_100K
Formula	$C_{35.88}H_{32.88}EuO_{12}$
Formula weight	808.02
Temperature (K)	100
Wavelength (Å)	1.54184
Crystal system	Monoclinic
Space group	$P2_1/m$
a (Å)	4.87652(5)
b (Å)	37.4623(4)
c (Å)	17.3923(2)
α (°)	90.00
β (°)	96.9525(10)
γ (°)	90.00
V (Å^3)	3153.95(6)
F(000)	1629.0
Z	4
ρ_{calc} (g cm^{-3})	1.702
μ (mm^{-1})	14.825
2θ range (°)	4.718–140.148
Ref. meas. / indep.	21083/6014
R_{int}	0.0625
$^aR_1$ / $^bwR_2$ [$I \geq 2\sigma(I)$]	0.0378/0.0898
$^aR_1$ / $^bwR_2$ (all data)	0.0479/0.0948
GOF on F^2	1.018
Largest diff. peak/hole (e.Å^{-3})	1.11/-1.39
CCDC	2320898

$^a R_1 = \Sigma||F_o| - |F_c||/\Sigma|F_o|$. $^b wR_2 = [\Sigma w(F_o^2 - F_c^2)^2/\Sigma w(F_o^2)^2]^{1/2}$.

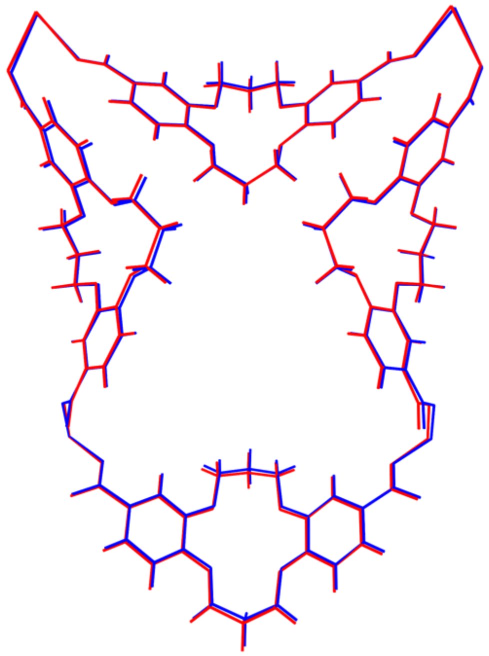

Figure S29. Structure of NKU-300 before (blue line) and after (red line) the adsorption of benzene.

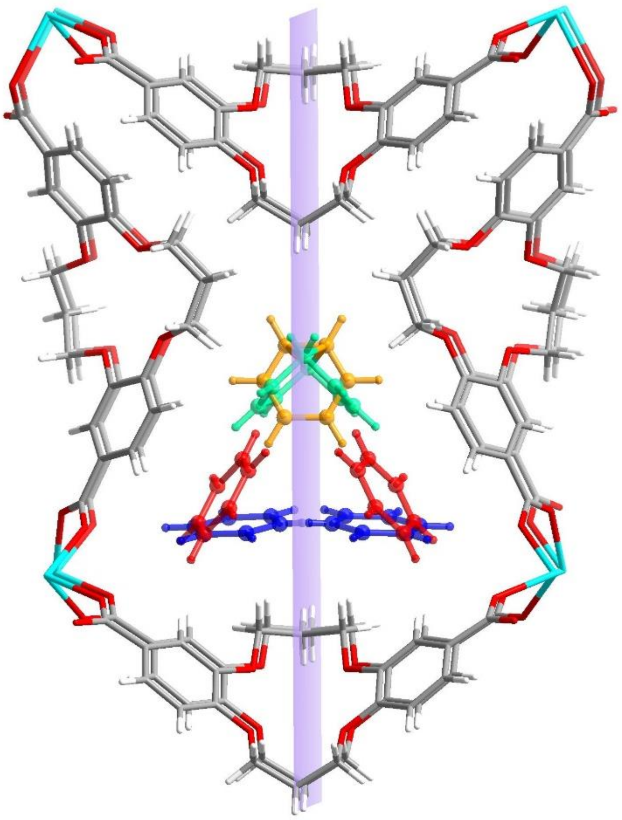

Figure S30. The mirror plane (purple) in the structure of benzene@NKU-300.

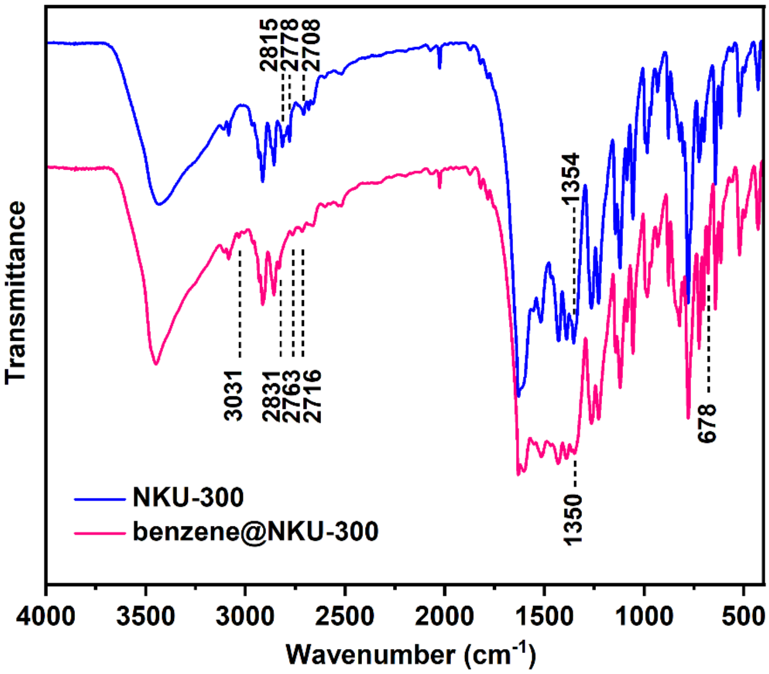

Figure S31. The FT-IR spectra of NKU-300 before and after immersing in benzene.

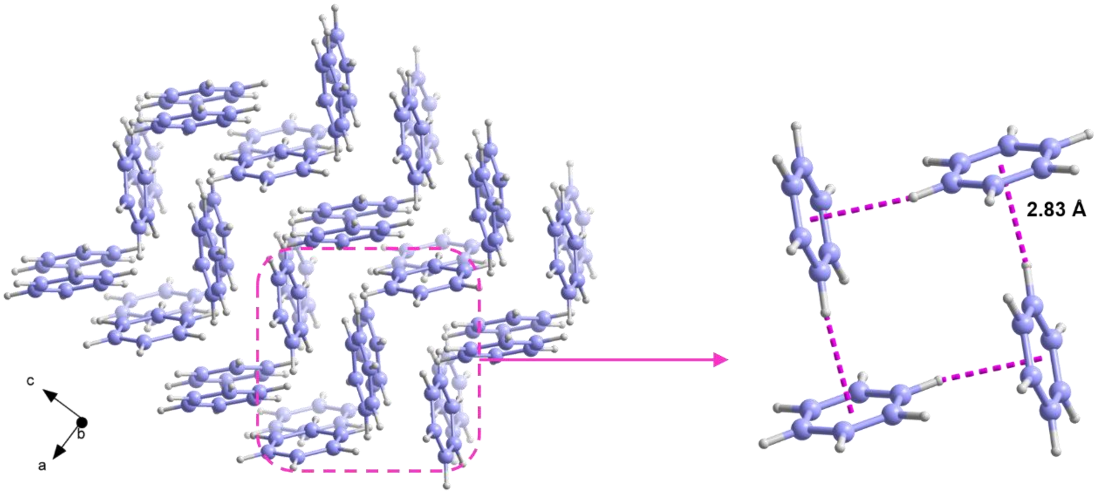

Figure S32. The structure of solid benzene [19,20].

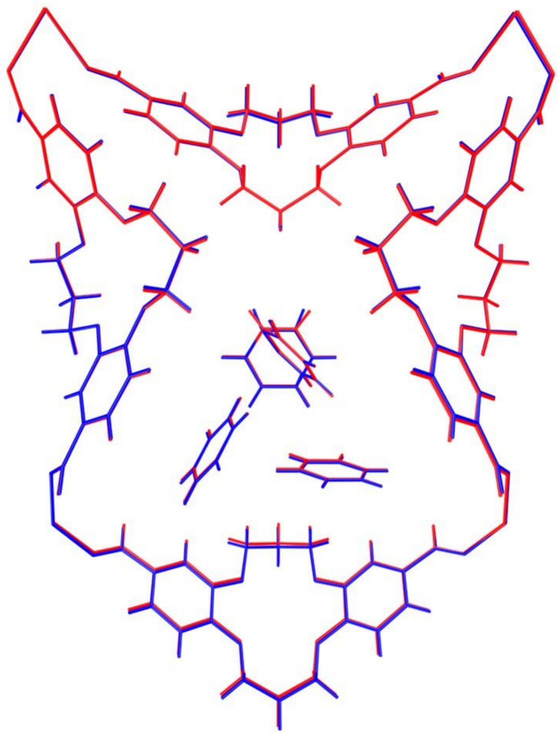

Figure S33. Structure of benzene@NKU-300 prepared by soaking in pure benzene (blue line) and benzene/cyclohexane = 1:100 (red line). A summary of crystallography data was listed in Table S6.

Table S6. Crystal Data and Structure Refinement for benzene@NKU-300_100K-1.

Complexes	benzene@NKU-300_100K-1
Formula	$C_{35.64}H_{32.64}EuO_{12}$
Formula weight	804.90
Temperature (K)	100
Wavelength (Å)	1.54184
Crystal system	Monoclinic
Space group	$P2_1/m$
a (Å)	4.87760(10)
b (Å)	37.4518(8)
c (Å)	17.3746(4)
α (°)	90.00
β (°)	96.878(2)
γ (°)	90.00
V (Å^3)	3151.06(12)
$F(000)$	1622.0
Z	4
ρ_{calc} (g cm^{-3})	1.697
μ (mm^{-1})	14.836
2θ range (°)	6.968–140.114
Ref. meas. / indep.	20942/6021
R_{int}	0.0796
$^aR_1$ / $^bwR_2$ [$I \geq 2\sigma(I)$]	0.0286/0.0675
$^aR_1$ / $^bwR_2$ (all data)	0.0336/0.0691
GOF on F^2	1.069
Largest diff. peak/hole (e.Å^{-3})	0.78/-0.60
CCDC	2320924

$^a R_1 = \Sigma||F_o| - |F_c||/\Sigma|F_o|$. $^b wR_2 = [\Sigma w(F_o^2 - F_c^2)^2/\Sigma w(F_o^2)^2]^{1/2}$.

Table S7. Summary of unit cell parameters. The monoclinic crystal system and the space group $P2_1/m$ remain unaltered throughout the measurement. Standard deviations are expressed in brackets in terms of the least significant digit(s).

T (K)	Condition	*a* (Å)	*b* (Å)	*c* (Å)	*β* (°)	*V* (Å^3)
100	/	4.87652(5)	37.4623(4)	17.39225(19)	96.9525(10)	3153.95(6)
180	/	4.889	37.5570(2)	17.36670(10)	96.75	3166.70(2)
298	/	4.912	37.76070(10)	17.38420(10)	96.65	3202.74(2)
298	dirty vacuum	4.919	37.77620(10)	17.38280(10)	96.64	3208.43(2)
298	fine vacuum	4.926	37.7926(2)	17.38410(10)	96.62	3214.76(3)
323	fine vacuum	4.936	37.8422(3)	17.39130(10)	96.5960(10)	3227.00(3)
323	argon 200 mbar	4.942	37.8537(3)	17.39300(10)	96.5810(10)	3232.32(3)
353	argon 200 mbar	4.9478(2)	37.9632(12)	17.4290(6)	96.493(4)	3252.8(2)
393	argon 200 mbar	4.93240(10)	37.8682(8)	17.3012(4)	96.360(2)	3211.65(12)
423	argon 200 mbar	4.923	37.7958(5)	17.1877(2)	96.0980(10)	3180.00(6)
273	argon 200 mbar	4.910	37.5762(3)	17.14420(10)	96.1480(10)	3144.90(3)

Table S8. Summary of refinement details.

T (K)	Condition	Resolution (Å)	R_{int} (%)	R_1 [$I \geq 2\sigma(I)$] (%)	wR_2 (all data) (%)	GooF
100	/	0.82	6.25	3.78	9.48	1.018
180	/	0.78	3.24	1.85	5.21	1.073
298	/	0.78	3.39	1.69	4.68	1.057
298	dirty vacuum	0.78	3.37	1.76	4.96	1.044
298	fine vacuum	0.78	3.43	1.86	5.09	1.013
323	fine vacuum	0.78	3.98	2.13	5.79	0.949
323	argon 200 mbar	0.80	4.50	2.53	6.77	0.972
353	argon 200 mbar	0.80	25.74	8.42	21.93	0.986
393	argon 200 mbar	0.78	13.34	4.80	12.43	1.047
423	argon 200 mbar	0.77	9.54	2.96	5.74	0.984
273	argon 200 mbar	0.80	5.71	5.01	13.98	0.978

Table S9. Summary of the occupancies of the benzene molecules at sites a to d.

T (K)	Condition	Occupancies				Number / per cage
		a	b	c	d	
100	/	0.58	0.52	0.44	0.42	1.96
180	/	0.50	0.46	0.38	0.23, 0.15	1.72
298	/	0.46	0.42	0.26	0.32	1.46
298	dirty vacuum	0.46	0.42	0.24	0.30	1.42
298	fine vacuum	0.46	0.42	0.2	0.26	1.34
323	fine vacuum	0.34	0.42	0	0.3	1.06
323	argon 200 mbar	0	0.40	0	0.22	0.62
353	argon 200 mbar	/	/	/	/	/
393	argon 200 mbar	/	/	/	/	/
423	argon 200 mbar	/	/	/	/	/
273	argon 200 mbar	/	/	/	/	/

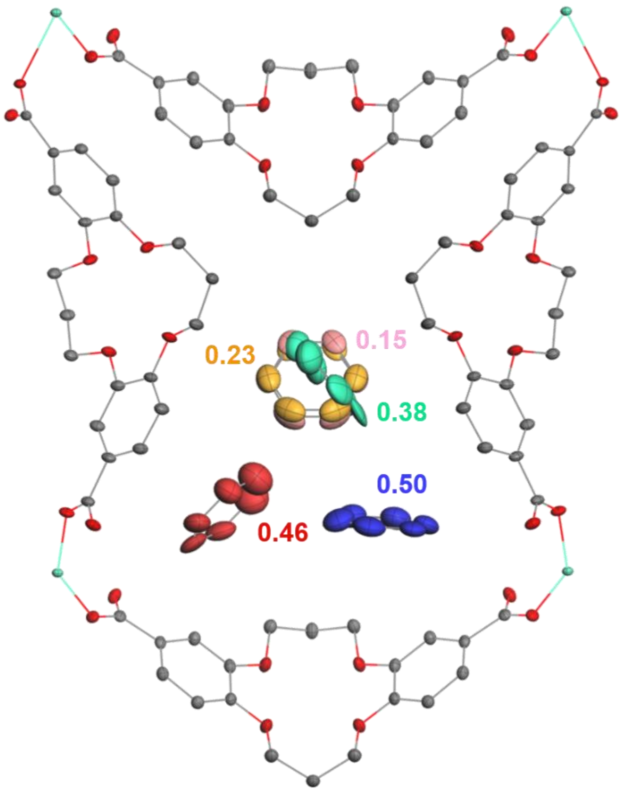

Figure S34. The SCXRD structure of benzene@NKU-300_180K. The numbers represent the occupancies. The atoms are represented by their 50% probability thermal ellipsoids. Hydrogen atoms was omitted for clarity.

Table S10. Crystal Data and Structure Refinement for benzene@NKU-300_180K.

Complexes	benzene@NKU-300_180K
Formula	$C_{35.16}H_{32.16}EuO_{12}$
Formula weight	798.65
Temperature (K)	180
Wavelength (Å)	0.4859
Crystal system	Monoclinic
Space group	$P2_1/m$
a (Å)	4.889
b (Å)	37.5570(2)
c (Å)	17.36670(10)
α (°)	90.00
β (°)	96.75
γ (°)	90.00
V (Å^3)	3166.70(2)
F(000)	1608.0
Z	4
ρ_{calc} (g cm^{-3})	1.675
μ (mm^{-1})	0.744
2θ range (°)	1.482–36.292
Ref. meas. / indep.	47678/7084
R_{int}	0.0324
$^aR_1$ / $^bwR_2$ [$I \geq 2\sigma(I)$]	0.0185/0.0513
$^aR_1$ / $^bwR_2$ (all data)	0.0203/0.0521
GOF on F^2	1.073
Largest diff. peak/hole (e.Å^{-3})	0.59/-1.04
CCDC	2320926

$^a R_1 = \Sigma||F_o| - |F_c||/\Sigma|F_o|$. $^b wR_2 = [\Sigma w(F_o^2 - F_c^2)^2/\Sigma w(F_o^2)^2]^{1/2}$.

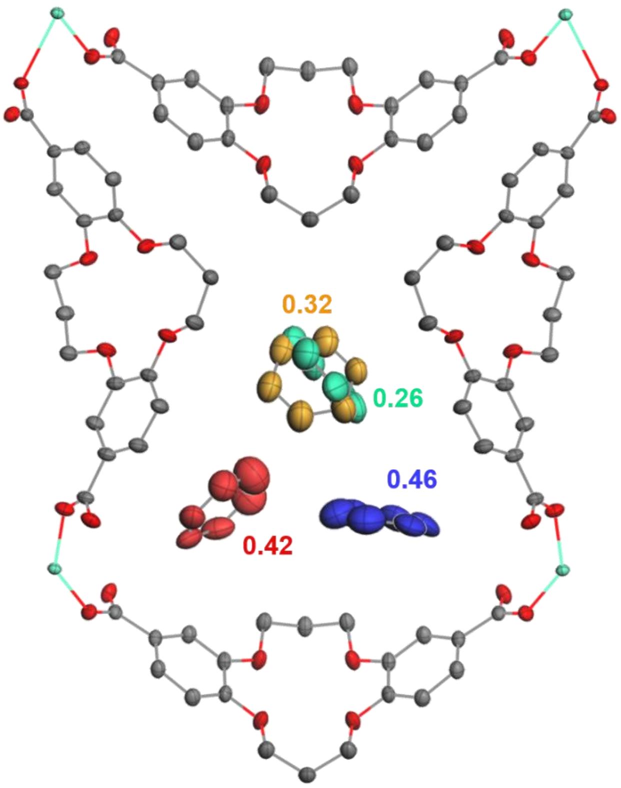

Figure S35. The SCXRD structure of benzene@NKU-300_298K-1. The numbers represent the occupancies. The atoms are represented by their 50% probability thermal ellipsoids. Hydrogen atoms was omitted for clarity.

Table S11. Crystal Data and Structure Refinement for benzene@NKU-300_298K-1.

Complexes	benzene@NKU-300_298K-1
Formula	$C_{34.38}H_{31.38}EuO_{12}$
Formula weight	788.49
Temperature (K)	298
Wavelength (Å)	0.4859
Crystal system	Monoclinic
Space group	$P2_1/m$
a (Å)	4.912
b (Å)	37.76070(10)
c (Å)	17.38420(10)
α (°)	90.00
β (°)	96.65
γ (°)	90.00
V (Å^3)	3202.74(2)
F(000)	1587.0
Z	4
ρ_{calc} (g cm^{-3})	1.635
μ (mm^{-1})	0.735
2θ range (°)	1.474–36.296
Ref. meas. / indep.	48702/7187
R_{int}	0.0339
$^aR_1$ / $^bwR_2$ [$I \geq 2\sigma(I)$]	0.0169/0.0463
$^aR_1$ / $^bwR_2$ (all data)	0.0202/0.0468
GOF on F^2	1.057
Largest diff. peak/hole (e.Å^{-3})	0.42/-0.35
CCDC	2320932

$^a R_1 = \Sigma||F_o| - |F_c||/\Sigma|F_o|$. $^b wR_2 = [\Sigma w(F_o^2 - F_c^2)^2/\Sigma w(F_o^2)^2]^{1/2}$.

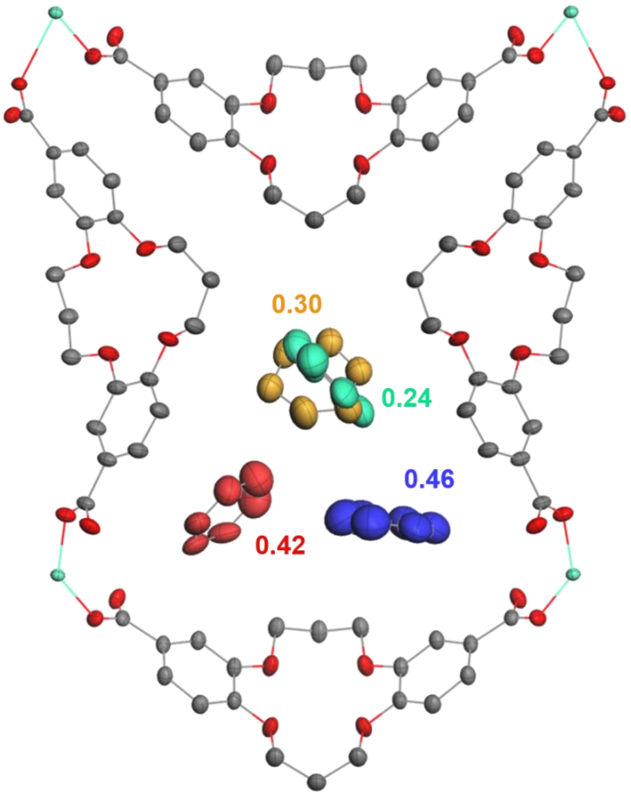

Figure S36. The SCXRD structure of benzene@NKU-300_298K-2. The numbers represent the occupancies. The atoms are represented by their 50% probability thermal ellipsoids. Hydrogen atoms was omitted for clarity.

Table S12. Crystal Data and Structure Refinement for benzene@NKU-300_298K-2.

Complexes	benzene@NKU-300_298K-2
Formula	$C_{34.26}H_{31.26}EuO_{12}$
Formula weight	786.93
Temperature (K)	298
Wavelength (Å)	0.4859
Crystal system	Monoclinic
Space group	$P2_1/m$
a (Å)	4.919
b (Å)	37.77620(10)
c (Å)	17.38280(10)
α (°)	90.00
β (°)	96.64
γ (°)	90.00
V (Å^3)	3208.43(2)
F(000)	1583.0
Z	4
ρ_{calc} (g cm^{-3})	1.629
μ (mm^{-1})	0.733
2θ range (°)	1.474–36.29
Ref. meas. / indep.	48722/7195
R_{int}	0.0337
$^aR_1$ / $^bwR_2$ [$I \geq 2\sigma(I)$]	0.0176/0.0490
$^aR_1$ / $^bwR_2$ (all data)	0.0213/0.0496
GOF on F^2	1.044
Largest diff. peak/hole (e.Å^{-3})	0.45/-0.36
CCDC	2320933

$^a R_1 = \Sigma||F_o| - |F_c||/\Sigma|F_o|$. $^b wR_2 = [\Sigma w(F_o^2 - F_c^2)^2/\Sigma w(F_o^2)^2]^{1/2}$.

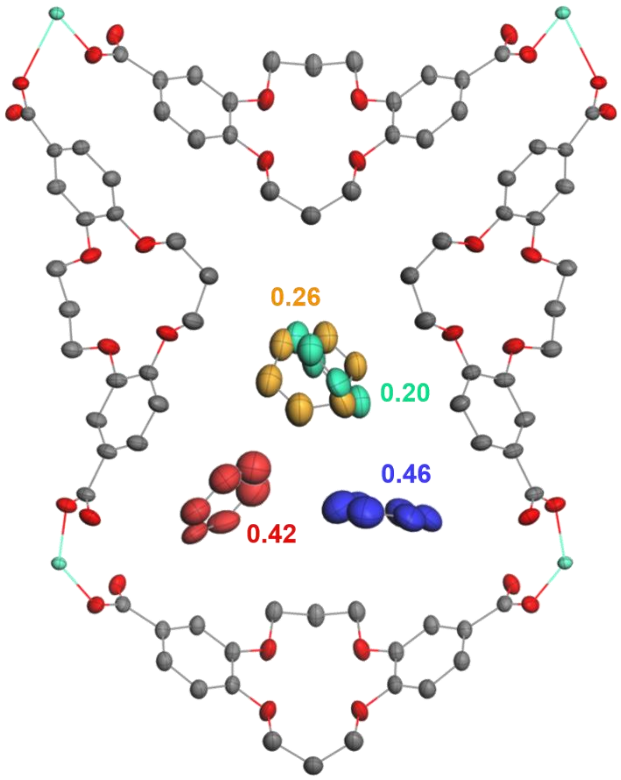

Figure S37. The SCXRD structure of benzene@NKU-300_298K-3. The numbers represent the occupancies. The atoms are represented by their 50% probability thermal ellipsoids. Hydrogen atoms was omitted for clarity.

Table S13. Crystal Data and Structure Refinement for benzene@NKU-300_298K-3.

Complexes	benzene@NKU-300_298K-3
Formula	$C_{34.02}H_{31.02}EuO_{12}$
Formula weight	783.81
Temperature (K)	298
Wavelength (Å)	0.4859
Crystal system	Monoclinic
Space group	$P2_1/m$
a (Å)	4.926
b (Å)	37.7926(2)
c (Å)	17.38410(10)
α (°)	90.00
β (°)	96.62
γ (°)	90.00
V (Å^3)	3214.76(3)
F(000)	1577.0
Z	4
ρ_{calc} (g cm^{-3})	1.619
μ (mm^{-1})	0.732
2θ range (°)	1.474–36.294
Ref. meas. / indep.	48826/7209
R_{int}	0.0343
$^aR_1$ / $^bwR_2$ [$I \geq 2\sigma(I)$]	0.0186/0.0500
$^aR_1$ / $^bwR_2$ (all data)	0.0233/0.0509
GOF on F^2	1.013
Largest diff. peak/hole (e.Å^{-3})	0.40/-0.44
CCDC	2320934

$^a R_1 = \Sigma||F_o| - |F_c||/\Sigma|F_o|$. $^b wR_2 = [\Sigma w(F_o^2 - F_c^2)^2/\Sigma w(F_o^2)^2]^{1/2}$.

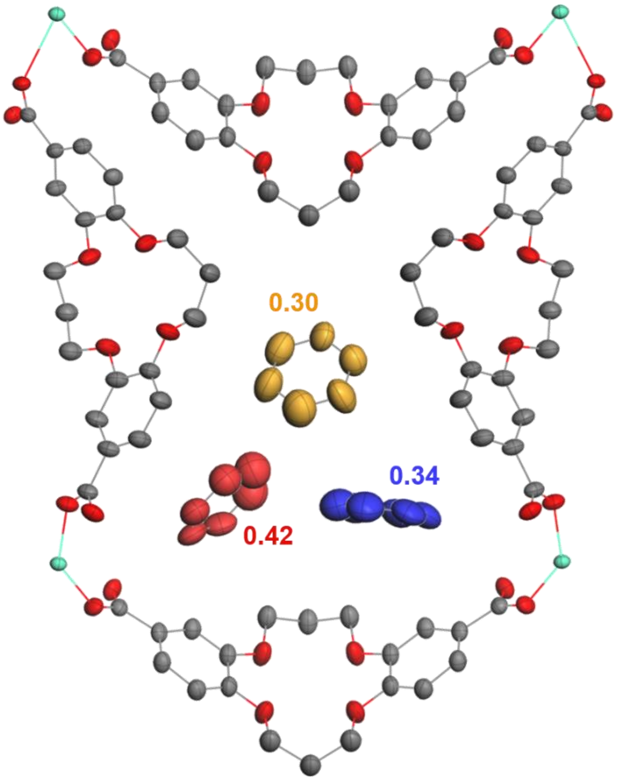

Figure S38. The SCXRD structure of benzene@NKU-300_323K-1. The numbers represent the occupancies. The atoms are represented by their 50% probability thermal ellipsoids. Hydrogen atoms was omitted for clarity.

Table S14. Crystal Data and Structure Refinement for benzene@NKU-300_323K-1.

Complexes	benzene@NKU-300_323K-1
Formula	$C_{33.18}H_{30.18}EuO_{12}$
Formula weight	772.87
Temperature (K)	323
Wavelength (Å)	0.4859
Crystal system	Monoclinic
Space group	$P2_1/m$
a (Å)	4.936
b (Å)	37.8422(3)
c (Å)	17.39130(10)
α (°)	90.00
β (°)	96.5960(10)
γ (°)	90.00
V (Å^3)	3227.00(3)
F(000)	1553.0
Z	4
ρ_{calc} (g cm^{-3})	1.591
μ (mm^{-1})	0.728
2θ range (°)	1.612–36.296
Ref. meas. / indep.	48954/7235
R_{int}	0.0398
$^aR_1$ / $^bwR_2$ [$I \geq 2\sigma(I)$]	0.0213/0.0563
$^aR_1$ / $^bwR_2$ (all data)	0.0288/0.0579
GOF on F^2	0.949
Largest diff. peak/hole (e.Å^{-3})	0.52/-0.60
CCDC	2321059

$^a R_1 = \Sigma||F_o| - |F_c||/\Sigma|F_o|$. $^b wR_2 = [\Sigma w(F_o^2 - F_c^2)^2/\Sigma w(F_o^2)^2]^{1/2}$.

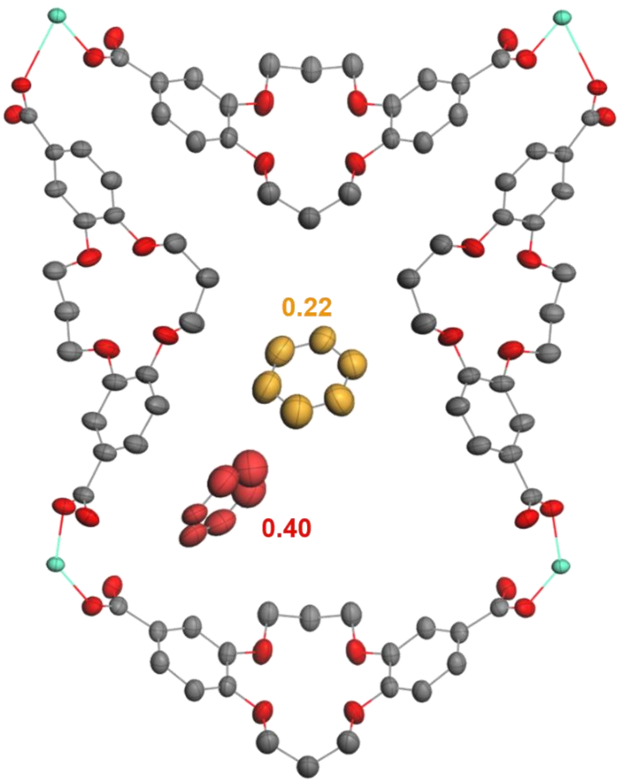

Figure S39. The SCXRD structure of benzene@NKU-300_323K-2. The numbers represent the occupancies. The atoms are represented by their 50% probability thermal ellipsoids. Hydrogen atoms was omitted for clarity.

Table S15. Crystal Data and Structure Refinement for benzene@NKU-300_323K-2.

Complexes	benzene@NKU-300_323K-2
Formula	$C_{31.86}H_{28.86}EuO_{12}$
Formula weight	755.69
Temperature (K)	323
Wavelength (Å)	0.4859
Crystal system	Monoclinic
Space group	$P2_1/m$
a (Å)	4.942
b (Å)	37.8537(3)
c (Å)	17.39300(10)
α (°)	90.00
β (°)	96.5810(10)
γ (°)	90.00
V (Å^3)	3232.32(3)
F(000)	1516.0
Z	4
ρ_{calc} (g cm^{-3})	1.553
μ (mm^{-1})	0.726
2θ range (°)	1.612–35.358
Ref. meas. / indep.	45306/6686
R_{int}	0.0450
$^aR_1$ / $^bwR_2$ [$I \geq 2\sigma(I)$]	0.0253/0.0658
$^aR_1$ / $^bwR_2$ (all data)	0.0341/0.0677
GOF on F^2	0.972
Largest diff. peak/hole (e.Å^{-3})	0.47/-0.84
CCDC	2321060

$^a R_1 = \Sigma||F_o| - |F_c||/\Sigma|F_o|$. $^b wR_2 = [\Sigma w(F_o^2 - F_c^2)^2/\Sigma w(F_o^2)^2]^{1/2}$.

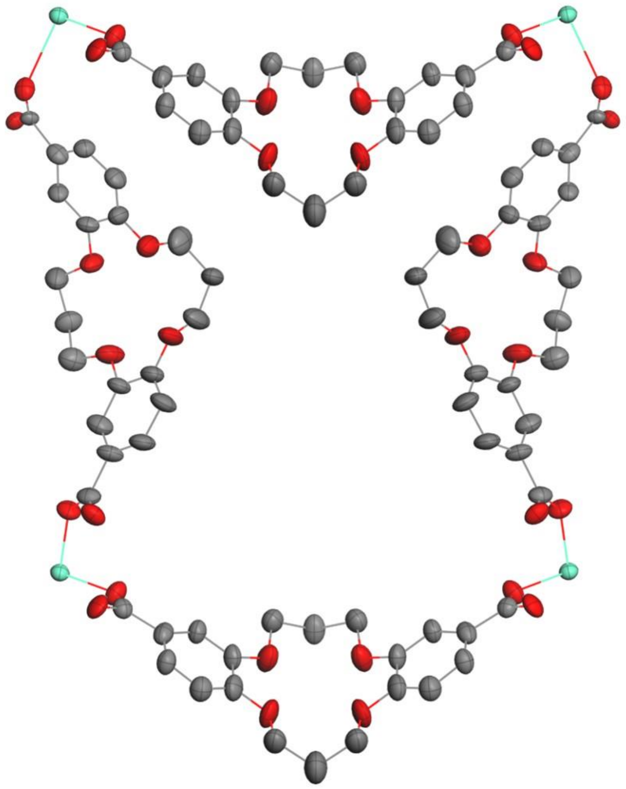

Figure S40. The SCXRD structure of benzene@NKU-300_353K. The numbers represent the occupancies. The atoms are represented by their 50% probability thermal ellipsoids. Hydrogen atoms was omitted for clarity.

Table S16. Crystal Data and Structure Refinement for benzene@NKU-300_353K.

Complexes	benzene@NKU-300_353K
Formula	$C_{30}H_{27}EuO_{12}$
Formula weight	731.47
Temperature (K)	353
Wavelength (Å)	0.4859
Crystal system	Monoclinic
Space group	$P2_1/m$
a (Å)	4.9478(2)
b (Å)	37.9632(12)
c (Å)	17.4290(6)
α (°)	90.00
β (°)	96.493(4)
γ (°)	90.00
V (Å^3)	3252.8(2)
F(000)	1464.0
Z	4
ρ_{calc} (g cm^{-3})	1.494
μ (mm^{-1})	0.720
2θ range (°)	3.346–35.356
Ref. meas. / indep.	26880/6737
R_{int}	0.2574
$^aR_1$ / $^bwR_2$ [$I \geq 2\sigma(I)$]	0.0842/0.1889
$^aR_1$ / $^bwR_2$ (all data)	0.1367/0.2193
GOF on F^2	0.986
Largest diff. peak/hole (e.Å^{-3})	2.47/-2.59
CCDC	2321066

$^a R_1 = \Sigma||F_o| - |F_c||/\Sigma|F_o|$. $^b wR_2 = [\Sigma w(F_o^2 - F_c^2)^2/\Sigma w(F_o^2)^2]^{1/2}$.

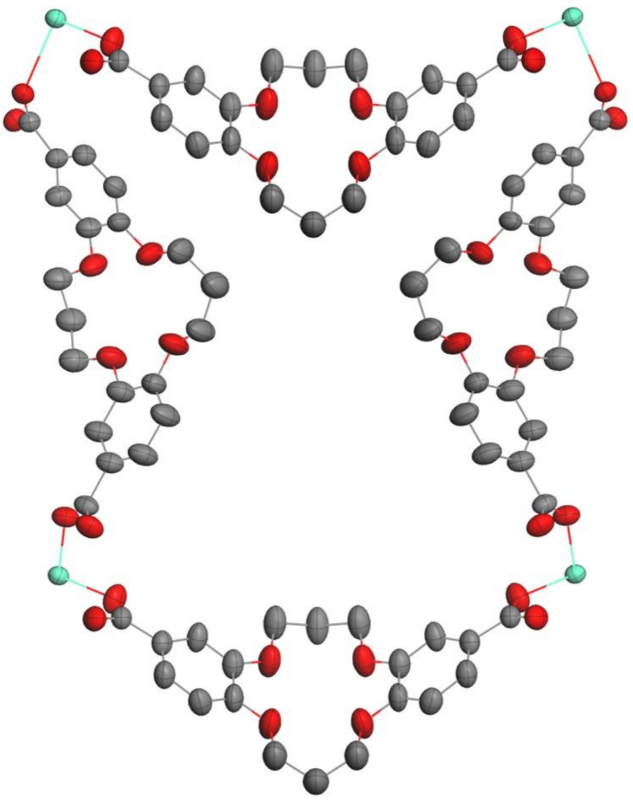

Figure S41. The SCXRD structure of benzene@NKU-300_393K. The numbers represent the occupancies. The atoms are represented by their 50% probability thermal ellipsoids. Hydrogen atoms was omitted for clarity.

Table S17. Crystal Data and Structure Refinement for benzene@NKU-300_393K.

Complexes	benzene@NKU-300_393K
Formula	$C_{30}H_{27}EuO_{12}$
Formula weight	731.47
Temperature (K)	393
Wavelength (Å)	0.4859
Crystal system	Monoclinic
Space group	$P2_1/m$
a (Å)	4.93240(10)
b (Å)	37.8682(8)
c (Å)	17.3012(4)
α (°)	90.00
β (°)	96.360(2)
γ (°)	90.00
V (Å^3)	3211.65(12)
F(000)	1464.0
Z	4
ρ_{calc} (g cm^{-3})	1.513
μ (mm^{-1})	0.729
2θ range (°)	3.322–36.296
Ref. meas. / indep.	44681/7199
R_{int}	0.1334
$^aR_1$ / $^bwR_2$ [$I \geq 2\sigma(I)$]	0.0480/0.1165
$^aR_1$ / $^bwR_2$ (all data)	0.0649/0.1243
GOF on F^2	1.047
Largest diff. peak/hole (e.Å^{-3})	1.73/-1.43
CCDC	2321067

$^a R_1 = \Sigma||F_o| - |F_c||/\Sigma|F_o|$. $^b wR_2 = [\Sigma w(F_o^2 - F_c^2)^2/\Sigma w(F_o^2)^2]^{1/2}$.

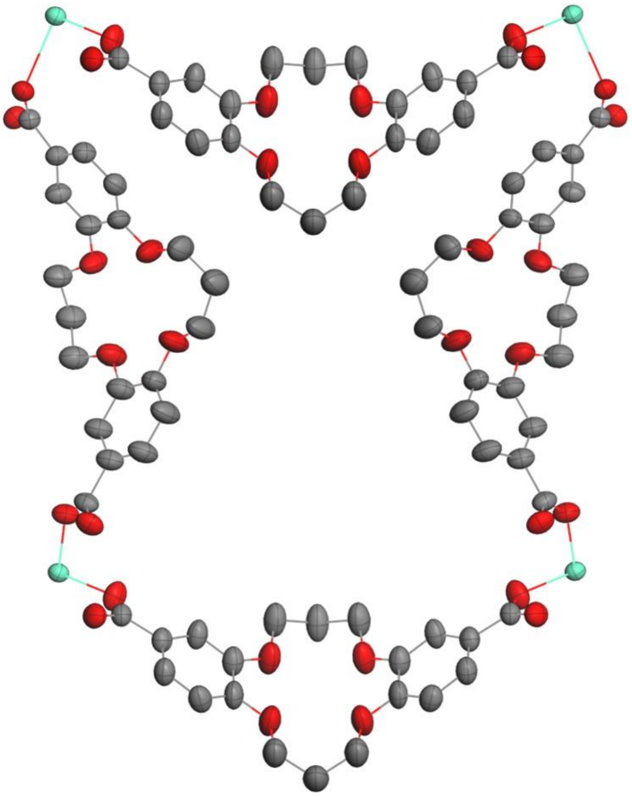

Figure S42. The SCXRD structure of benzene@NKU-300_423K. The numbers represent the occupancies. The atoms are represented by their 50% probability thermal ellipsoids. Hydrogen atoms was omitted for clarity.

Table S18. Crystal Data and Structure Refinement for benzene@NKU-300_423K.

Complexes	benzene@NKU-300_423K
Formula	$C_{30}H_{27}EuO_{12}$
Formula weight	731.47
Temperature (K)	423
Wavelength (Å)	0.4859
Crystal system	Monoclinic
Space group	$P2_1/m$
a (Å)	4.923
b (Å)	37.7958(5)
c (Å)	17.1877(2)
α (°)	90.00
β (°)	96.0980(10)
γ (°)	90.00
V (Å^3)	3180.00(6)
F(000)	1464.0
Z	4
ρ_{calc} (g cm^{-3})	1.528
μ (mm^{-1})	0.736
2θ range (°)	1.474–36.784
Ref. meas. / indep.	50017/7404
R_{int}	0.0954
$^aR_1$ / $^bwR_2$ [$I \geq 2\sigma(I)$]	0.0296/0.0561
$^aR_1$ / $^bwR_2$ (all data)	0.0461/0.0574
GOF on F^2	0.984
Largest diff. peak/hole (e.Å^{-3})	0.44/-0.45
CCDC	2321068

$^a R_1 = \Sigma||F_o| - |F_c||/\Sigma|F_o|$. $^b wR_2 = [\Sigma w(F_o^2 - F_c^2)^2/\Sigma w(F_o^2)^2]^{1/2}$.

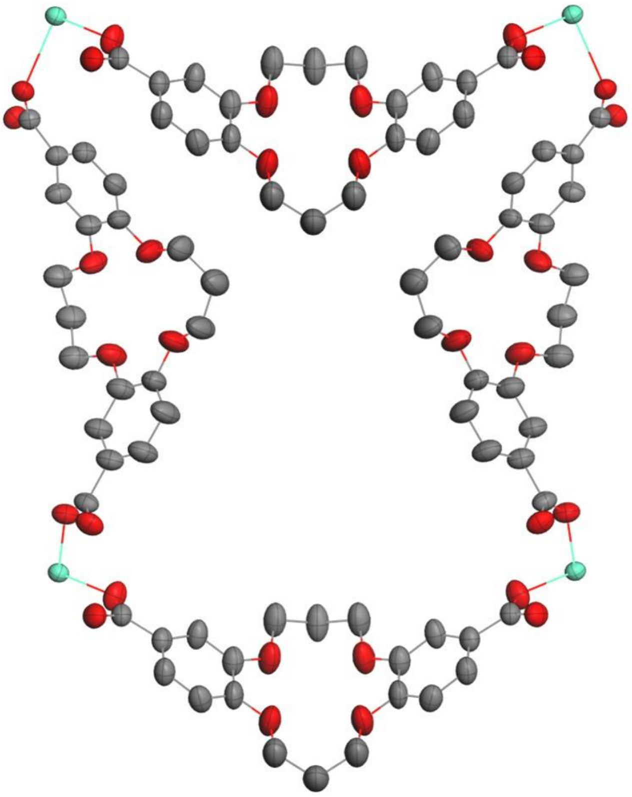

Figure S43. The SCXRD structure of benzene@NKU-300_273K. The numbers represent the occupancies. The atoms are represented by their 50% probability thermal ellipsoids. Hydrogen atoms was omitted for clarity.

Table S19. Crystal Data and Structure Refinement for benzene@NKU-300_273K.

Complexes	benzene@NKU-300_273K
Formula	$C_{30}H_{27}EuO_{12}$
Formula weight	731.47
Temperature (K)	273
Wavelength (Å)	0.4859
Crystal system	Monoclinic
Space group	$P2_1/m$
a (Å)	4.910
b (Å)	37.5762(3)
c (Å)	17.14420(10)
α (°)	90.00
β (°)	96.1480(10)
γ (°)	90.00
V (Å^3)	3144.90(3)
F(000)	1464.0
Z	4
ρ_{calc} (g cm^{-3})	1.545
μ (mm^{-1})	0.744
2θ range (°)	1.482–35.358
Ref. meas. / indep.	44232/6527
R_{int}	0.0571
$^aR_1$ / $^bwR_2$ [$I \geq 2\sigma(I)$]	0.0501/0.1365
$^aR_1$ / $^bwR_2$ (all data)	0.0586/0.1398
GOF on F^2	0.978
Largest diff. peak/hole (e.Å^{-3})	2.73/-1.59
CCDC	2320931

$^a R_1 = \Sigma||F_o| - |F_c||/\Sigma|F_o|$. $^b wR_2 = [\Sigma w(F_o^2 - F_c^2)^2/\Sigma w(F_o^2)^2]^{1/2}$.

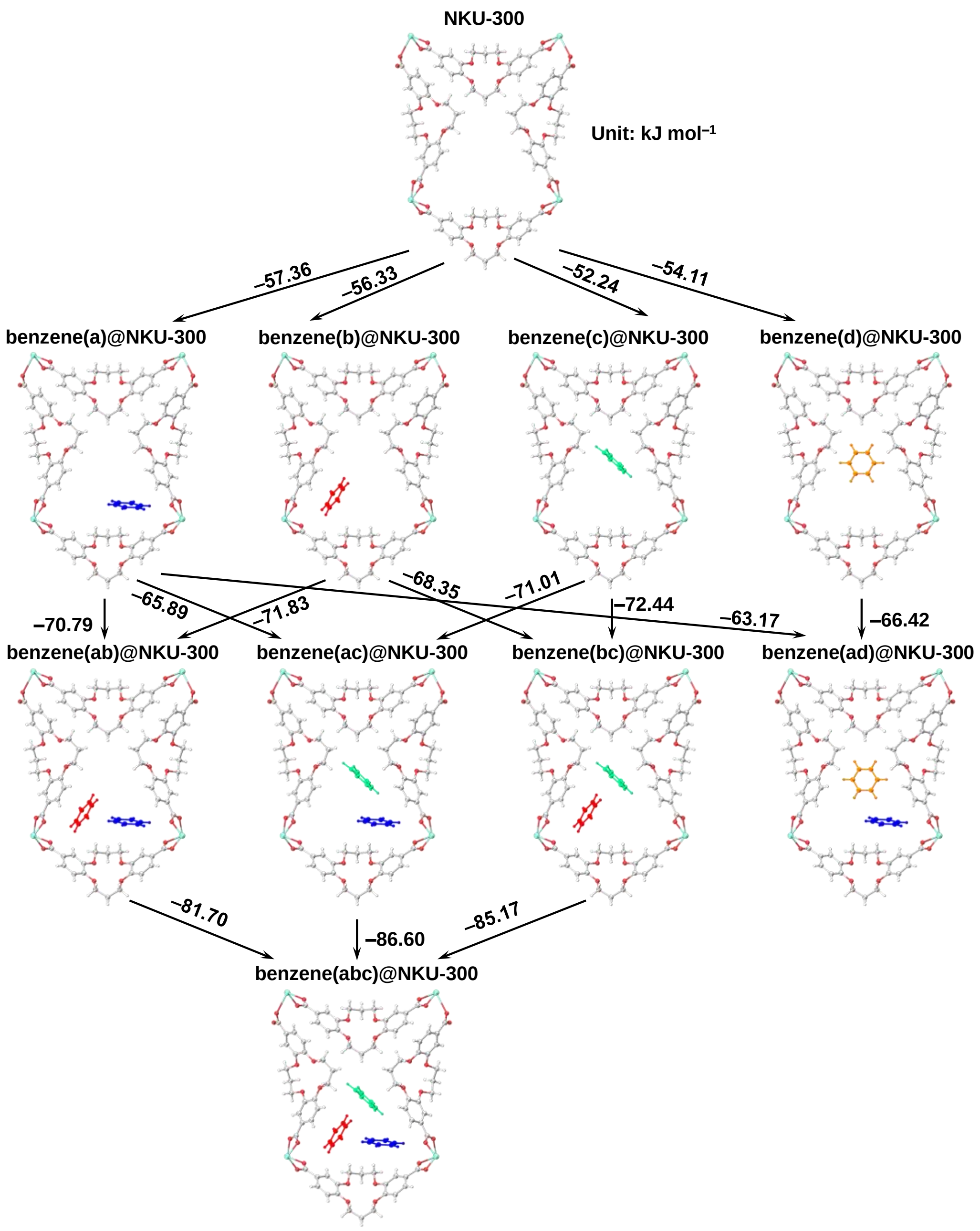

Route 1:	NKU-300→benzene(a)@NKU-300	ΔE_{ads} (kJ mol^{-1}): –57.36
Route 2:	NKU-300→benzene(b)@NKU-300	ΔE_{ads} (kJ mol^{-1}): –56.33
Route 3:	NKU-300→benzene(c)@NKU-300	ΔE_{ads} (kJ mol^{-1}): –52.24
Route 4:	NKU-300→benzene(d)@NKU-300	ΔE_{ads} (kJ mol^{-1}): –54.11
Route 5:	NKU-300→benzene(a)@NKU-300→benzene(ab)@NKU-300	ΔE_{ads} (kJ mol^{-1}): –128.15 = (–57.36) + (–70.79)
Route 6:	NKU-300→benzene(a)@NKU-300→benzene(ac)@NKU-300	ΔE_{ads} (kJ mol^{-1}): –123.25 = (–57.36) + (–65.89)
Route 7:	NKU-300→benzene(b)@NKU-300→benzene(ab)@NKU-300	ΔE_{ads} (kJ mol^{-1}): –128.16 = (–56.33) + (–71.83)
Route 8:	NKU-300→benzene(b)@NKU-300→benzene(bc)@NKU-300	ΔE_{ads} (kJ mol^{-1}): –124.68 = (–56.33) + (–68.35)
Route 9:	NKU-300→benzene(c)@NKU-300→benzene(ac)@NKU-300	ΔE_{ads} (kJ mol^{-1}): –123.25 = (–52.24) + (–71.01)
Route 10:	NKU-300→benzene(c)@NKU-300→benzene(bc)@NKU-300	ΔE_{ads} (kJ mol^{-1}): –124.68 = (–52.24) + (–72.44)
Route 11:	NKU-300→benzene(a)@NKU-300→benzene(ad)@NKU-300	ΔE_{ads} (kJ mol^{-1}): –120.53 = (–57.36) + (–63.17)
Route 12:	NKU-300→benzene(d)@NKU-300→benzene(ad)@NKU-300	ΔE_{ads} (kJ mol^{-1}): –120.53 = (–54.11) + (–66.42)
Route 13:	NKU-300→benzene(a)@NKU-300→benzene(ab)@NKU-300→benzene(abc)@NKU-300	ΔE_{ads} (kJ mol^{-1}): –209.85 = (–57.36) + (–70.79) + (–81.70)
Route 14:	NKU-300→benzene(a)@NKU-300→benzene(ac)@NKU-300→benzene(abc)@NKU-300	ΔE_{ads} (kJ mol^{-1}): –209.85 = (–57.36) + (–65.89) + (–86.60)
Route 15:	NKU-300→benzene(b)@NKU-300→benzene(ab)@NKU-300→benzene(abc)@NKU-300	ΔE_{ads} (kJ mol^{-1}): –209.86 = (–56.33) + (–71.83) + (–81.70)
Route 16:	NKU-300→benzene(b)@NKU-300→benzene(bc)@NKU-300→benzene(abc)@NKU-300	ΔE_{ads} (kJ mol^{-1}): –209.85 = (–56.33) + (–68.35) + (–85.17)
Route 17:	NKU-300→benzene(c)@NKU-300→benzene(ac)@NKU-300→benzene(abc)@NKU-300	ΔE_{ads} (kJ mol^{-1}): –209.85 = (–52.24) + (–71.01) + (–86.60)
Route 18:	NKU-300→benzene(c)@NKU-300→benzene(bc)@NKU-300→benzene(abc)@NKU-300	ΔE_{ads} (kJ mol^{-1}): –209.85 = (–52.24) + (–72.44) + (–85.17)

Figure S44. The adsorption routes of benzene in benzene@NKU-300 and the binding energy of each adsorption step (marked on the arrow). The 18 adsorption routes and the calculation formula of binding energy are listed below the figure.

Table S20. Calculated total energy and average binding energy of different structures based on benzene@NKU-300.

Structure	E (hartree)	ΔE_{ads} (kJ mol^{-1})
NKU-300[a]	–11265.2217484	
benzene(a)	–231.734399624	
benzene(b)	–231.877065930	
benzene(c)	–231.858450623	
benzene(d)	–231.812193757	
benzene(a)@NKU-300	–11496.9779982	–57.36
benzene(b)@NKU-300	–11497.1202703	–56.33
benzene(c)@NKU-300	–11497.1000978	–52.24
benzene(d)@NKU-300	–11497.0545528	–54.11
benzene(ab)@NKU-300	–11728.8820272	–64.08
benzene(ac)@NKU-300	–11728.8615449	–61.63
benzene(bc)@NKU-300	–11729.0047551	–62.34
benzene(ad)@NKU-300	–11728.8142518	–60.27
benzene(abc)@NKU-300	–11960.7715956	–69.95

[a]The structure of NKU-300 is extracted from the structure of benzene@NKU-300.

Adsorption configuration analysis

Considering both saturated and unsaturated adsorption conditions, benzene@NKU-300 exhibits nine adsorption configurations (Fig. S44). The binding energy of each configuration was calculated to be –52.2 ~ –70.0 kJ mol^{-1} (Table S20), which demonstrates the increase from mono-benzene adsorption to di-benzene and tri-benzene adsorption. Considering the sequence of benzene adsorption one by one (Fig. S44), the calculated binding energy of each step (Table S21) indicates guest-guest interaction boosting the binding energy. the average binding energy value (ΔE_x, Table S21) of each adsorption site could be calculated to verify the sequence of benzene desorption.

Table S21. Binding energy of different adsorption steps of benzene@NKU-300.

Step	ΔE_{ads} (kJ mol^{-1})
NKU-300[a]→benzene(a)@NKU-300	–57.36
NKU-300[a]→benzene(b)@NKU-300	–56.33
NKU-300[a]→benzene(c)@NKU-300	–52.24
NKU-300[a]→benzene(d)@NKU-300	–54.11
benzene(a)@NKU-300→benzene(ab)@NKU-300	–70.79
benzene(a)@NKU-300→benzene(ac)@NKU-300	–65.89
benzene(b)@NKU-300→benzene(ab)@NKU-300	–71.83
benzene(b)@NKU-300→benzene(bc)@NKU-300	–68.35
benzene(c)@NKU-300→benzene(ac)@NKU-300	–71.01
benzene(c)@NKU-300→benzene(bc)@NKU-300	–72.44
benzene(a)@NKU-300→benzene(ad)@NKU-300	–63.17
benzene(d)@NKU-300→benzene(ad)@NKU-300	–66.42
benzene(ab)@NKU-300→benzene(abc)@NKU-300	–81.70
benzene(ac)@NKU-300→benzene(abc)@NKU-300	–86.60
benzene(bc)@NKU-300→benzene(abc)@NKU-300	–85.17

[a]The structure of NKU-300 is extracted from the structure of benzene@NKU-300.

Table S22. Calculated average binding energy (ΔE) of different benzene adsorption sites in benzene@NKU-300.

Adsorption site	ΔE_{ads}[a] (kJ mol^{-1})					ΔE (kJ mol^{-1})
	1	2	3	4	5	
a	–57.36	–71.83	–71.01	–66.42	–85.17	–70.36
b	–56.33	–70.79	–72.44	–86.60		–71.54
c	–52.24	–65.89	–68.35	–81.70		–67.05
d	–54.11	–63.17				–58.64

[a]The binding energies are extracted from Table S21.

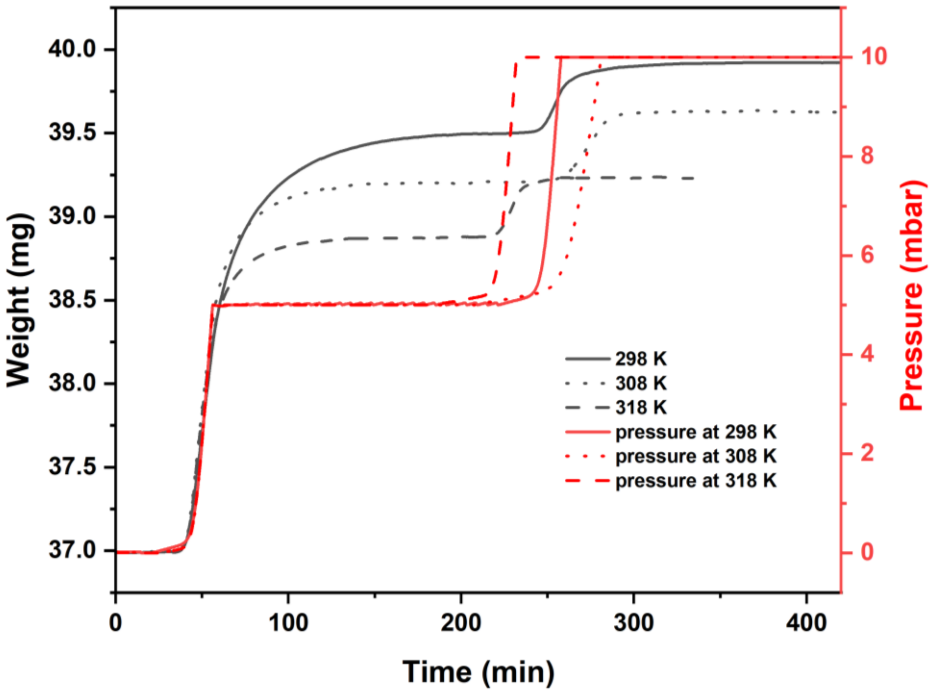

Figure S45. The adsorption kinetics of benzene by NKU-300 at different temperature.

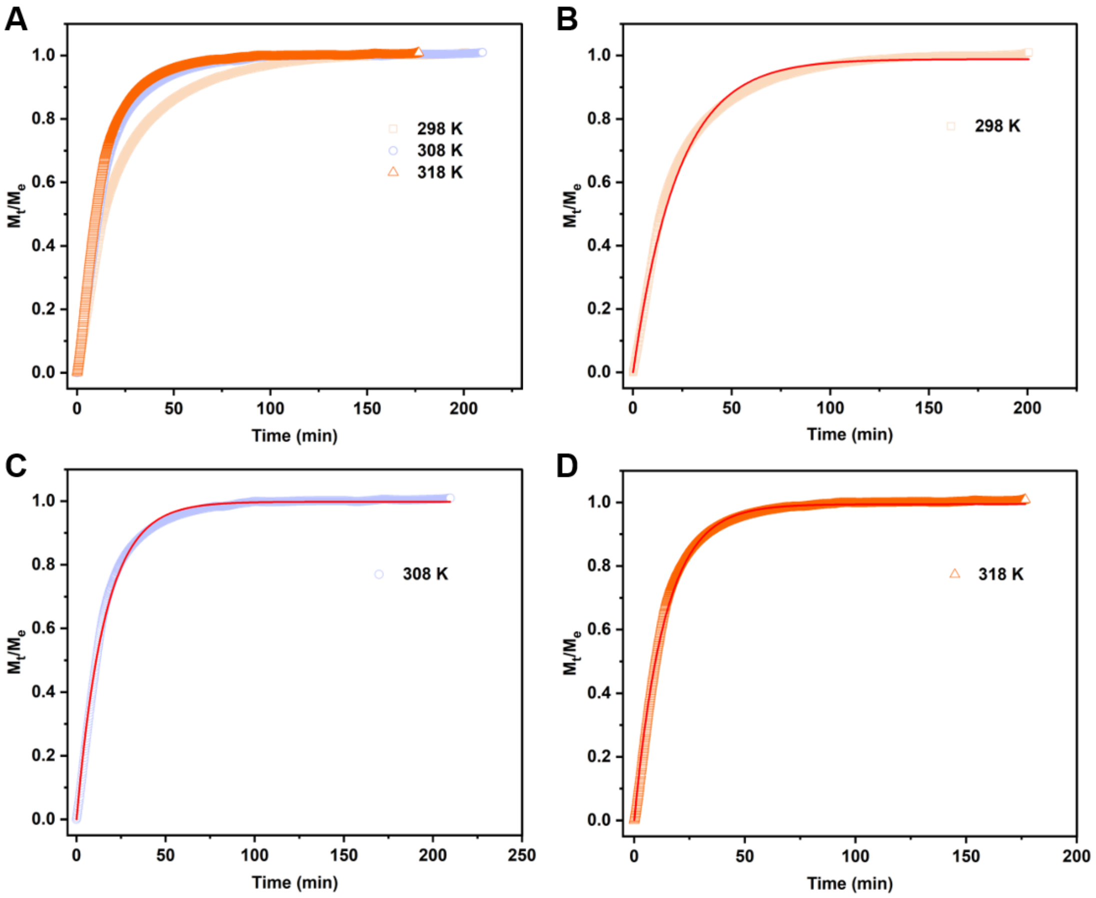

Figure S46. The kinetic profiles and fitting results at 0-5 mbar. The kinetic profiles at different temperatures (**A**), and the fitting results at 298K (**B**), 308K (**C**), 318K (**D**).

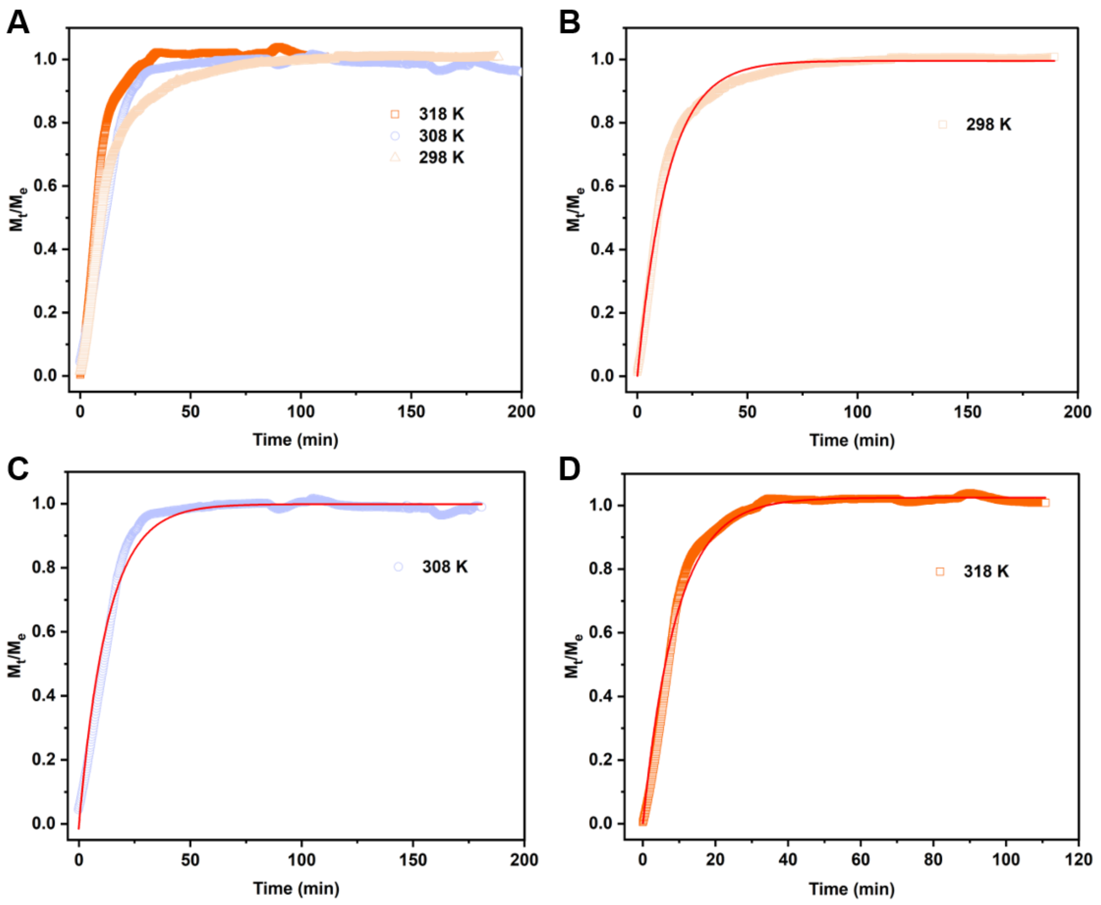

Figure S47. The kinetic profiles and fitting results at 5-10 mbar. The kinetic profiles at different temperatures (**A**), and the fitting results at 298K (**B**), 308K (**C**), 318K (**D**).

Table S23. Fitting results of vapour adsorption kinetic.

P (mbar)	T (K)	A	*k* (s)	R^2
0-5	298	0.988	0.0443	0.993
	308	0.997	0.0629	0.995
	318	0.994	0.0730	0.995
5-10	298	0.995	0.0729	0.987
	308	0.998	0.0779	0.976
	318	1.024	0.1091	0.983

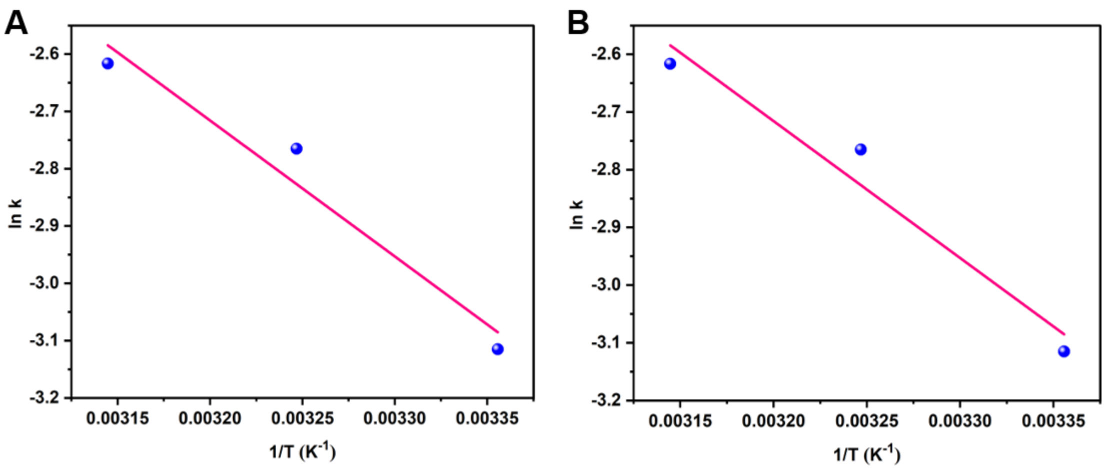

Figure S48. Plots of ln(*k*) against the reciprocal of T at 0-5 mbar (**A**) and 5-10 mbar (**B**).

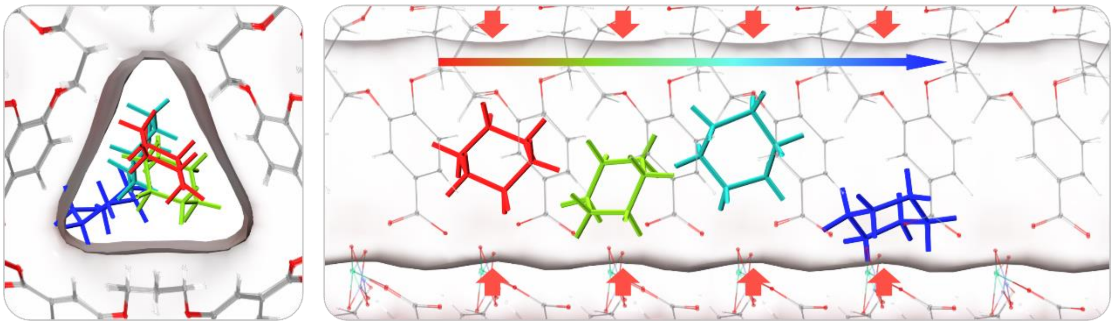

Figure S49. The configuration of cyclohexane passing through the NKU-300 channel.

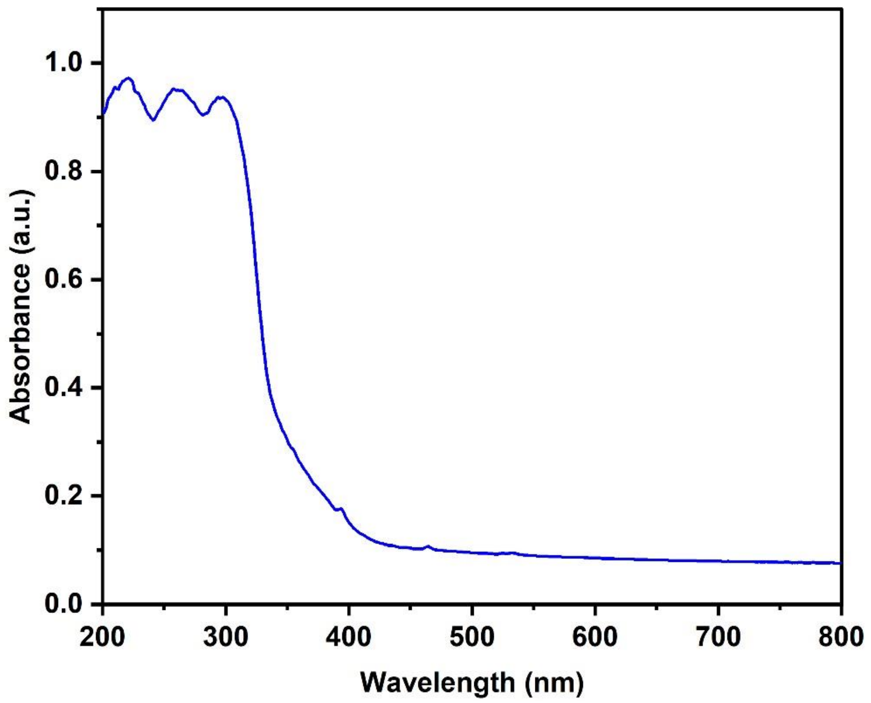

Figure S50. The solid-state UV-Vis diffuse reflectance spectrum of NKU-300.

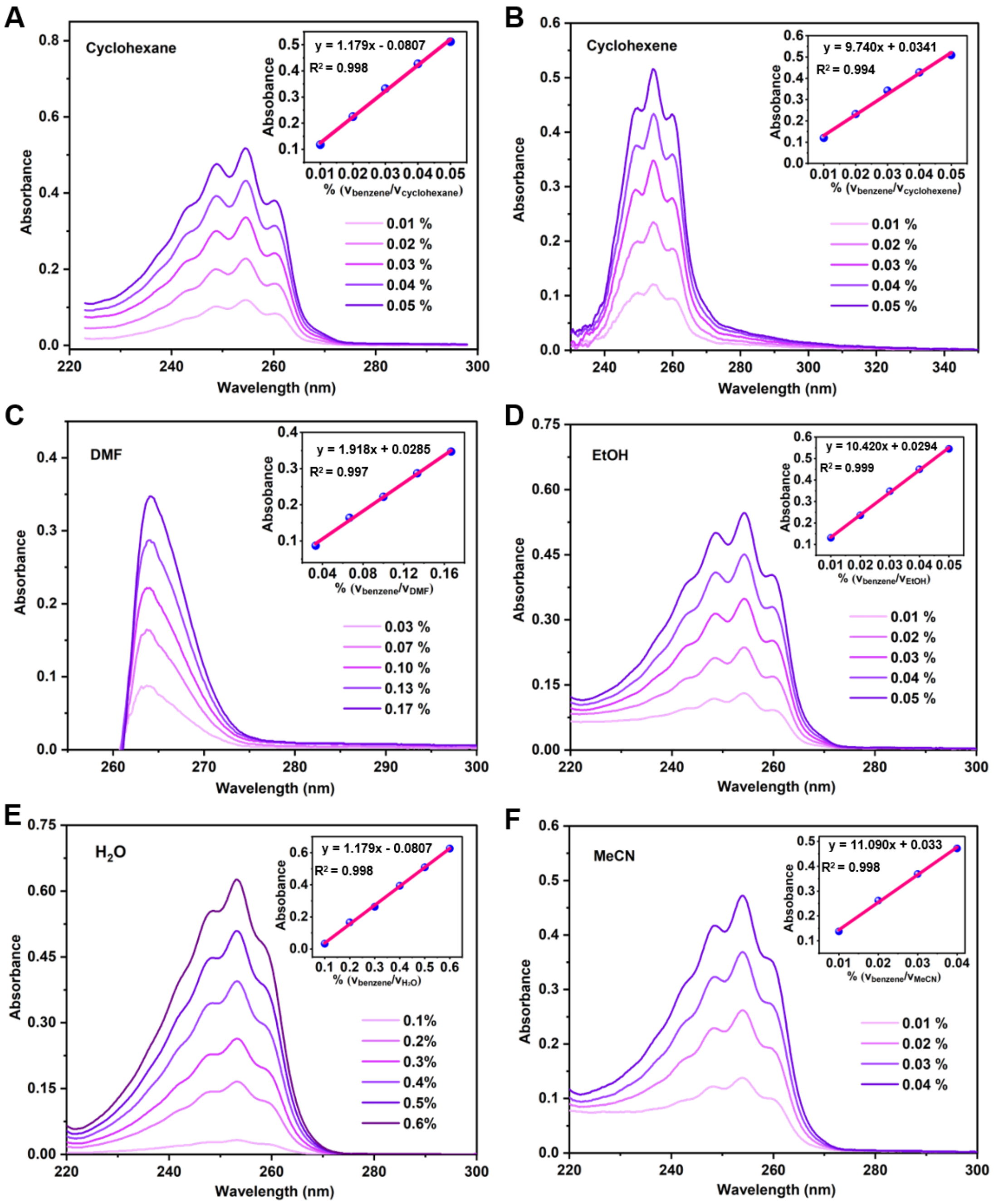

Figure S51. Liquid UV-vis spectra and fitting results. Liquid UV-vis spectra of benzene in cyclohexane (**A**), cyclohexene (**B**), DMF (**C**), EtOH (**D**), H_2O (**E**), and MeCN (**F**).

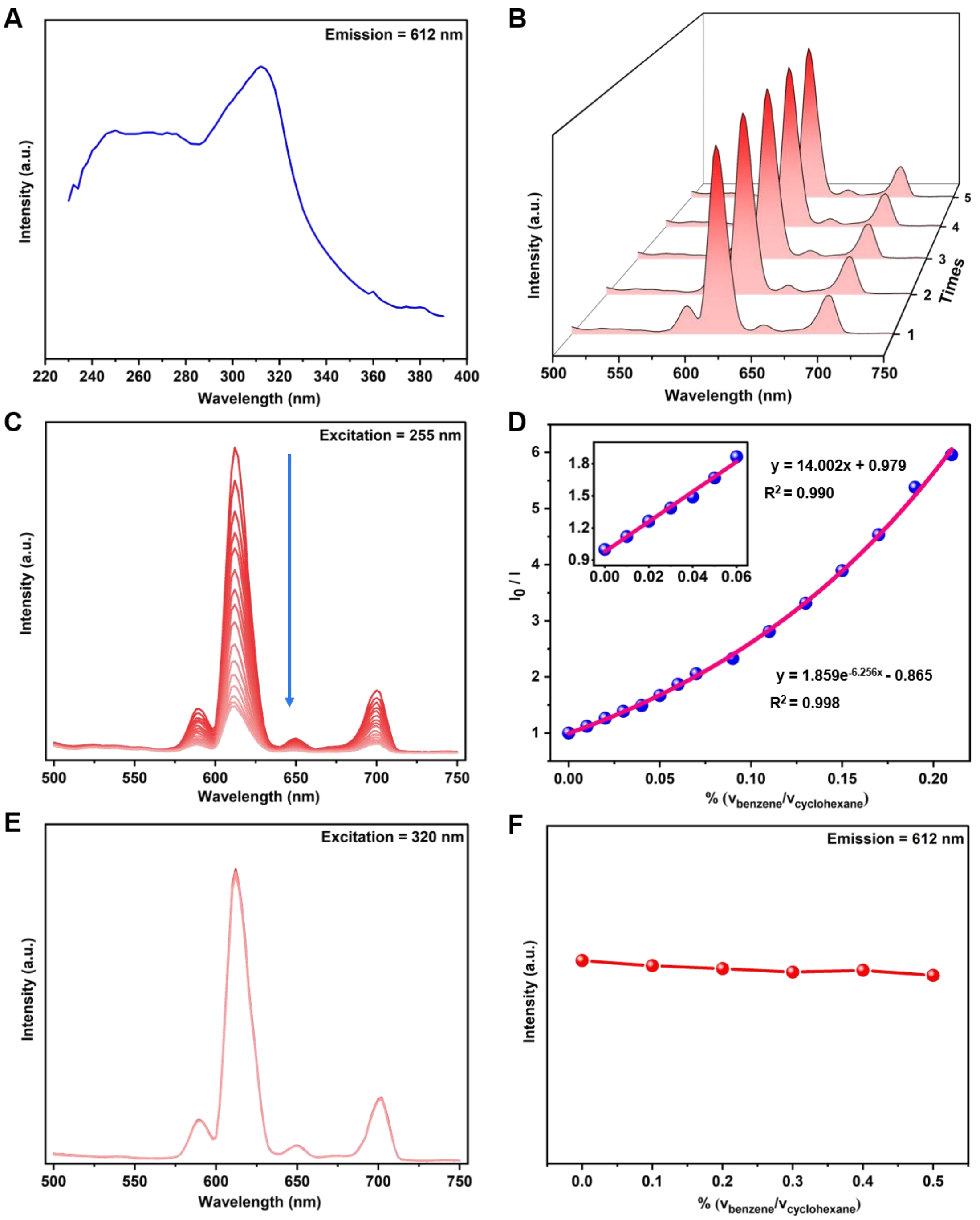

Figure S52. Sensing performance of NKU-300 for benzene in cyclohexane. (**A**) Excitation spectrum of NKU-300 dispersed in cyclohexane. (**B**) NKU-300 suspension in cyclohexane for 5 minutes. (**C**) Fluorescence emission spectra of NKU-300 dispersed in cyclohexane upon incremental addition of benzene (Excitation at 255 nm). (**D**) Fluorescence intensity changes of NKU-300 at 612 nm (Excitation at 255 nm). (**E**) Fluorescence emission spectra of NKU-300 dispersed in cyclohexane upon incremental addition of benzene (Excitation at 320 nm). (**F**) Fluorescence intensity changes of NKU-300 at 612 nm (Excitation at 320 nm).

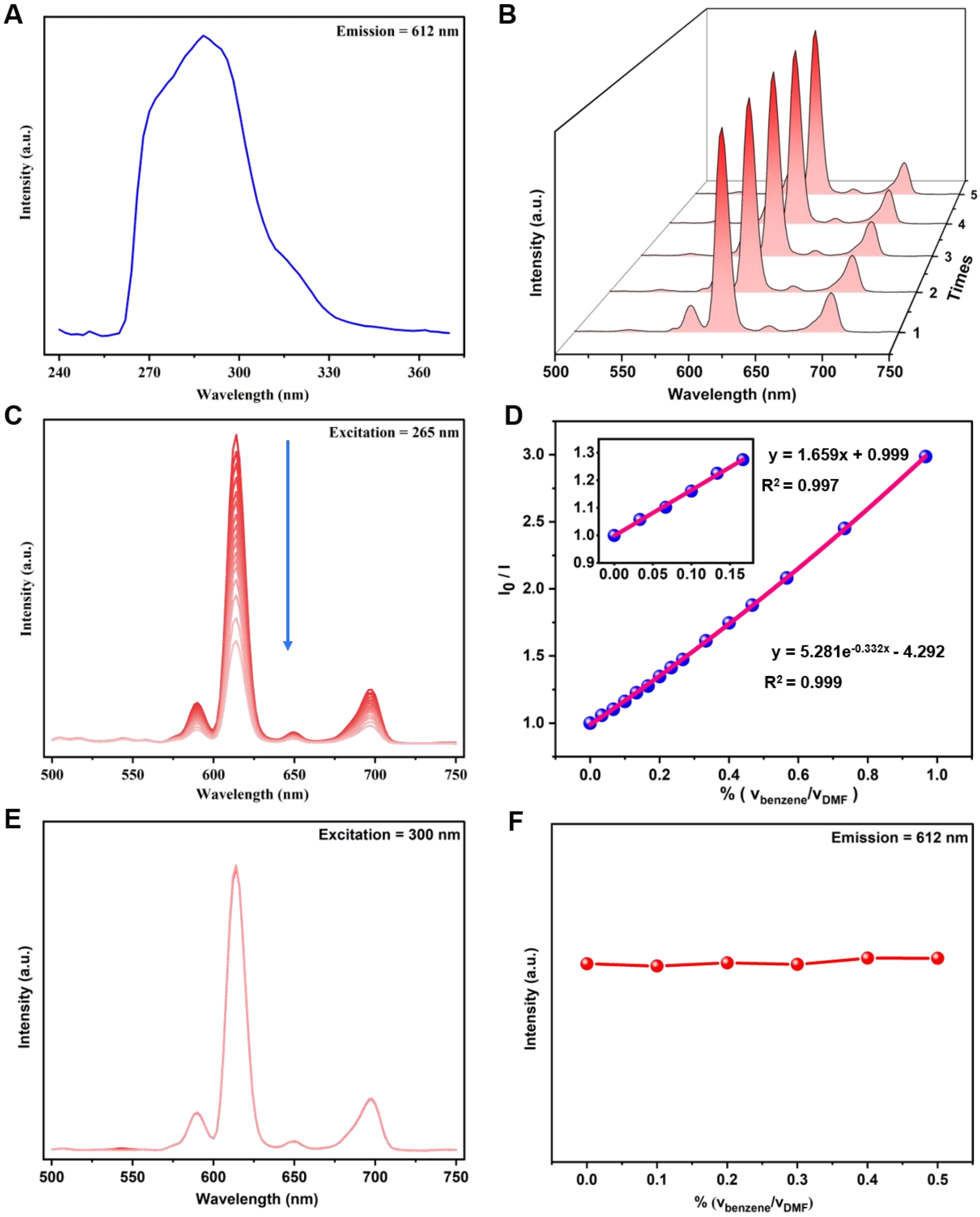

Figure S53. Sensing performance of NKU-300 for benzene in DMF. (**A**) Excitation spectrum of NKU-300 dispersed in DMF. (**B**) NKU-300 suspension in DMF for 5 minutes. (**C**) Fluorescence emission spectra of NKU-300 dispersed in DMF upon incremental addition of benzene (Excitation at 265 nm). (**D**) Fluorescence intensity changes of NKU-300 at 612 nm (Excitation at 265 nm). (**E**) Fluorescence emission spectra of NKU-300 dispersed in DMF upon incremental addition of benzene (Excitation at 300 nm). (**F**) Fluorescence intensity changes of NKU-300 at 612 nm (Excitation at 300 nm).

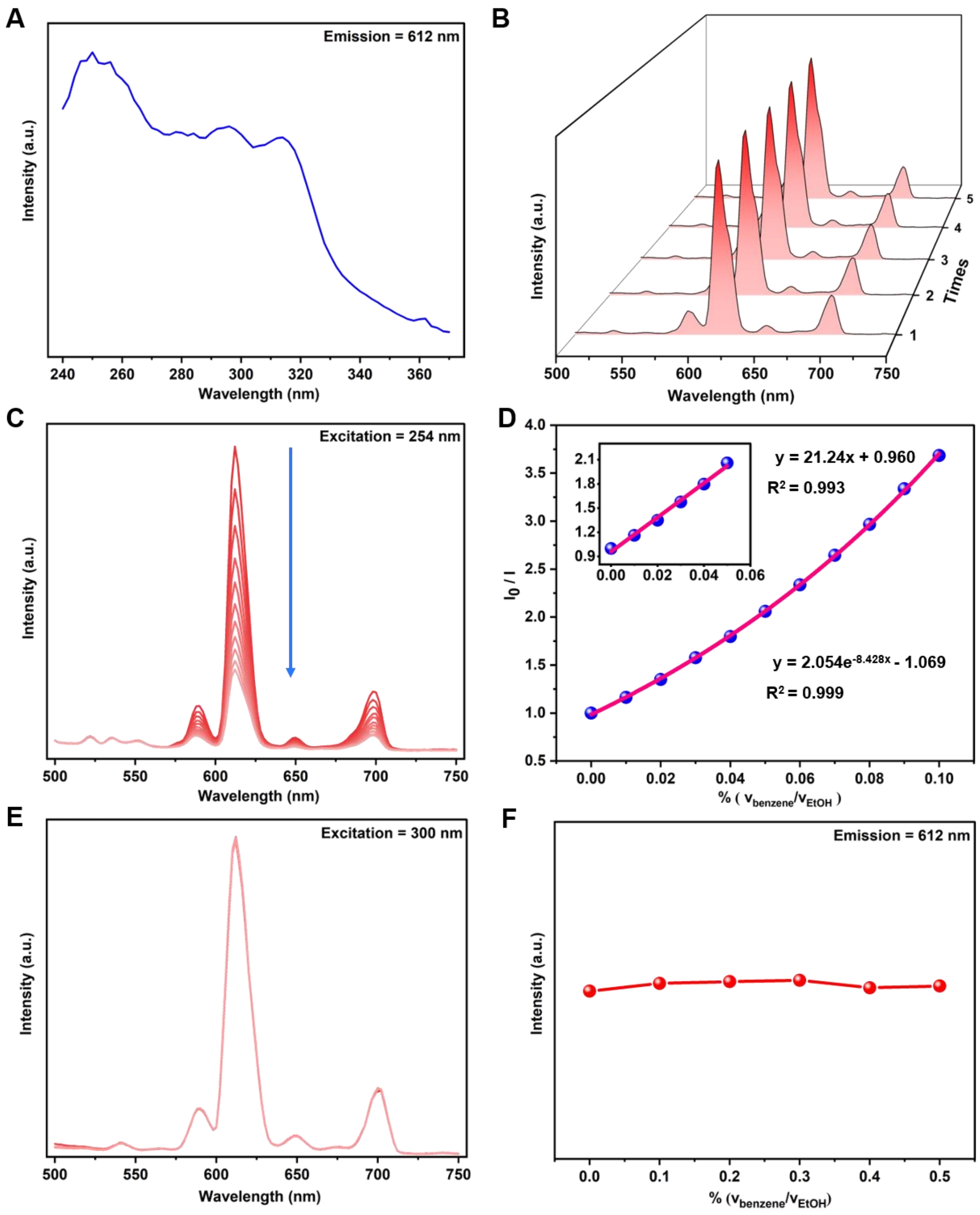

Figure S54. Sensing performance of NKU-300 for benzene in EtOH. (**A**) Excitation spectrum of NKU-300 dispersed in EtOH. (**B**) NKU-300 suspension in EtOH for 5 minutes. (**C**) Fluorescence emission spectra of NKU-300 dispersed in EtOH upon incremental addition of benzene (Excitation at 254 nm). (**D**) Fluorescence intensity changes of NKU-300 at 612 nm (Excitation at 254 nm). (**E**) Fluorescence emission spectra of NKU-300 dispersed in EtOH upon incremental addition of benzene (Excitation at 300 nm). (**F**) Fluorescence intensity changes of NKU-300 at 612 nm (Excitation at 300 nm).

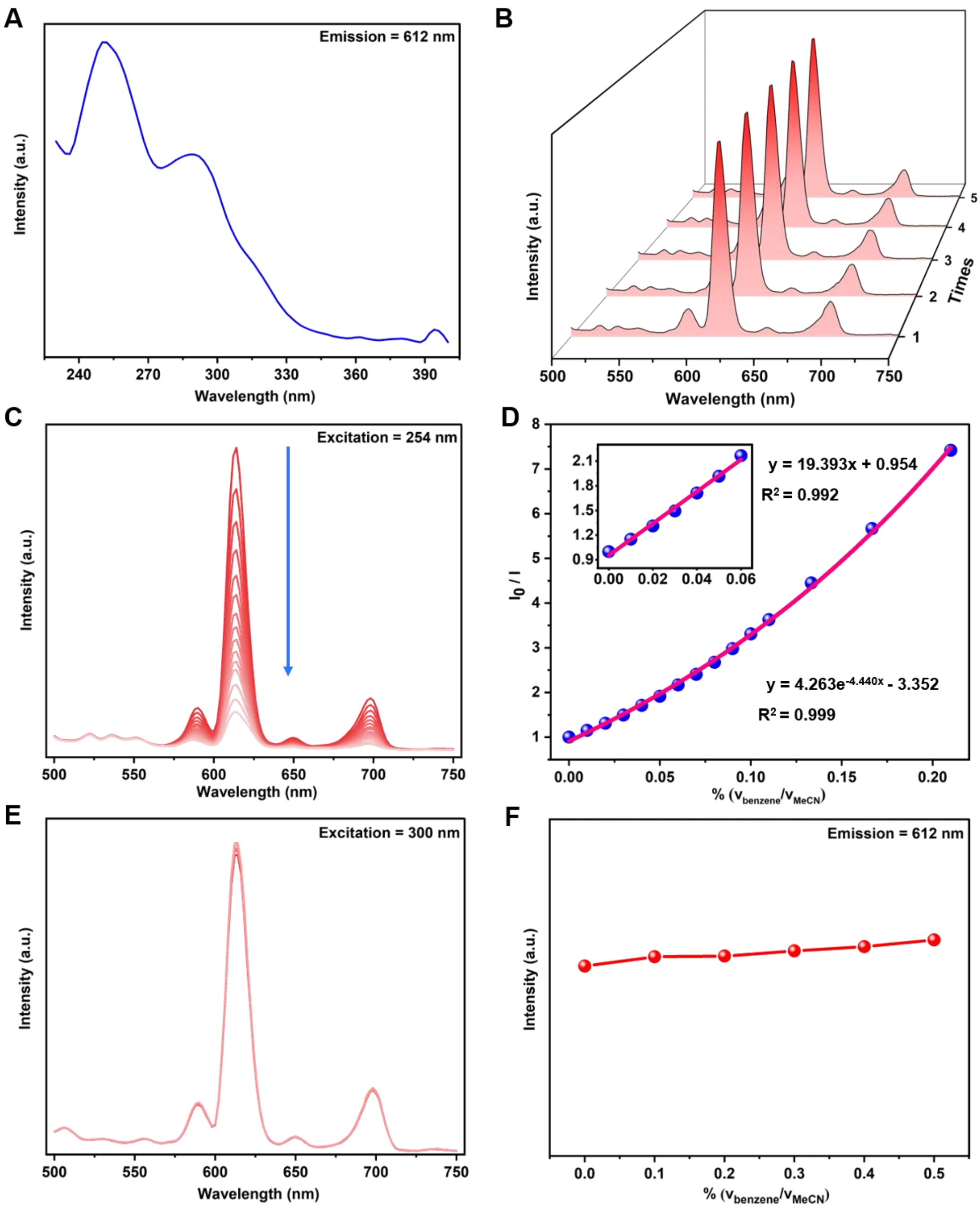

Figure S55. Sensing performance of NKU-300 for benzene in MeCN. (**A**) Excitation spectrum of NKU-300 dispersed in MeCN. (**B**) NKU-300 suspension in MeCN for 5 minutes. (**C**) Fluorescence emission spectra of NKU-300 dispersed in MeCN upon incremental addition of benzene (Excitation at 254 nm). (**D**) Fluorescence intensity changes of NKU-300 at 612 nm (Excitation at 254 nm). (**E**) Fluorescence emission spectra of NKU-300 dispersed in MeCN upon incremental addition of benzene (Excitation at 300 nm). (**F**) Fluorescence intensity changes of NKU-300 at 612 nm (Excitation at 300 nm).

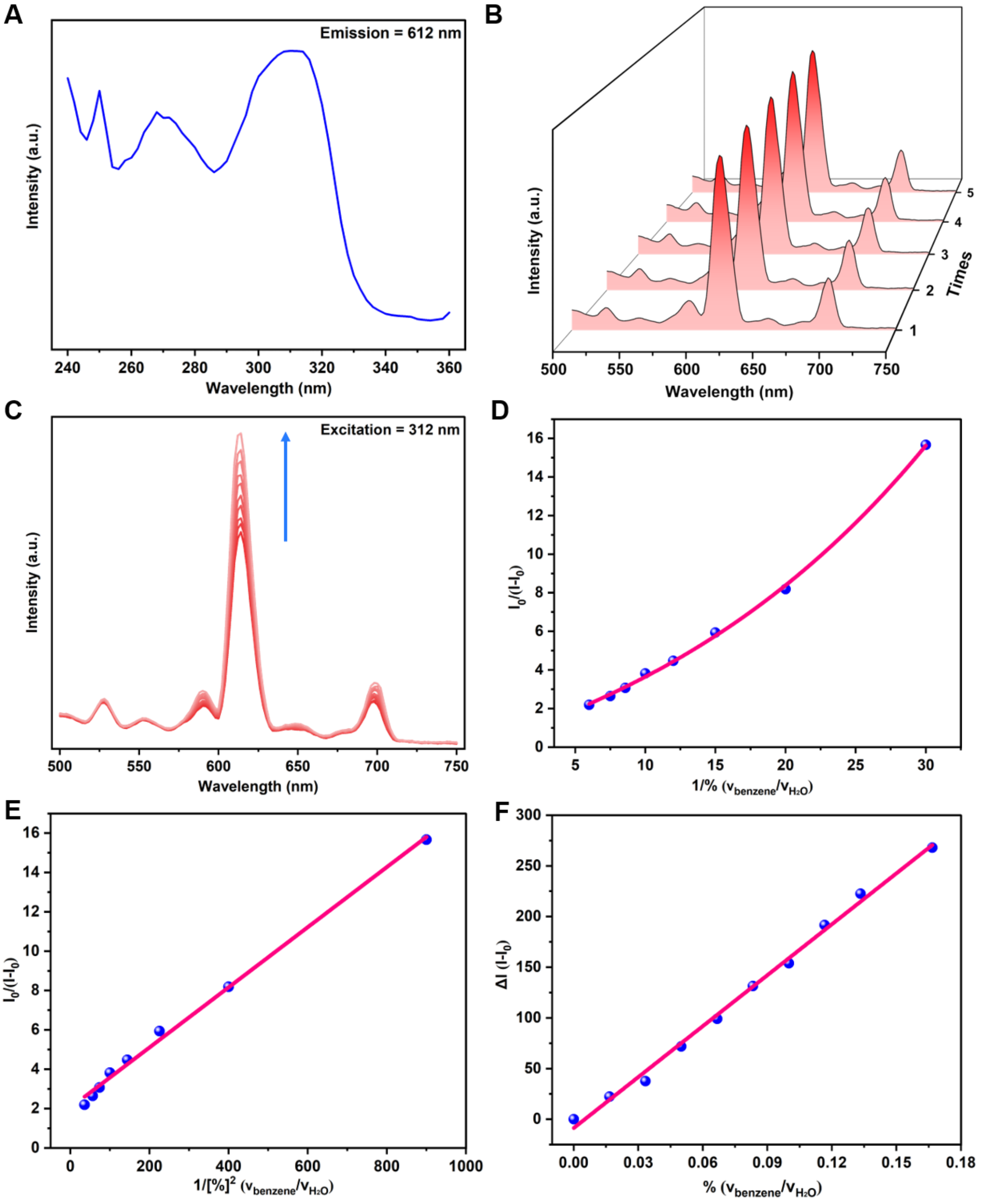

Figure S56. Sensing performance of NKU-300 for benzene in H_2O. (**A**) Excitation spectrum of NKU-300 dispersed in H_2O. (**B**) NKU-300 suspension in H_2O for 5 minutes. (**C**) Fluorescence emission spectra of NKU-300 dispersed in H_2O upon incremental addition of benzene (Excitation at 312 nm). (**D**) Fluorescence intensities of NKU-300 at 612 nm vs the reciprocal of benzene volume fraction in H_2O. (**E**) Fluorescence intensities of NKU-300 at 612 nm vs the reciprocal of benzene volume fraction squared in H_2O. (**F**) Plots of ΔI ($I - I_0$) against benzene volume fraction in H_2O.

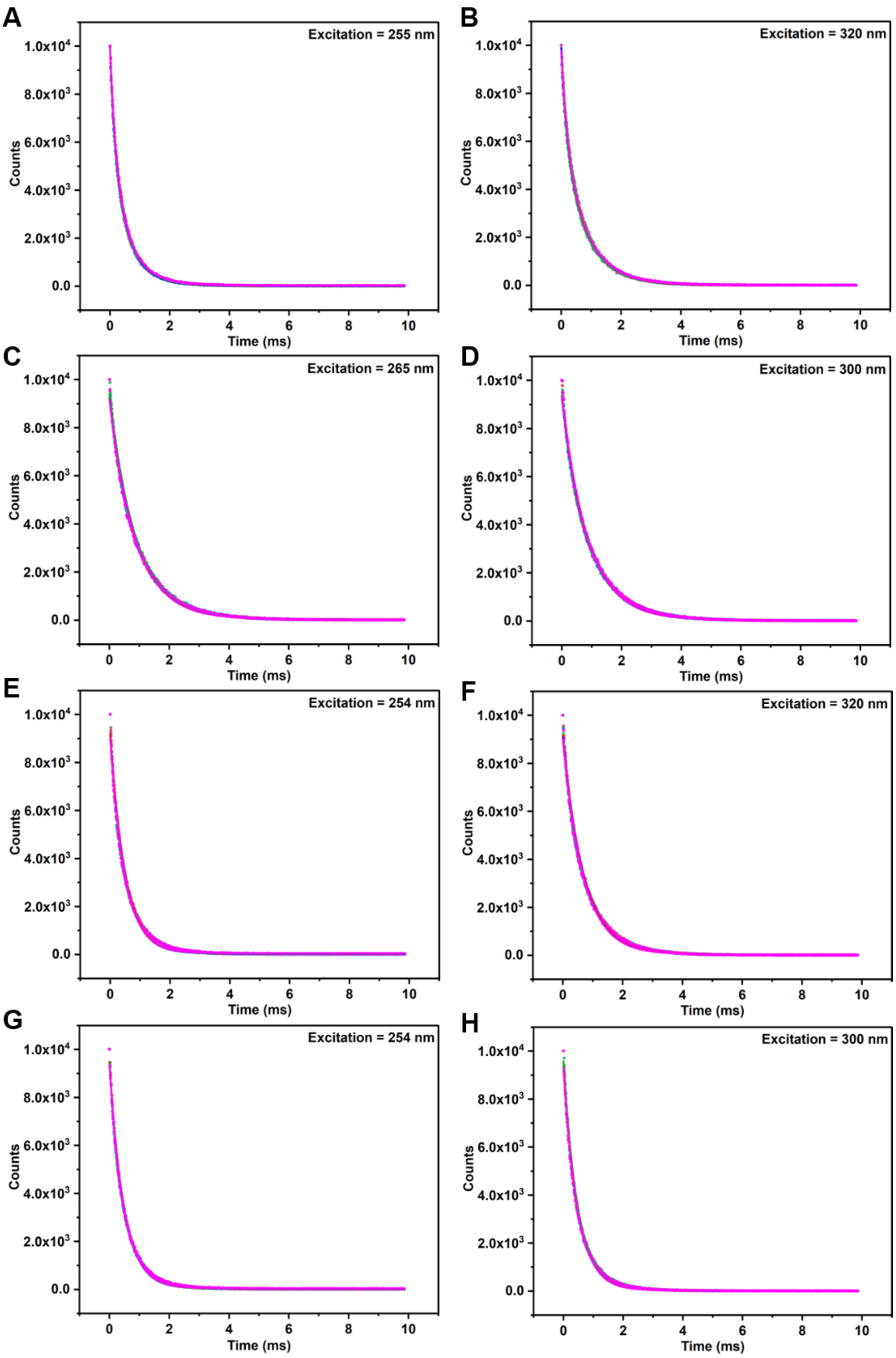

Figure S57. Luminescence lifetime patterns at 612 nm. NKU-300 dispersed in cyclohexane in the presence of benzene excited at 255 nm (**A**) and 320 nm (**B**). NKU-300 dispersed in DMF in the presence of benzene excited at 265 nm (**C**) and 300 nm (**D**). NKU-300 dispersed in EtOH in the presence of benzene excited at 254 nm (**E**) and 320 nm (**F**). NKU-300 dispersed in MeCN in the presence of benzene excited at 254 nm (**G**) and 300 nm (**H**).

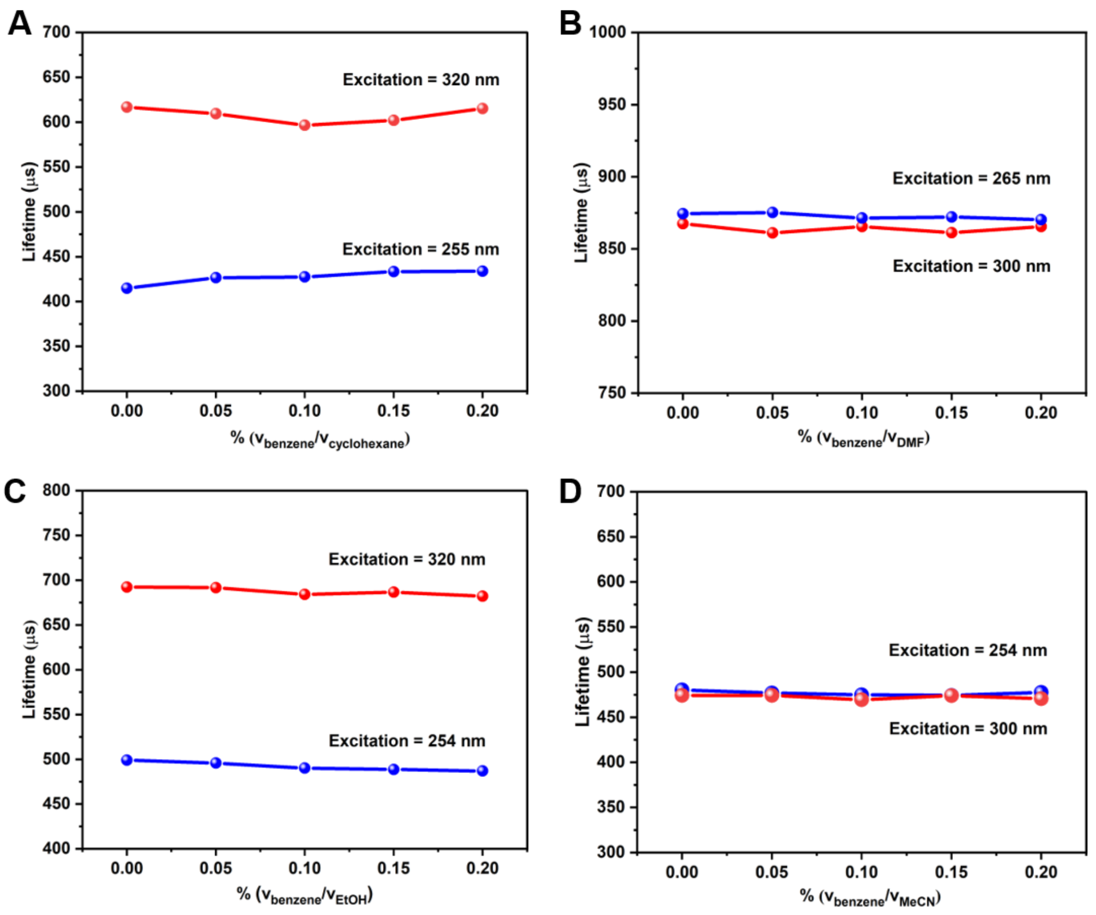

Figure S58. Lifetime fitting results. (**A**) NKU-300 dispersed in cyclohexane in different percent of benzene. (**B**) NKU-300 dispersed in DMF in different percent of benzene. (**C**) NKU-300 dispersed in EtOH in different percent of benzene. (**D**) NKU-300 dispersed in MeCN in different percent of benzene.

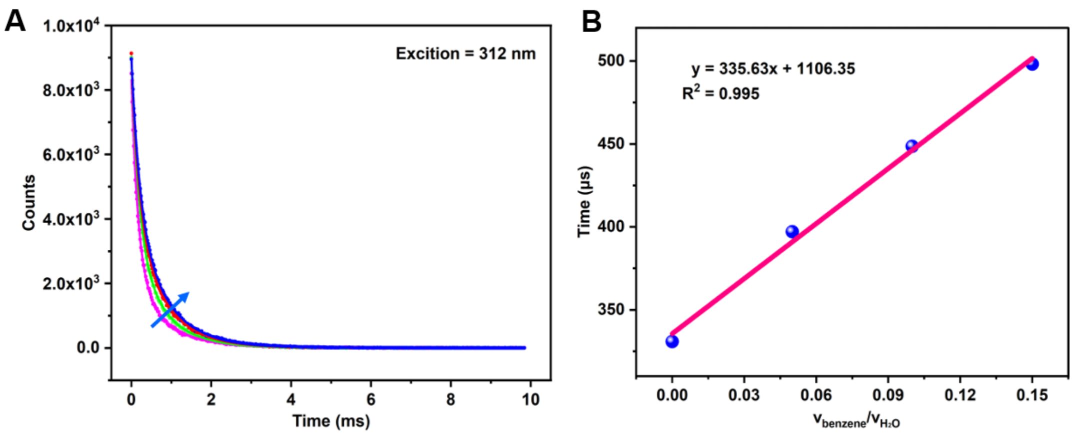

Figure S59. Luminescence lifetime pattern at 612 nm and fitting results. (**A**) Luminescence lifetimes of NKU-300 dispersed in H_2O in the presence of benzene excited at 312 nm. (**B**) Lifetime fitting results of NKU-300 dispersed in H_2O in different percent of benzene.

Table S24. Fitting results of the luminescence experiments.

Solvent	Linear Range (%)	K_{SV}	K_{BH}	LOD (%)
Cyclohexane	0.00001-0.060	1.40×10^{3}	/	0.30×10^{-2}
DMF	0.00001-0.170	1.66×10^{2}	/	1.40×10^{-2}
EtOH	0.00001-0.050	2.12×10^{3}	/	1.40×10^{-3}
MeCN	0.00001-0.060	1.94×10^{3}	/	0.50×10^{-3}
H_2O	0.00001-0.167	/	6.54×10^{4}	0.74×10^{-5}

NMR Spectra for New Compounds

4,4'-(propane-1,3-diylbis(oxy))bis(3-hydroxybenzoic acid) (4)

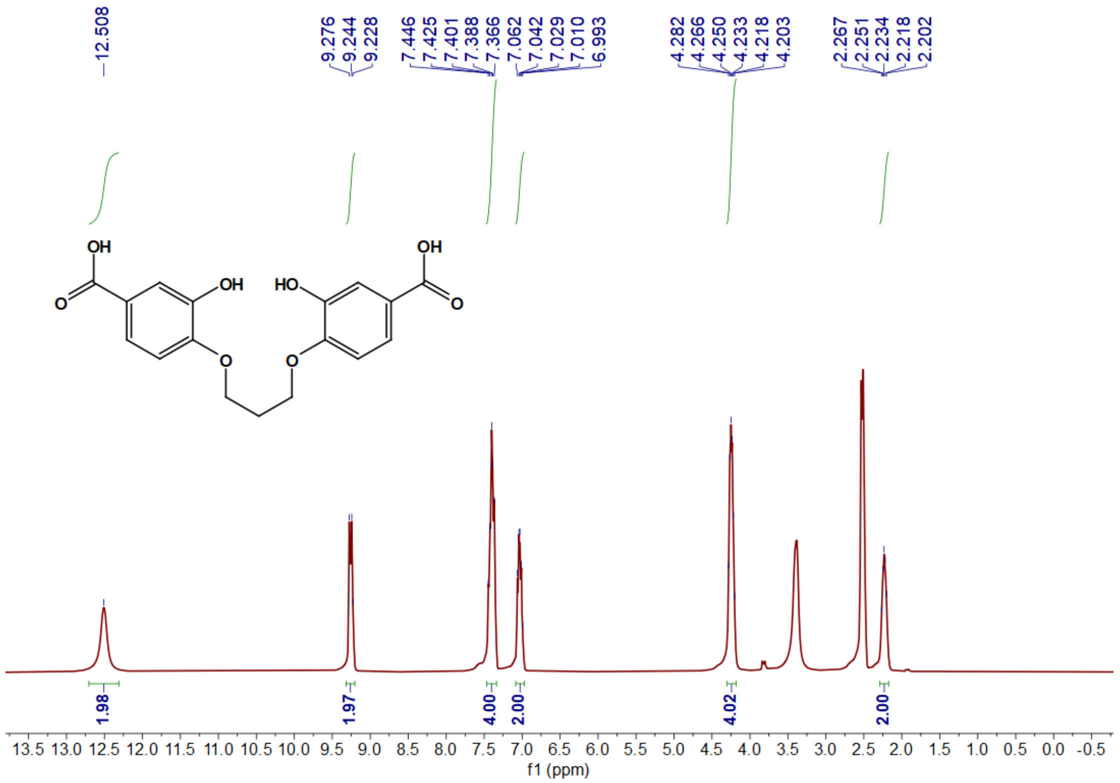

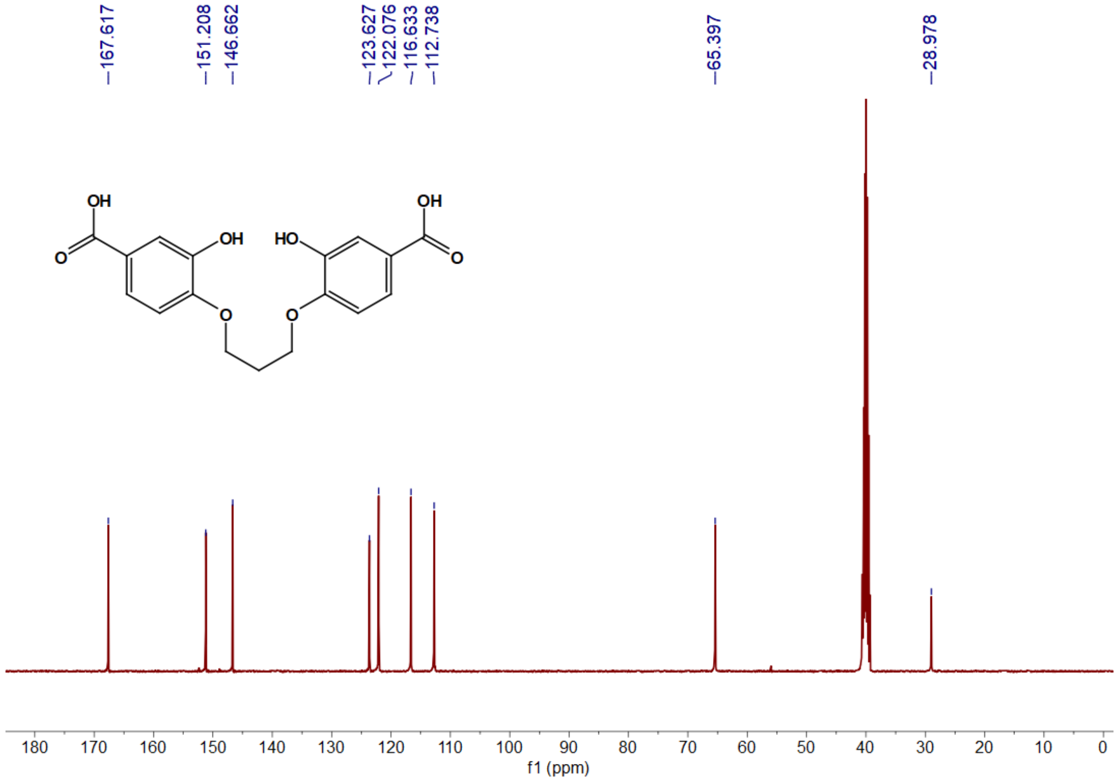

dimethyl 4,4'-(propane-1,3-diylbis(oxy))bis(3-hydroxybenzoate) (5)

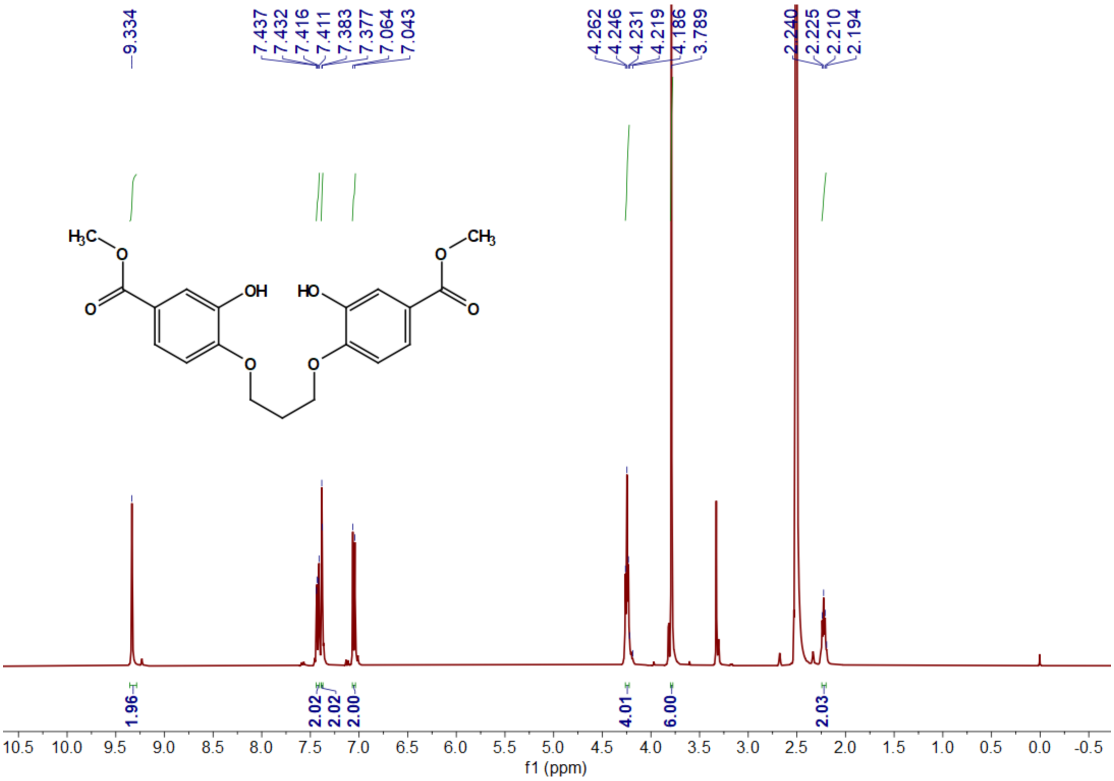

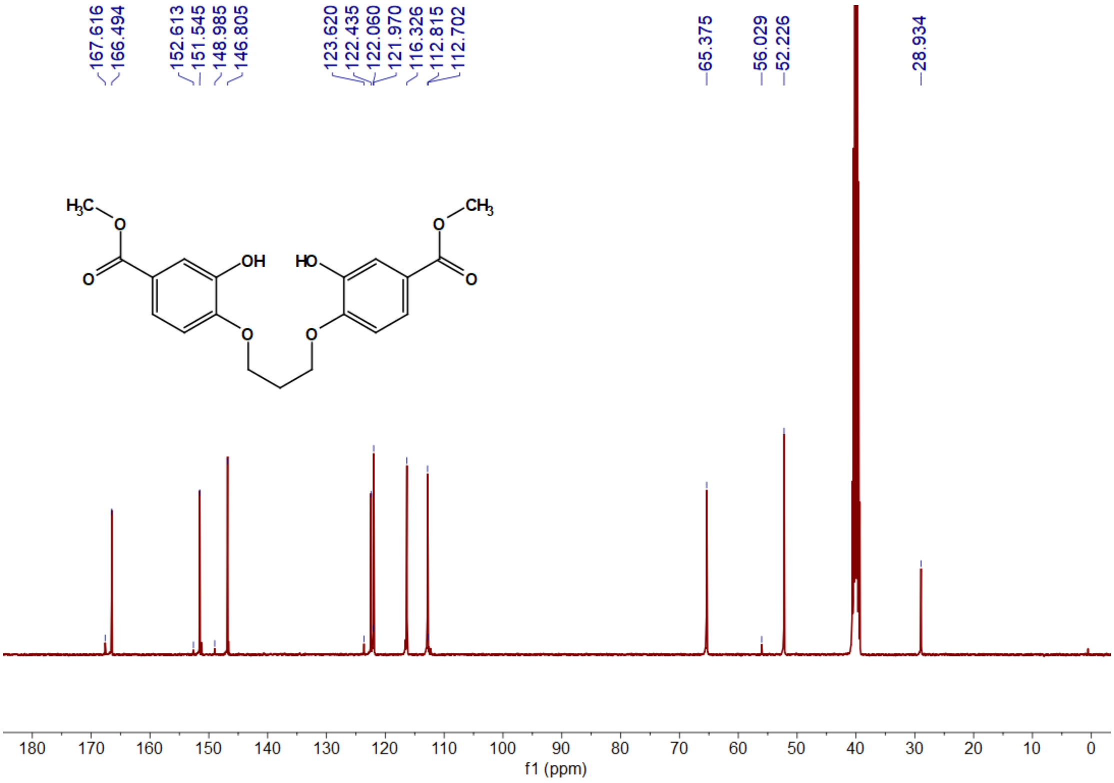

dimethyl 7,8,16,17-tetrahydro-6*H*,15*H*-dibenzo[*b*,*i*][1,4,8,11]tetraoxacyclotetradecine-2,12-dicarboxylate (6)

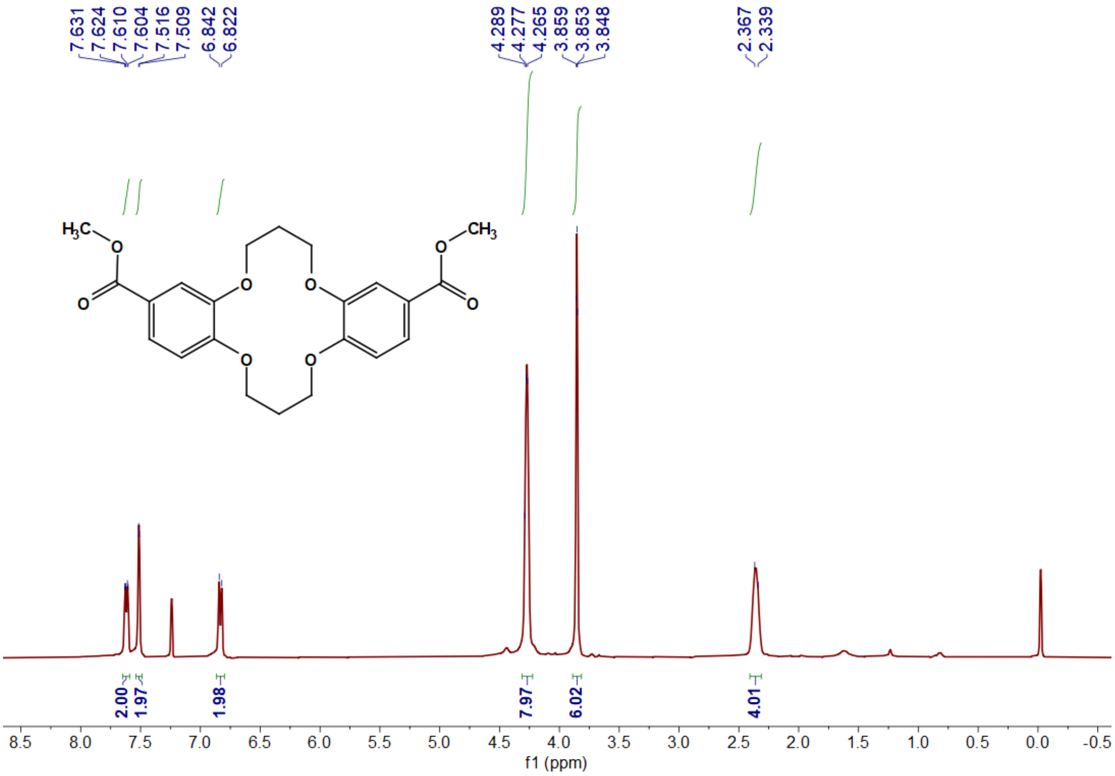

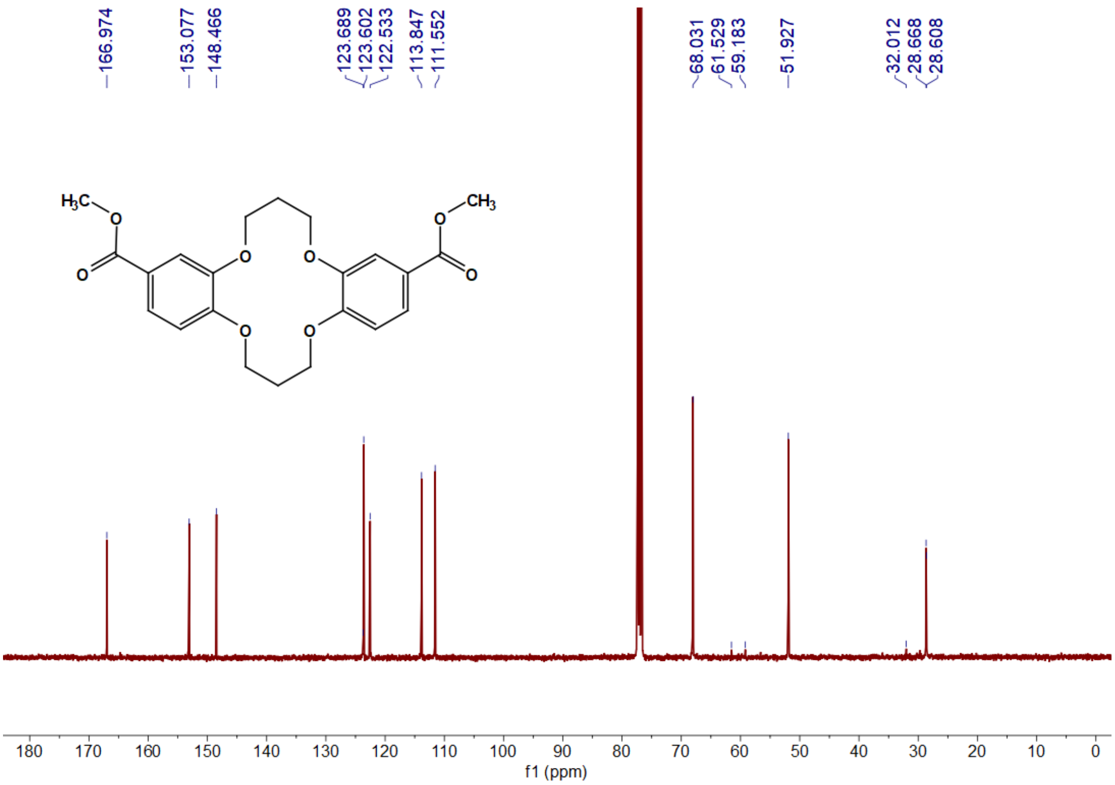

7,8,16,17-tetrahydro-6*H*,15*H*-dibenzo[*b*,*i*][1,4,8,11]tetraoxacyclotetradecine-2,12-dicarboxylic acid (H_2L)

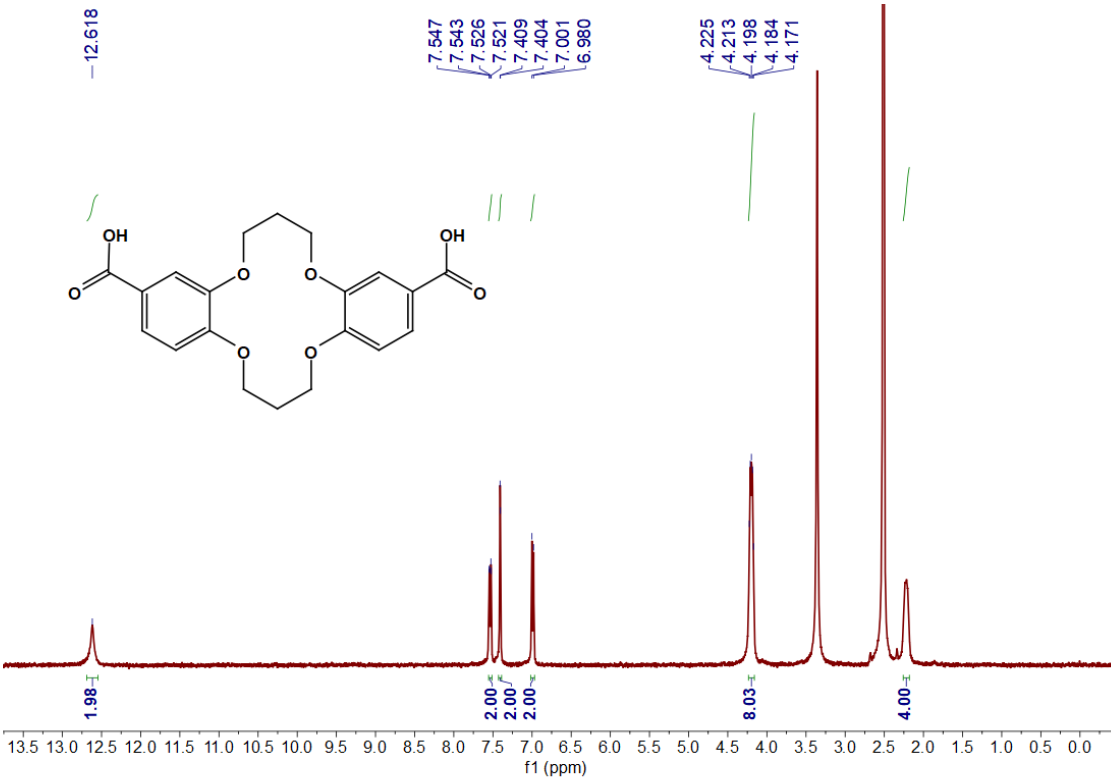

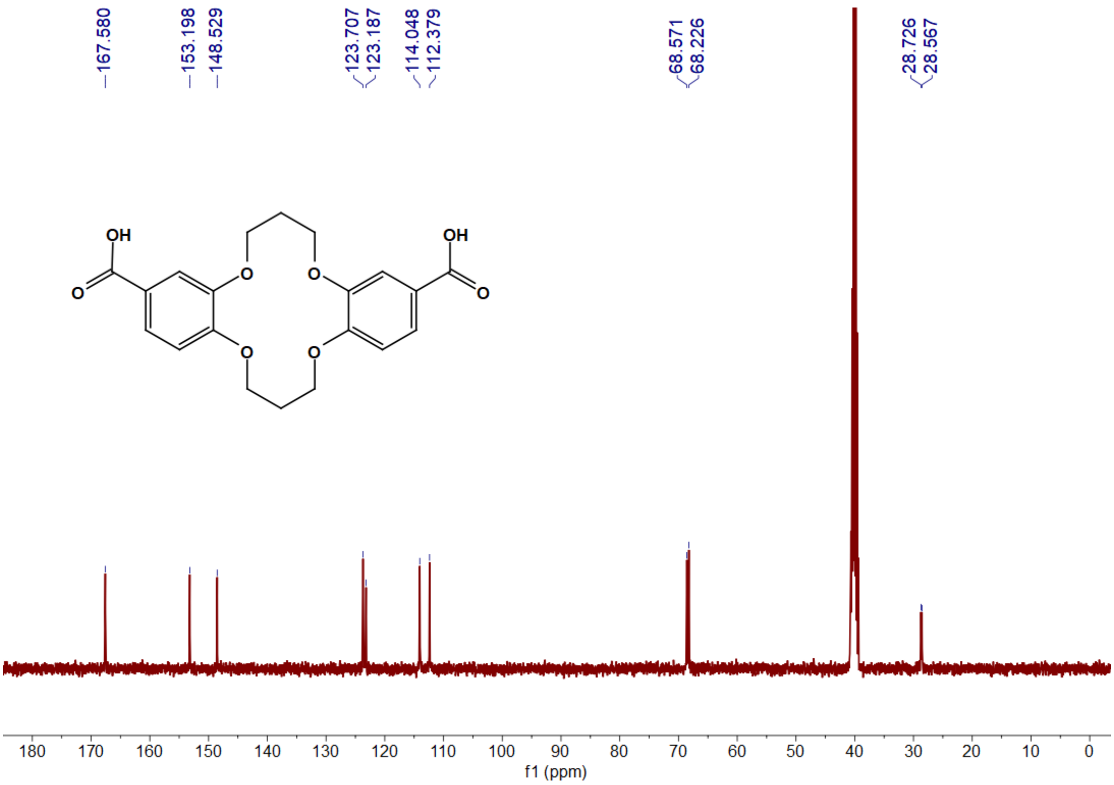

References

1. Bell TW, Choi H-J and Harte W *et al*. Syntheses, conformations, and basicities of bicyclic triamines. *J Am Chem Soc* 2003; **125**: 12196–210.
2. Fletcher AJ, Cussen EJ and Bradshaw D *et al*. Adsorption of gases and vapors on nanoporous Ni_2(4,4′-Bipyridine)$_3$(NO_3)$_4$ metal-organic framework materials templated with methanol and ethanol: Structural effects in adsorption kinetics. *J Am Chem Soc* 2004; **126**: 9750−9.
3. Sheldrick GM. SHELXT - Integrated space-group and crystals-structure determination. *Acta Cryst* 2015; **A71**: 3–8.
4. Sheldrick GM. Crystal structure refinement with SHELXL. *Acta Cryst* 2015; **C71**: 3–8.
5. Frisch MJ, Trucks GW and Schlegel HB *et al*. Gaussian 16, Revision A.03, Gaussian, Inc., Wallingford CT, 2016.
6. Lu T and Chen F. Multiwfn: A multifunctional wavefunction analyzer. *J Comput Chem* 2012; **33**: 580–92.
7. Humphrey W, Dalke A and Schulten K. VMD: Visual molecular dynamics. *J Mol Graphics* 1996; **14**: 33–8.
8. Bannwarth C, Caldeweyher E and Ehlert S *et al*. Extended tight-binding quantum chemistry methods. *WIREs Comput Mol Sci* 2020; **11**: e01493.
9. Spicher S and Grimme S. Robust atomistic modeling of materials, organometallic, and biochemical systems. *Angew Chem Int Ed* 2020; **59**: 15665–73.
10. Zhou J, Yu G and Li Q *et al*. Separation of benzene and cyclohexane by nonporous adaptive crystals of a Hybrid[3]arene. *J Am Chem Soc* 2020; **142**: 2228–32.
11. Yao L-Y, and Yam VW-W. Dual emissive gold(I)–sulfido cluster framework capable of benzene–cyclohexane separation in the solid state accompanied by luminescence color changes. *J Am Chem Soc* 2021; **143**: 2558–66.
12. Cui P-F, Liu X-R and Lin Y-J *et al*. Highly selective separation of benzene and cyclohexane in a spatially confined carborane metallacage. *J Am Chem Soc* 2022; **144**: 6558–65.
13. Ye C-R, Wang W-J and Chen W *et al*. Harnessing shape complementarity for upgraded cyclohexane purification through adaptive bottlenecked pores in an imidazole-containing MOF. *Angew Chem Int Ed* 2021; **60**: 23590–5.
14. Han Y, Chen Y and Ma Y *et al*. Control of the pore chemistry in metal-organic frameworks for efficient adsorption of benzene and separation of benzene/cyclohexane. *Chem* 2023; **9**: 739–54.
15. Lysova AA, Samsonenko DG and Dorovatovskii PV *et al*. Tuning the molecular and cationic affinity in a series of multifunctional metal–organic frameworks based on dodecanuclear Zn(II) carboxylate wheels. *J Am Chem Soc* 2019; **141**: 17260–9.
16. Liu C-H, Chen L and Zhang H *et al*. A B←N framework based on 1D dative B←N polymers for exclusive recognition and separation of benzene from its azeotrope. *Chem* 2023; **9**: 3532–43.
17. Li G, Zhu C and Xi X *et al*. Selective binding and removal of organic molecules in a flexible polymeric material with stretchable metallosalen chains. *Chem Commun* 2009; **16**: 2118–20.

18. Sapianik AA, Kovalenko KA and Samsonenko DG *et al*. Exceptionally effective benzene/cyclohexane separation using a nitro-decorated metal–organic framework. *Chem Commun* 2020; **56**: 8241–4.
19. Cox EG and Smith JAS. Crystal Structure of Benzene at −3°C. *Nature* 1954; **173**: 75.
20. Cox EG. Crystal structure of benzene. *Rev Mod Phys* 1958; **30**: 159–62.
